# Identification and Validation of Novel Immune-Related Alternative Splicing Signatures as a Prognostic Model for Colon Cancer

**DOI:** 10.3389/fonc.2022.866289

**Published:** 2022-05-26

**Authors:** Yunze Liu, Lei Xu, Chuanchuan Hao, Jin Wu, Xianhong Jia, Xia Ding, Changwei Lin, Hongmei Zhu, Yi Zhang

**Affiliations:** ^1^Department of Traditional Chinese Medicine, Affiliated Hospital of Xuzhou Medical University, Xuzhou, China; ^2^Dongzhimen Hospital, Beijing University of Chinese Medicine, Beijing, China; ^3^Department of General Surgery, Affiliated Hospital of Xuzhou Medical University, Xuzhou, China; ^4^Institute of Digestive Diseases, Xuzhou Medical University, Xuzhou, China; ^5^Department of Gastrointestinal Surgery, The Third Xiang Ya Hospital of Central South University, Changsha, China

**Keywords:** bioinformatic analysis, alternative splicing, immune, colon cancer, prognosis

## Abstract

**Background:**

Individual immune-related alternative splicing (AS) events have been found to be significant in immune regulation and cancer prognosis. However, a comprehensive analysis of AS events in cancer cells based on immune-related genes (IRGs) has not been performed, and its clinical value is unknown.

**Methods:**

Colon cancer cases with AS data were obtained from TCGA, and then, we identified overall survival-related AS events (OS-ASEs) based on IRGs by univariate analyses. Using Lasso regression, multivariate Cox regression, Kaplan–Meier analysis and nomograms, we constructed an AS risk model based on the calculated risk score. Furthermore, associations of the risk score with clinical and immune features were confirmed through the Wilcoxon rank sum test, association analysis, etc. Finally, by qRT–PCR, cell coculture and CCK-8 analyses, we validated the significance of OS-ASEs in colon cancer cell lines and clinical samples.

**Results:**

A total of 3,119 immune-related AS events and 183 OS-ASEs were identified, and 9 OS-ASEs were ultimately used to construct a comprehensive risk model for colon cancer patients. Low-risk patients had better OS and DFS rates than high risk patients. Furthermore, a high risk score corresponded to high numbers of multiple tumour-infiltrating immune cells and high expression of HLA-D region genes and immune checkpoint genes. Notably, we identified for the first time that anti-PD-L1 or anti-CTLA-4 antibodies may decrease the OS of specific colon cancer patients in the low-risk group. Additionally, the *in vitro* experiment validated that CD46-9652-ES and PSMC5-43011-ES are positively correlated with the infiltration of immune cells and promote the growth of colon cancer cells. CD46-9652-ES can contribute to T cell-mediated tumour cell killing. PSMC5-43011-ES was observed to induce M2 polarization of macrophages.

**Conclusions:**

This study identified and validated immune-related prognostic AS signatures that can be used as a novel AS prognostic model and provide a novel understanding of the relationship between the immune microenvironment and clinical outcomes.

## Introduction

Colon cancer is one of the most common malignancies worldwide. Its incidence and mortality rate have both continuously increased in recent years (1–3). The 5-year overall survival (OS) for persons with colon cancer is approximately 65% in the United States (4). Effective prognostic evaluation is necessary to provide precise and personalized treatment for colon cancer patients and improve patient outcomes. In recent years, tumour-node-metastasis (TNM) pathological staging has been recommended as a common staging method (5, 6). Patients with different TNM stages have approximately distinguishing prognoses. However, individual differences in colon cancer patients within the same TNM pathological stage cause significant differences in OS and recurrence outcomes (7). Thus, it is necessary to develop a meaningful prognostic model based on molecular signatures to improve the accuracy of prediction and treatment strategies for colon cancer.

Alternative splicing (AS) is a common process by which a pre-messenger RNA (pre-mRNA) can be spliced at different sites to produce at least two different mRNA splicing isoforms, increasing protein diversity (8). Aberrant AS can generate abnormal isoforms that affect the development and prognosis of cancer (9). For example, a specific AS event of PKM pre-mRNA produces PKM2, which contributes to a poor prognosis in multiple myeloma by promoting aerobic glycolysis (10). Abnormal AS of CD44 pre-mRNA produces the oncogenic isoform CD44v6, which promotes colorectal cancer (CRC) metastasis, and the overexpression of the CD44v6 isoform predicts poor overall survival (OS) in CRC patients (11). Given the influence of AS events (ASEs) in cancer, the use of gene-specific AS isoforms as prognostic predictors and therapeutic targets for cancer is promising.

Recently, studies have suggested that complex and dynamic relationships between cancer progression and immune genes exist at all clinical stages (12, 13). The use of changes in immune gene AS as promising diagnostic and therapeutic targets in cancer has attracted attention. For example, variable 5’-UTR splicing at exon 1 of the immune gene HLA-C produces different HLA-C isoforms that affect natural killer (NK) cell function, and different isoforms likely act as biomarkers that reflect NK cell activity in multiple cancers (14, 15). Moreover, in CRC, exon skipping in the 5’ coding region of the immune gene PD-L1 can generate splicing isoform b, which has a more significant inhibitory impact on T cells than the typical PD-L1 protein and may be a new biomarker for the efficacy of anti-PD-1/PD-L1 immunotherapy (16). However, current studies have mainly focused on the importance of individual ASEs of immune genes in only a few cases; a comprehensive overview of ASEs based on a large-scale cohort in cancer is still lacking.

In this study, we systematically profiled the immune-related ASEs of colon cancer patients and then constructed comprehensive and respective prognostic models based on 7 types of immune-related ASEs. We investigated the potential value of a comprehensive risk model in predicting prognosis, evaluating the immune microenvironment and guiding clinical treatment. Finally, we validated the immune-related function of significant ASEs in colon cancer cell lines and clinical samples. Our study provides novel insights into AS, cancer processes and the immune microenvironment.

## Materials and Methods

### Acquisition and Processing of Data

First, the RNA-seq data, single nucleotide polymorphisms (SNPs) data, copy number variations (CNVs) data and clinical information of colonic adenocarcinoma (COAD) patients were downloaded from the TCGA database (https://portal.gdc.cancer.gov/). A total of 473 COAD cases and 41 normal cases were obtained, and cases with an OS of at least 90 days were retained. Second, ASEs with percent spliced in (PSI) values for COAD were extracted from the TCGA SpliceSeq (17) website (https://bioinformatics.mdanderson.org/TCGASpliceSeq/). The PSI value is an objective ratio from 0 to 1 for quantifying an ASE. We stringently filtered PSI values for all ASEs (samples with PSI values ≥75% and average PSI values of samples ≥0.05).

**Figure 1:**
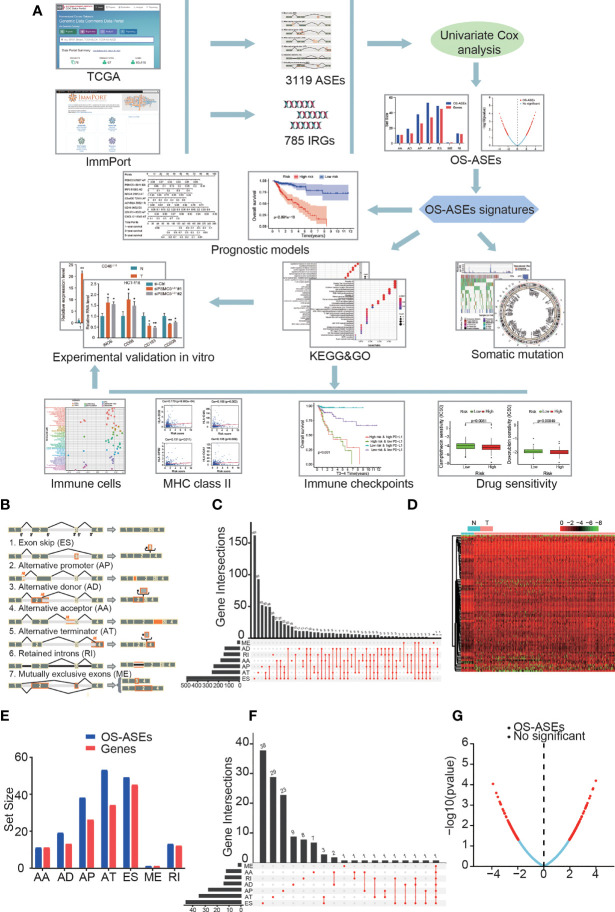
ASEs and OS-ASEs of IRGs. **(A)** Study flowchart. A total of 3119 ASEs based on 785 IRGs were obtained from TCGA and ImmPort databases. Then, 183 OS-ASEs was identified according to univariate COX analysis. Furthermore, final key OS-ASEs was selected to construct prognostic models. Subsequently, KEGG, GO analyses, immune-related analyses, somatic mutation, and drug sensitivity assays were applied to identify the potential function of OS-ASEs signature. Finally, *in vitro* validations were conducted to explore the expression and function of these OS-ASEs. **(B)** Schematic representation of seven different alternative splicing types: alternate acceptor site (AA); alternate donor site (AD); alternate promoter (AP); alternate terminator (AT); exon skip (ES); mutually exclusive exon (ME); and retained intron (RI). **(C)** UpSet plot of ASEs of IRGs. **(D)** Heatmap of differentially expressed ASEs based on IRGs between tumour and normal samples that showed a significant difference. **(E)** OS-ASEs of IRGs and corresponding genes identified through univariate Cox analysis. **(F)** UpSet plot of OS-ASEs of IRGs showing interactions of the seven types in colon cancer. **(G)** Volcano plot of AS events, in which the red dots and blue dots represent ASEs of IRGs that were correlated and not correlated with OS, respectively.

We next screened for ASEs involving immune-related genes (IRGs) with appropriate PSI values *via* the ImmPort data portal (https://www.immport.org/shared/home). The filtered ASEs were divided into the following 7 specific types (**Figure 1B**): alternate acceptor site (AA); alternate donor site (AD); alternate promoter (AP); alternate terminator (AT); exon skip (ES); mutually exclusive exon (ME); and retained intron (RI). The differential expression of the 7 AS sets between normal and tumour samples was visualized in a heatmap (log FC > 0.5 and false discovery rate (FDR) < 0.05) and presented in an UpSet plot for quantitative analysis. The procedures were performed using the limma, pheatmap and UpSetR packages of R language (version 4.0.4).

A matrix with 7 types of IRG-related ASEs was built, in which PSI values were listed with survival information by combining the AS and clinical data.

### The Identification of OS-ASEs and Evaluation of Their Characteristics

The consolidated AS matrix was generated by univariate Cox regression analysis to determine OS-related alternative splicing events (OS-ASEs). We generated an UpSet plot to display the OS-ASEs and a volcano plot to illustrate all of the ASEs. Bubble plots were created to show the top 20 OS-ASEs of the 7 ASE types, in which the colour and size indicate the correlative value for survival. Gene ontology (GO) and Kyoto Encyclopedia of Genes and Genomes (KEGG) analyses were applied to evaluate the functional categories with a meaningful p value (<0.05). The R packages used in these steps were the UpSetR, ggplot2, colorspace, stringi, DOSE, clusterProfiler and enrichplot packages.

We obtained a splicing factor (SF) list from the SpliceAid2 database (www.introni.it/spliceaid.html) and adopted Pearson correlation analysis to explore the interaction of the SFs with OS-ASEs. An SF-AS regulatory network was generated by Cytoscape (3.8.2), in which the regulation pairs with p< 0.05 and a correlation coefficient > 0.1 were included. We depicted SF and OS-ASEs as triangles and ellipses and high-risk and low-risk OS-ASEs as red and green ellipses, respectively, and promoting and inhibiting regulations are depicted by red and green lines, respectively.

### Construction and Validation of Prognostic Models

Significant OS-ASEs were screened by Lasso regression. The log lambda value, which we selected according to the minimum cross-validation error point, was calculated. The key OS-ASEs with nonzero coefficients corresponding to the selected log lambda value were obtained.

Then, through multivariate Cox regression, the key ASEs were further filtered to generate final OS-ASEs to include in the prognostic signatures with β values. The risk scores of the prognostic signatures for COAD outcome prediction were calculated by the following formula: $\sum_{i=1}^{n}\beta \text{i}\times \text{PSI}$ . The 1-, 3-, and 5-year receiver operator characteristic (ROC) curves were plotted to evaluate the accuracy of the prognostic model. Using the Akaike information criterion (AIC) values, the highest point of the 3-year curve calculated, which was closest point to the upper-left corner and the maximum inflection point, was used as the cut-off point to distinguish the high- and low-risk groups. Kaplan-Meier curves of survival and disease-free survival (DFS) were constructed to illustrate differences between the two groups. Moreover, according to the OS-ASEs of the prognostic model, nomograms were generated and used to predict the OS and DFS of patients. Calibration curves were generated to graphically assess the accuracy of the nomograms. The R packages utilized in these operations included the survival, glmnet, survivalROC, survminer, rmda and rms packages.

Univariate and multivariate Cox regression analyses were performed to evaluate whether the risk score could act independently along with age, sex, clinical stage, and TNM stage. In addition, the results were visualized in forest maps. Chi-square and Wilcoxon signed-rank tests were used to judge the correlations between the risk score and clinicopathological characteristics. A band diagram was employed for visualization, in which p<0.001, p<0.01, and p<0.05 were labelled by ***, **, and *, respectively. A heatmap showing the expression levels of 9 OS-ASEs in the risk groups was generated. The R packages utilized in these operations included the survival, forestplot and pheatmap packages.

### Identification of Significantly Mutated Genes

The nucleotide mutational data were saved in the TCGA mutation annotation format (maf), and the maf data were processed and analyzed using Chi-square test to select significantly nucleotide mutational genes (P < 0.05). We then utilized R package maftools to visualize the SNP distribution and frequency in the low/high risk samples among patients.

### Integrative Analysis of Significant Copy Number Variation

Reference genome was Genome Research Consortium Human build 38 (GRCh38). This study calculated the ratios of copy number variation (CNV) of the genes both in normal and low/high risk tumour samples. Then, the gene-CNV matrix was constructed after using Chi-square test. CNVs change rates between normal and low/high risk samples were further compared through Chi-square test, and CNVs data including significant CNV genes (P < 0.05) were identified. Circos plot was used to show the significant CNV in the genome by means of the R packages RCircos.

### Evaluation of Tumour-Infiltrating Immune Cells and HLA Genes

We visualized the proportion of each immune cell and their correlation in the COAD patient cohort through CIBERSORT and the Wilcoxon rank sum test. Then, we adopted seven methods, TIMER, CIBERSORT, XCELL, QUANTISEQ, MCP-counter, EPIC, and CIBERSORT-ABS, to evaluate the immune infiltration status among the patients from the TCGA LIHC dataset. The relationships between the risk score and infiltrating immune cells were determined by Spearman correlation analysis. A lollipop diagram was generated to display the correlation coefficients of the results with p <0.05. In addition, several linear correlation plots were generated to illustrate the links between HLA genes and the risk score *via* Spearman correlation analysis. The R packages involved in the analyses were corrplot, scales, limma, ggplot2 and ggtext.

### Investigation of the Risk Model in the Clinic

Linear correlation plots were used to show the relationships between the risk score and the expression levels of immune checkpoint genes, including PD-L1, CTLA-4, LAG-3 and LAIR-1. Kaplan-Meier survival curves were generated to assess the survival status of four patient groups stratified by high/low expression of immune checkpoint-related genes and high/low risk score. In addition, we determined the half inhibitory concentration (IC50) of common chemotherapies and potential targeted drugs, such as doxorubicin, camptothecin, vinblastine and gemcitabine, in the TCGA LIHC dataset. We displayed the differences in drug IC50 between the two risk groups in the form of box drawings generated *via* the Wilcoxon signed-rank test. The R packages utilized in this procedure were survival, survminer, car, ridge, preprocessCore, genefilter, sva and ggpubr.

### ES-Related Downstream Protein-Protein Interactions

ES-related protein-protein interaction (PPI) and domain–domain interaction (DDI) networks were available on the DIGGER (18) website (https://github.com/louadi/DIGGER). A joint PPI and DDI network graph was produced, in which the nodes represented protein domains or proteins and the edges between the nodes represented DDIs or PPIs.

### Clinical Sample Collection and CRC Cell Line Culture

All clinical samples were collected from the Gastrointestinal Surgery Department of Affiliated Hospital of Xuzhou Medical University, and sample collected was approved by the Medical Ethics Committee of the hospital. All the clinical samples were stored at -80°C. All cell lines were purchased from ATCC, and the cells were cultured in McCoy’s 5A or RPMI 1640 medium (Gibco BRL, United States) in an incubator at 37°C, 95% humidity and 5% CO_2_.

### RNA Extraction and qRT–PCR

Total RNA was extracted from cells and tissues using the TRIzol method (Dongsheng Bio. #R1022) following the protocol. Then, the obtained RNAs were processed for cDNA synthesis. qRT–PCR was then performed using SYBR Green qPCR Mix (Dongsheng Bio. #P2092) and analysed on a Roche LightCycler system. The expression levels of the target genes were normalized based on the expression level of GAPDH. The primer sequences used for amplification and siRNA sequences are listed in the **Supplemental Material**.

### Cell Counting Kit-8 Assay

The transfected cells were seeded at 5 × 10^3^ cells per well in 96-well plates. After 24, 48 and 72 hours, 10 µl CCK-8 reagent (Dojindo, Japan) and 100 µl medium were added to each well, and the cells were then incubated in a cell incubator at 37°C for 2 h. By measuring the optical density (OD) value at 450 nm, the cell growth rate was calculated.

### T Cell-Mediated Tumour Cell Killing Assay

Primary human T cells were activated using CD3 antibody (Abcam, UK) and CD28 antibody (Abcam, UK). Colon cancer cells and activated T cells were cocultured in 6-well plates, and the wells were then washed with PBS three times to remove T cells after a period of coculture. The surviving cancer cells were fixed and stained using crystal violet solution.

### Macrophage Polarization Experiments

The Transwell method was used to detect the effect of colon cancer cells on the polarization of macrophages. Transfected colon cancer cell lines (HCT-116 and SW480) were added to the upper chamber, and THP-1 cells were seeded in the lower chamber. Before adding colon cancer cells, THP-1 cells were treated with 100 ng/ml PMA (Sigma, USA) for 1 day to induce M0 macrophages. After coculture for 48 h, the mRNA levels of M1 and M2 macrophage markers were detected by qRT–PCR analysis.

## Results

### Identification of IRG-Related ASEs and OS-ASEs

The research flow chart of this study is shown in **Figure 1A**. The data for 437 COAD samples were obteined from the TCGA database and 380 samples were included after data arrangement. We listed a summary of the included COAD sample characteristics in **Table 1** and detailed clinical information in **Table S1**. The PSI value of sample, which represents the inclusion of a transcript portion divided by the overall reads, was used to quantify each ASE. We screened ASEs of IRGs with PSI values across the genome in COAD and detected 3,119 ASEs of 785 IRGs (**Figure 1C**) (**Additional File 1**). When defining the FDR (FDR<0.05) and logFC (>0.5) cut-offs, we generated a heatmap (**Figure 1D**) to illustrate the significant differences in ASEs of IRGs between normal and cancer samples, suggesting that these ASEs play an important role in the colon cancer process.

**Table 1:** The clinical characteristics of COAD patients.

Characteristics	Total samples	Risk groups		t –test
		Low n = 235	High n = 145	P value
**Age (years)**				0.854
≤65 years	157	99	58	
>65 years	223	136	77	
**Gender**				0.835
Female	173	106	67	
Male	207	129	78	
**Pathological stage**				
Stage I Stage II Stage III Stage IV Unknown	66 144 109 50 11	46 95 67 20 7	20 49 42 30 4	0.002**
**AJCC-T stage**				<0.001***
T0/Tis T1 T2 T3 T4	1 9 67 258 45	1 7 47 163 17	0 2 20 95 28	
**AJCC-N stage**				0.002**
N0	223	149	74	
N1 N2	91 66	57 29	34 37	
**AJCC-M stage**				<0.001***
M0 M1 Unknown	284 50 46	184 20 31	100 30 15	
**Survival status**				<0.001***
Alive	309	215	94	
Dead	71	20	51	

*P < 0.05, **P< 0.01, ***P< 0.001.

Through univariate Cox proportional hazards regression analysis, we investigated the associations between PSI values of ASEs and the OS time of patients and determined the OS-ASEs (p<0.05). The distribution of all ASEs showed a remarkably normal distribution in the volcano plot (**Figure 1G**). 11 AA events in 11 genes, 19 AD events in 13 genes, 38 AP events in 26 genes, 53 AT events in 34 genes, 49 ES events in 45 genes, 1 ME event in 1 gene, and 13 RI events in 12 genes were identified as OS-ASEs (**Figure 1E**) (**Additional File 2**). From these results, it can be seen that one IRG can have more than one OS-ASE. Furthermore, IRG expression for the 7 AS types was visualized with an UpSet plot (**Figure 1F**).

### Molecular Characteristics and SF-AS Network of OS-ASEs

**Figure 2:**
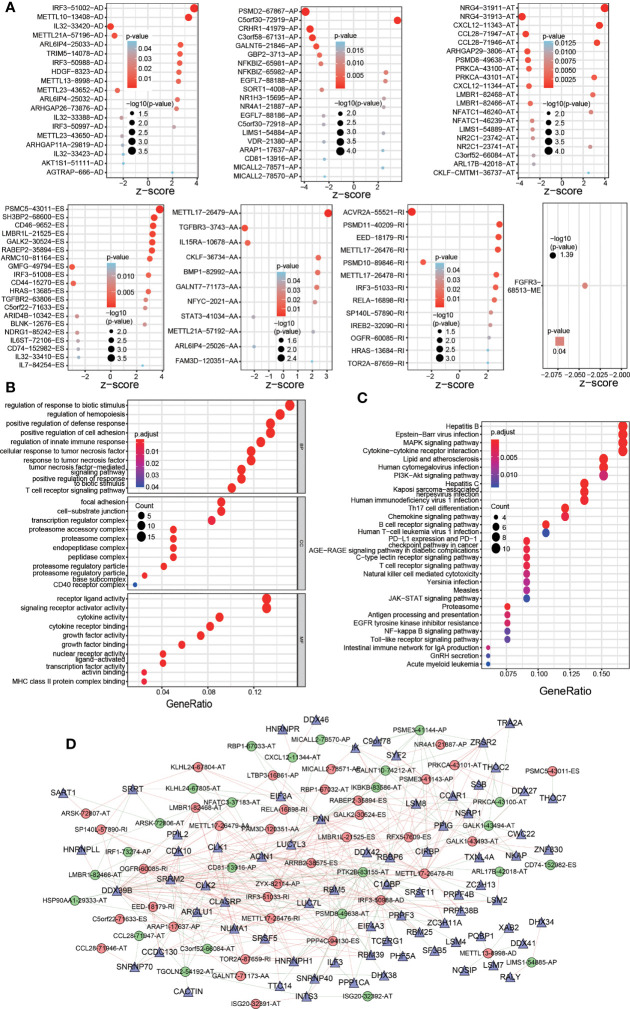
Significant OS-ASEs in colon cancer and their characteristics. **(A)** Bubble plots of the top 20 most significant OS-ASEs correlated with clinical outcome based on type (AA, AD, AP, AT, ES, ME and RI). GO **(B)** and KEGG pathway **(C)** analyses of OS-ASEs. **(D)** The SF-AS regulatory network. In the network, high-risk and low-risk OS-ASEs are represented by red and green circles, respectively, and SFs are represented by triangles. The red and green lines indicate positive and negative regulation, respectively.

The distribution of significant OS-ASEs in COAD is shown in bubble plots, including AAs, ADs, ATs, APs, ESs, ME, and RIs (**Figure 2A**). Next, we explored the molecular characteristics of IRGs with OS-ASEs by GO (**Figure 2B**) and KEGG analyses (**Figure 2C**). According to the GO analysis results, the significant biological processes (BPs) were regulation of response to biotic stimulus, regulation of haemopoiesis, and positive regulation of defence response, etc. The cellular component (CC) enrichment results showed that the most significantly enriched CC terms were focal adhesion, cell-substrate junction, transcription regulator complex, etc. The significant molecular functions (MFs) included receptor ligand activity, signalling receptor activator activity, cytokine receptor binding, etc. In addition, KEGG analysis identified the enriched pathways of the meaningful IRGs, including pathways related to hepatitis B, Epstein-Barr virus infection, MAPK signalling pathway, etc.

Because ASEs are regulated by SFs, which bind directly to pre-mRNAs, we generated an SF-AS regulatory network with the purpose of exploring the correlation between OS-ASEs and SFs (**Figure 2D**). In the network, 12189 postive and 11814 negative regulations between SFs and all the OS-ASEs were identified (p< 0.05, cor>0.1). The detailed correlation information of the SF-AS network is listed in **Additional File 3**.

### Establishment and Assessment of Comprehensive and Specific Prognostic Models Based on OS-ASEs

**Figure 3:**
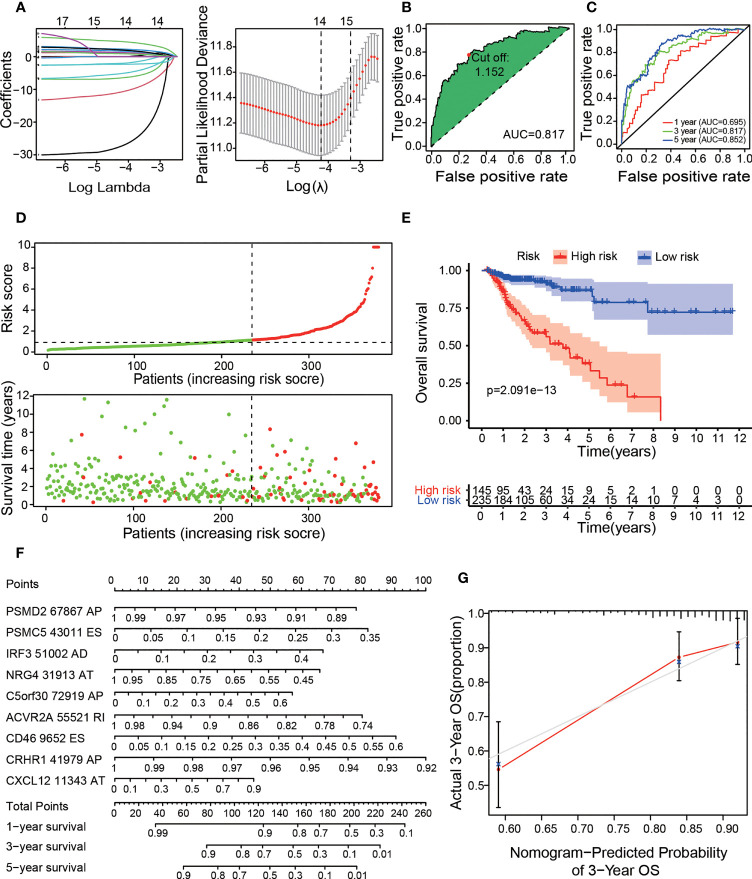
Construction of the prognostic risk model with OS-ASEs. **(A)** LASSO Cox analysis. The right subfigure shows the process by which the log lambda value, which we selected as the minimum cross-validation error point, was calculated. The OS-ASEs with nonzero coefficients corresponding to the selected log lambda value were obtained for risk model construction in the left subfigure. **(B)** The ROC curve with the maximum inflection point for the optimal prognostic model. **(C)** The 1-, 3-, and 5-year ROC plots for the prognostic model. **(D)** The risk plot between the high- and low-risk groups. **(E)** Kaplan–Meier curve of OS outcomes for patients. The lighter blue and red regions represent the 95% CI areas of the prognosis curve. **(F)** Nomogram for predicting OS probabilities. The points achieved for each risk factor were summed on the total point axis, and the total points correspond to the OS probability of patients. **(G)** Calibration curve for evaluating the accuracy of the OS nomogram with 3-year OS data from the TCGA cohort.

Based on the OS-ASEs, we constructed a comprehensive prognostic model for COAD patients. First, to prevent overfitting of the model, Lasso regression was used to screen all 183 OS-ASEs. As shown in **Figure 3A**, the log-lambda value with the smallest model cross-validation error was between -4 and -5, and the corresponding OS-ASE number was 14. Next, through forward and backward screening of the Cox model, we ultimately obtained 9 OS-ASEs: PSMD2-67867-AP; PSMC5-43011-ES; IRF3-51002-AD; NRG4-31913-AT; C5orf30-72919-AP; CRHR1-41979-AP; CXCL12-11343-AT; ACVR2A-55521-RI; and CD46-9652-ES. We calculated the risk score of each COAD patient based on the 9 OS-ASEs, and the details are shown in **Additional File 4**. Then, we determined the areas under the curve (AUC) values for the ROC curves of 9 OS-ASEs, and the 1-, 3-, and 5-year AUC values were 0.695, 0.817 and 0.852, respectively, with appropriate sensitivities and specificities (**Figure 3C**). With the aim of distinguishing the high- and low-risk COAD groups, we adopted the maximum inflection point as the optimal cut-off point on the 3-year ROC curve by AIC values (**Figure 3B**). Accordingly, 235 cases with risk scores lower than 1.152 were classified into the low-risk group, and 145 cases with risk scores greater than or equal to 1.152 were classified into the high-risk group (**Figure 3D**). In Kaplan-Meier analysis, the prognostic model suggested that the patients in the low-risk group had better OS outcomes than those in the high-risk group (p<0.001) (**Figure 3E**). To further improve the predictability of the prognostic model, we established a risk nomogram with an applicable C index value (0.73) for predicting the OS probability of COAD patients. As depicted in **Figure 3F**, a higher total point based on the sum of each OS-ASE point corresponded to worse 1-year, 3-year and 5-year survival rates. To demonstrate the accuracy of the OS nomogram, we generated a calibration curve and found good agreement between the predicted and actual 3-year OS values (**Figure 3G**). Moreover, we further developed 6 prognostic models based on six types of AS (AA, AD, AP, AT, ES and RI) (**Figures S1**, **2**). All 6 prognostic models were confirmed to be meaningful.

**Figure 4:**
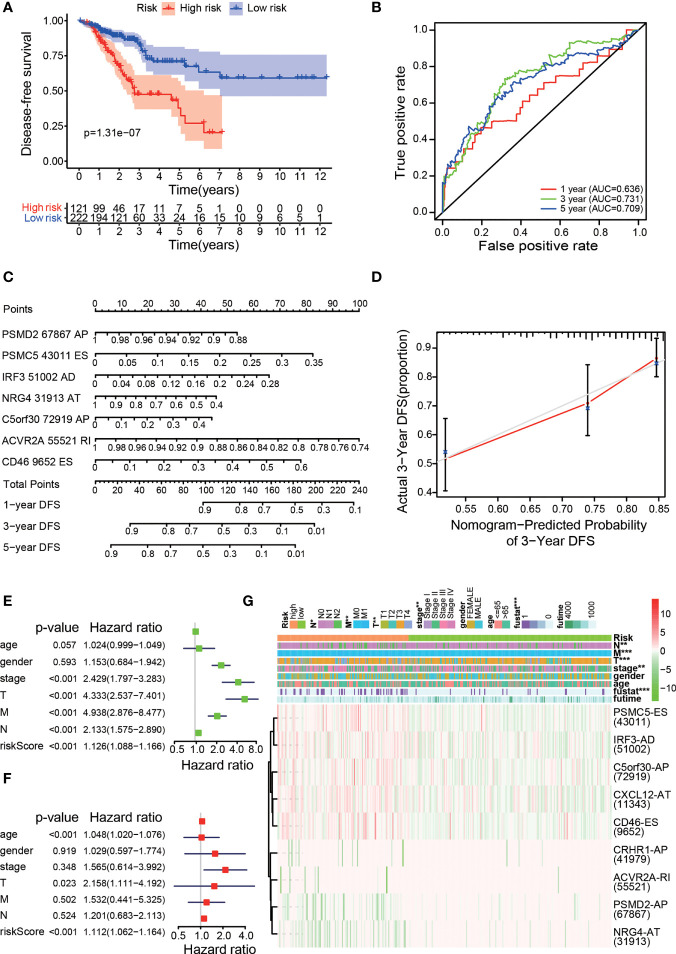
Clinical evaluation of the prognostic risk model. **(A)** Kaplan–Meier curve for DFS prediction of colon cancer patients. **(B)** The 1-, 3-, and 5-year ROC plots of the prognostic model for predicting DFS outcomes. **(C)** Nomogram for predicting DFS probabilities. **(D)** Calibration curve for evaluating the accuracy of the DFS nomogram using 3-year DFS data. Univariate Cox regression **(E)** and multivariate Cox regression **(F)** analyses of clinical parameters and the risk score, in which the P values of the risk score were both less than 0.001, indicated that the risk score can act as an independent prognostic factor in colon cancer. **(G)** A strip chart along with a scatter diagram showing that T stage, N stage, M stage, clinical stage, and survival status were significantly related to the risk score. The bottom heatmap displays the scatter of each OS-ASE (the green and red colours correspond to low and high PSI values, respectively). *p < 0.05, **p < 0.01, and ***p < 0.001.

To evaluate whether the high-/low-risk group in the prognostic model was related to cancer recurrence, we performed Kaplan-Meier analysis to construct a prognostic model of DFS based on the risk groups used with the comprehensive prognostic model (**Figure 4A**), and our results indicated that patients in the low-risk group had lower recurrence rates than those in the high-risk group (p<0.001), with acceptable accuracy (**Figure 4B**). Then, we obtained 7 meaningful ASEs from the 9 OS-ASEs through Lasso regression and further constructed a risk nomogram for predicting DFS (**Figure 4C**). The calibration curve (**Figure 4D**) showed excellent accuracy of the DFS nomogram based on actual 3-year DFS data in the TCGA cohort. To validate whether the risk score derived from the comprehensive prognostic model can act as an independent predictor, we performed univariate and multivariate Cox analyses of the risk score along with age, sex, clinical stage and TNM stage (**Figures 4E, F**). The risk score was independently associated with prognosis (p<0.001). Next, we produced a heatmap to describe the relationships between the risk score derived from the comprehensive model, the PSI values of OS-ASEs and clinical characteristics (**Figure 4G**). The top half of the heatmap depicts the clinical correlations in the two groups and shows that the risk score was correlated with N stage (p<0.01), M stage (p<0.05), T stage (p<0.01), clinical stage (p<0.05) and survival status (p<0.001). The bottom half of the heatmap shows the distribution of the PSI values of the 9 OS-ASEs in the low- and high-risk groups.

### Cancer-Related Somatic Mutation in the Low and High Immune-Related Risk Groups

**Figure 5:**
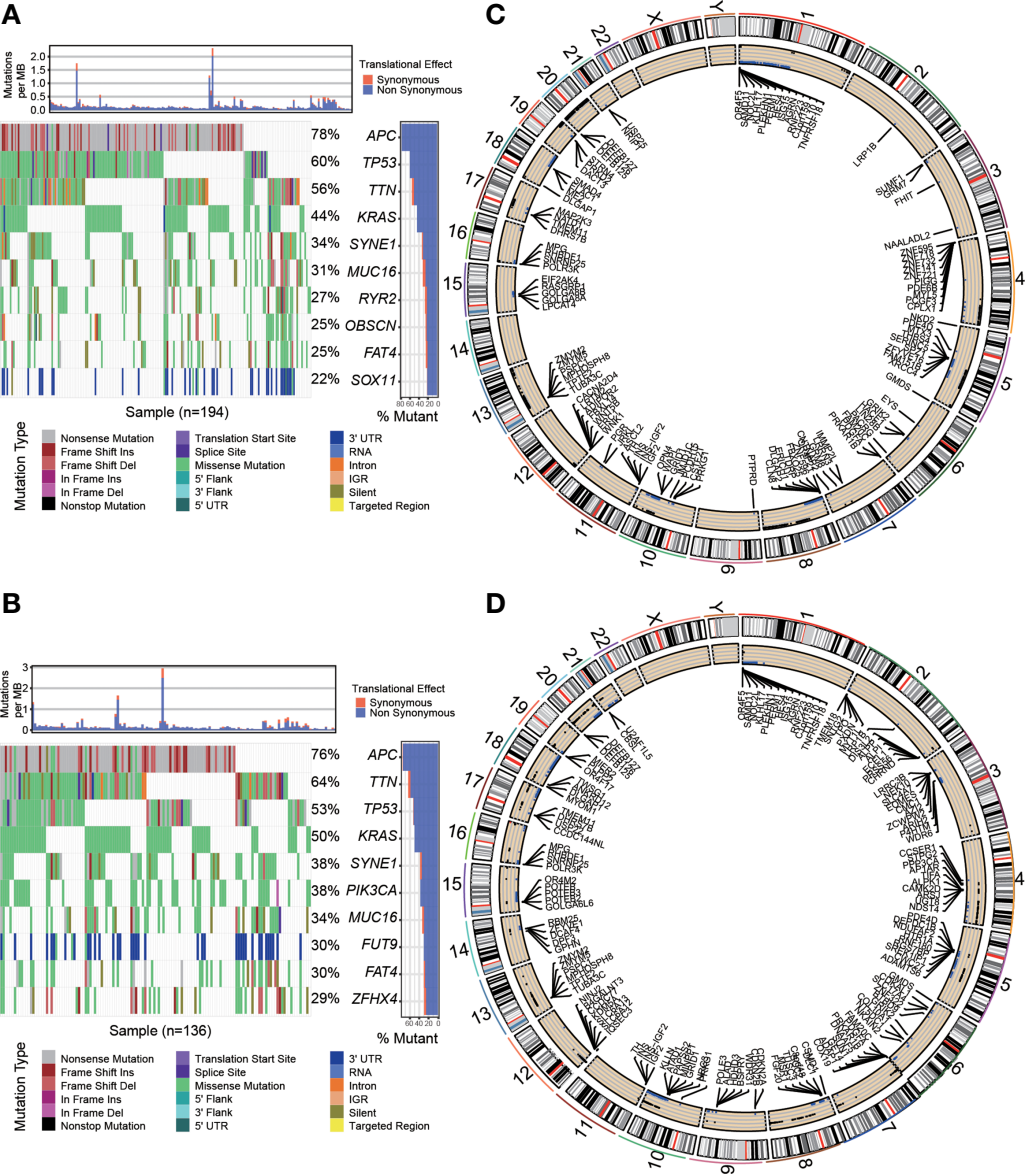
Cancer-related somatic mutation in the two risk groups. Waterfall plots showing the SNP information in the low-risk group **(A)** and high-risk group **(B)**. The location of CNVs in the low-risk group **(C)** and high-risk group **(D)** visualized by Circos plots. The outside circle shows chromosomes; the inside circle illustrates the distribution of CNVs (black or blue points represent the copy number gain or loss, respectively).

To explore the difference in cancer-related somatic mutation between the high- and low-risk groups, we investigated single nucleotide polymorphisms (SNPs) and copy number variations (CNVs) in both groups. First, we calculated the frequency and distribution of SNPs. The waterfall plots illustrate the representative SNPs in each group (**Figures 5A, B**). APC (78%), TP53 (60%), TTN (56%), KRAS (44%) and SYNE1 (34%) were the top five genes with nucleotide mutations in the low-risk group. APC (76%), TTN (64%), TP53 (53%), KRAS (50%) and SYNE1 (38%) were the top five genes with single nucleotide mutations in the high-risk group. Many genes such as TTN had a relatively higher mutation rate in the high-risk group (64% vs. 56%), while some genes such as TP53 presented a relatively lower mutation rate in the high-risk group (53% vs. 60%). We further analyzed CNV in the two risk groups. The Circos plots show the chromosomal location of the significant genes and copy number gain or loss is more common in high-risk samples than in low-risk samples (**Figures 5C, D**). Detailed information about the genes with CNV in the two groups is provided in **Additional File 5**.

In summary, risk groups can distinguish differences in somatic mutations, suggesting that there are important links between OS-ASEs and somatic mutations.

### Estimating Tumour Infiltrating Immune Cells and Immume Response With the Risk Assessment Signature

**Figure 6:**
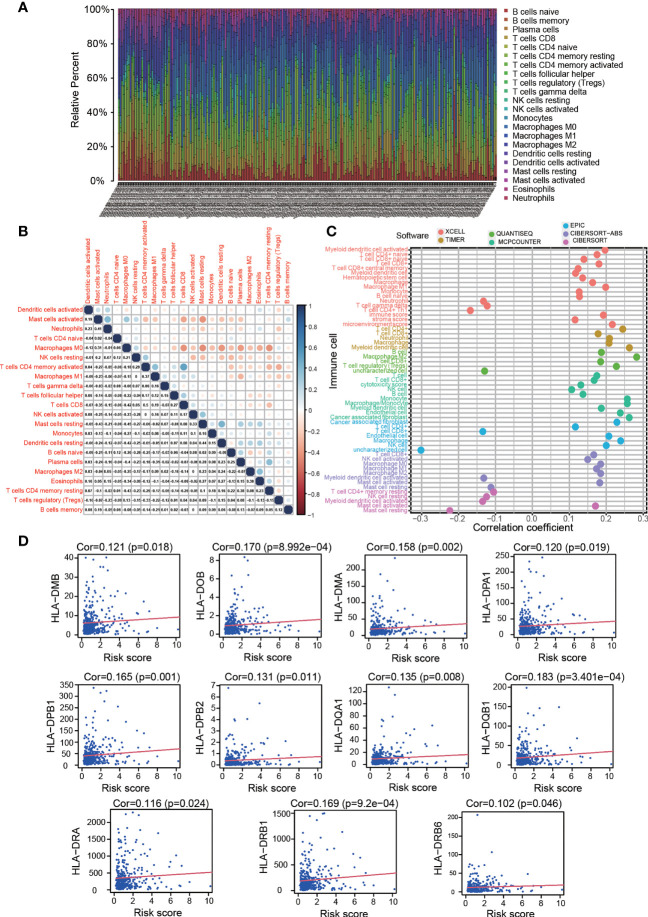
Estimation of tumour-infiltrating cell proportion and HLA-D region gene expression by risk model. **(A)** Bar plot of the proportion of each immune cell type in the colon cancer patient cohort. **(B)** Correlations between the immune cell proportions in colon cancer. **(C)** Correlations between tumour-infiltrating immune cell proportions and risk score. High risk scores were more positively related to most tumour-infiltrating immune cells, such as macrophages, CD4+ T cells and CD8+ T cells, as shown by Spearman correlation analysis. **(D)** High risk scores were positively correlated with HLA-D region gene expression.

Because OS-ASEs were initially identified based on their association with IRGs, we attempted to identify relationships between the risk model and the immune microenvironment of COAD. First, using the CIBERSORT method and the Wilcoxon rank sum test, we determined the proportion of each immune cell type in the COAD patient cohort (**Figure 6A**), and some relationships between immune cells were strong, such as the relationships between resting memory CD4+ T cells and M0 macrophages (r=-0.43), CD8+ T cells and resting memory CD4+ T cells (r=-0.26), and CD8+ T cells and M0 macrophages (r=-0.42) (**Figure 6B**). Then, we adopted the abovementioned seven methods to calculate the correlation coefficients between tumour-infiltrating immune cells and the risk score. As shown in **Figure 6C**, the risk score had positive relations with most tumour-infiltrating immune cells, such as M2 macrophages and CD4+ T cells (**Additional File 6**).

The foregoing GO analysis results showed that the expressed genes related to OS-ASEs were significantly related to MHC class II protein complex binding, receptor ligand activity and signalling receptor activator activity. Recent studies have also reported that MHC molecules can present peptides to the immune system and induce subsequent T cell responses (19). We investigated the expression of both the MHC I and II family genes and discovered that HLA-D region genes (MHC class II), including HLA-DMA, HLA-DMB, HLA-DOB, HLA-DPA1, HLA-DPB1, HLA-DPB2, HLA-DQA1, HLA-DQB1, HLA-DRA, HLA-DRB1 and HLA-DRB6, were positively related to the risk score (p<0.05) (**Figure 6D**). These results suggested that OS-ASEs could affect T cell responses by regulating HLA-D region gene expression.

**Figure 7:**
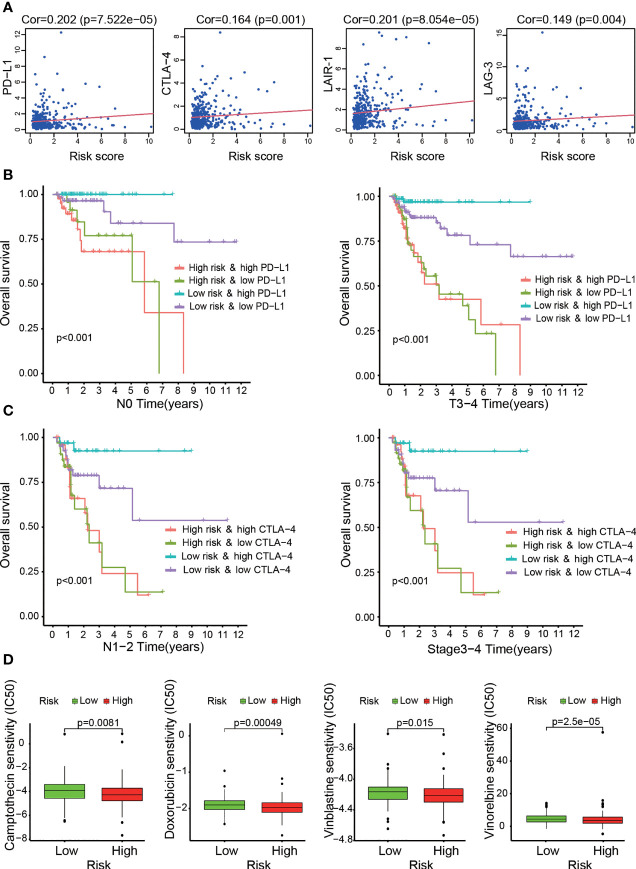
Assessment of immune checkpoint gene expression and its impact on clinical outcome by the risk model. **(A)** High risk scores were positively correlated with immune checkpoint gene (CTLA-4, LAG-3, LAIR-1 and PD-L1) expression. **(B, C)** Kaplan–Meier curves for OS among the four groups. Patients stratified by immune checkpoint gene (PD-L1 or CTLA-4) expression and risk score potentially had different outcomes. **(D)** The risk model acted as a predictor for chemosensitivity, as patients with high risk scores tended to have lower IC50 values for chemotherapy drugs, such as doxorubicin, camptothecin, vinblastine and gemcitabine.

The KEGG results indicated that the genes related to OS-ASEs were enriched in PD-L1 expression and the PD-1 checkpoint pathway in cancer. As classical immunosuppressive molecules, PD-L1 and PD-1 can regulate the activation of T cell. Our study found that the risk score was positively correlated with high expression of PD-L1 (p<0.001, **Figure 7A**). We further determined whether the risk score was associated with other biomarkers for immune checkpoints. Correlation analysis supported that the risk score was positively related to the expression of CTLA-4 (p=0.001), LAG-3 (p<0.01), and LAIR-1 (p< 0.001, **Figure 7A**). Immune checkpoint inhibitors (ICIs) have been applied for treating colon cancer in clinical practice, but selecting the right application is still unclear at present (20). Furthermore, we compared the clinical outcomes of four patient groups (a high-risk and low-risk group and a high-ICI and low-ICI gene expression group). As shown in **Figure 7B**, patients with T3-T4 stage or N0 stage disease, low risk and high PD-L1 expression had better OS outcomes than those with low risk and low PD-L1 expression. Similarly, in the low-risk group, patients with N1-N2 or stage 3–4 disease and high CTLA-4 expression experienced outcome benefits (**Figure 7C**). Interestingly, these results suggest that in patients with specific stage disease, immune checkpoint genes have protective effects, and the results of ICI treatment were the opposite of those expected.

Altogether, relationships between the risk score and immune infiltration cells and related immune response genes were evaluated. These results indicated that the risk level of patients was associated with the infiltration of immune cells and related immune responses, in which OS-ASEs play crucial roles.

### The Relationships Between the Risk Model and Chemotherapeutics

In addition to the previous analysis of immunotherapy-related relationships, we identified whether the model was associated with the efficacy of common chemotherapeutics used for treating COAD according to the TCGA LIHC dataset. We found that a high risk score was related to a lower IC50 of chemotherapeutic drugs such as doxorubicin (p<0.001), camptothecin (p<0.01), vinblastine (p<0.05) and vinorelbine (p<0.001) (**Figure 7D**). Additional analyses showed that the risk score was a potential predictor of chemosensitivity (**Additional File 7**).

### The Prognostic Value and Potential Influence of Independent OS-ASEs

**Figure 8:**
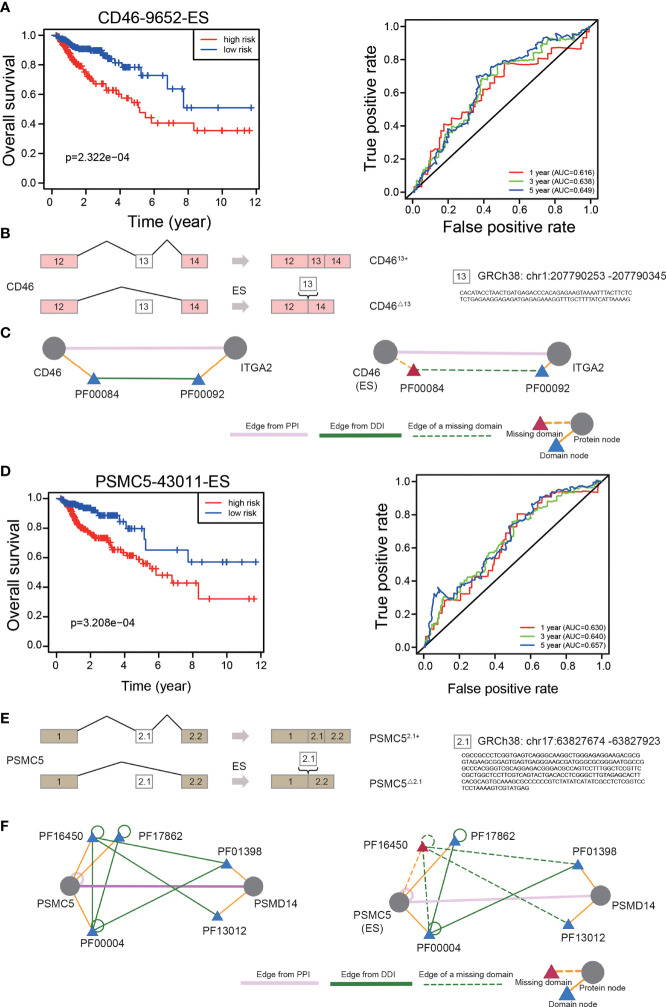
The prognostic value of independent OS-ASEs with downstream effects. **(A)** The Kaplan–Meier and 1-, 3-, and 5-year ROC curves of the OS prognostic model based on CD46-9652-ES. **(B)** The AS changes of CD46. **(C)** The change of interaction between CD46 and ITGA2 after different AS events of CD46. Triangles represent protein domains. The ES of CD46 can result in losing PF00084, which is the only domain that interacts with PF00092 of the ITGA2 protein. **(D)** The 1-, 3-, and 5-year ROC curves and Kaplan–Meier curves of the OS prognostic model based on PSMC5-43011-ES. **(E)** The AS changes of PSMC5. **(F)** Three domains of PSMC5 that mediate the interaction with PSMD14 and two of the three interactions are lost due to PSMC5 exon skipping.

To explore the prognostic significance of individual OS-ASEs, we selected ES events as examples. First, through ROC curve and Kaplan–Meier analyses, we illustrated the prognostic value of CD46-9652-ES in CRC (p<0.001) (**Figure 8A**). Patients with a high incidence of CD46-9652-ES had significantly poorer OS outcomes. Next, using the TCGA SpliceSeq database, we showed that CD46-9652-ES corresponds to CD46 exon 13 skipping and results in the increased expression of CD46^△13^ (exon 13 skipping) and decreased expression of CD46^13+^ (exon 13 inclusion) (**Figure 8B**). Changes in AS always have downstream effects, including PPI network alterations. Furthermore, by the DIGGER method, we revealed the PPI changes mediated by the alternative exon domain of CD46. As shown in **Figure 8C**, PF00084 domain of the CD46 protein mediated the interaction with ITGA2, and ES of CD46 could cause the PF00084 coding domain to be omitted, consequently preventing the interaction between CD46 and ITGA2.

We also identified that a high frequency of PSMC5-43011-ES increased mortality (p<0.001) (**Figure 8D**). This AS event led to an increase in the expression of PSMC5^△2.1^ (exon 2.1 skipping) and a decrease in the expression of PSMC5^2.1+^ (exon 2.1 inclusion) (**Figure 8E**). We further found that the PPI between PSMC5 and PSMD14 was controlled by 3 domains of the PSMC5 protein, and ES of PSMC5 disrupted 2 of the 3 interactions (**Figure 8F**).

### Validation of OS-ASEs Levels in Colon Cancer

**Figure 9:**
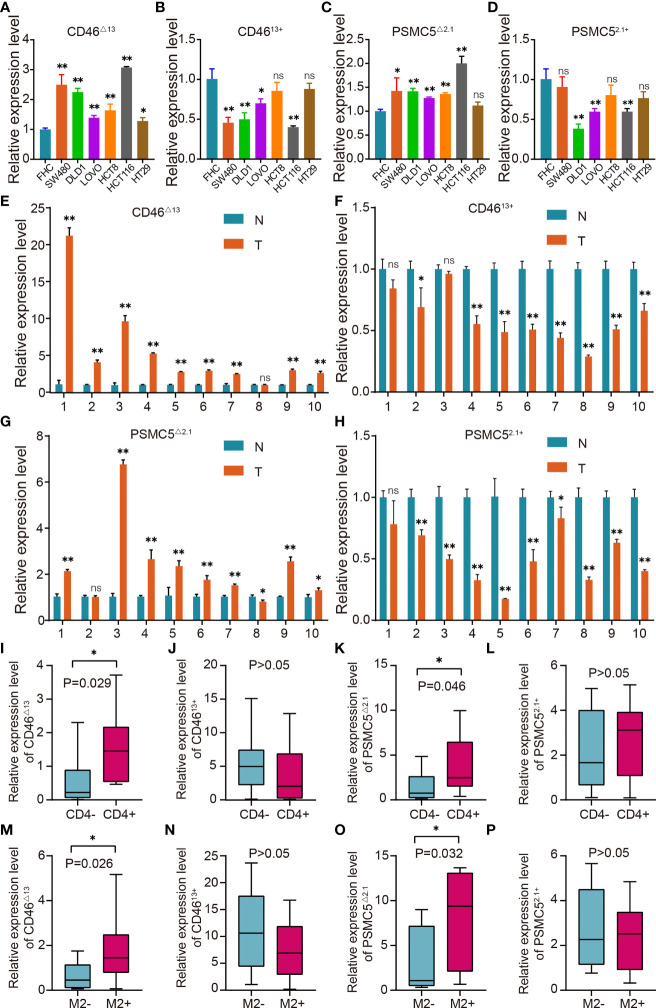
OS-ASE levels and the association between OS-ASEs and infiltration of immune cells in colon cancer. **(A–D)** The expression levels of CD46^△13^ and CD46^13+^, PSMC5^△2.1^ and PSMC5^2.1+^ in colon cancer cell lines (HCT116, HCT8, DLD1, LoVo, SW480, SW620) and FHC cell lines. **(E–H)** Expression analysis of CD46^△13^, CD46^13+^, PSMC5^△2.1^ and PSMC5^2.1+^ in 10 pairs of colon cancer tissue samples. **(I–L)** The expression of CD46^△13^, CD46^13+^, PSMC5^△2.1^ and PSMC5^2.1+^ in colon cancer samples characterized by CD4+/CD4- T cell infiltration. **(M–P)** The expression of CD46^△13^, CD46^13+^, PSMC5^△2.1^ and PSMC5^2.1+^ in colon cancer samples characterized by high/low infiltration of M2 macrophages. ^ns^p > 0.05, *p < 0.05 and **p < 0.01.

As significant AS events, CD46-9652-ES led to the increased expression of CD46^△13^ and the decreased expression of CD46^13+^. To validate the role of CD46-9652-ES, we first investigated the expression levels of CD46^△13^ and CD46^13+^ in colon cancer and normal colon cell lines. As shown in **Figures 9A, B**, we found that in colon cancer cells, the expression of CD46^△13^ was higher and the CD46^13+^ level was lower or indistinguishable compared to that of normal colon cells, suggesting that the ratio of CD46^△13^ vs. CD46^13+^ was increased in colon cancer cell lines. We further validated the expression levels of CD46^△13^ and CD46^13+^ in 10 paired samples from colon cancer patients. qRT–PCR results revealed that the expression and proportion of CD46^△13^ were higher in tumour tissues than in tumour-adjacent tissues (**Figures 9E, F**). In addition, PSMC5-43011-ES increased PSMC5^△2.1^ expression and decreased PSMC5^2.1+^ expression. We detected the expression of PSMC5^△2.1^ and PSMC5^2.1+^ in cell lines and clinical samples. Increased expression of PSMC5^△2.1^ and ratios of PSMC5^△2.1^ transcript vs. PSMC5^2.1+^ transcript were observed in colon cancer cells or tumour tissues compared to normal colon cell lines or tumour-adjacent tissues (**Figures 9C, D, G, H**). These data showed that the OS-ASEs CD46-9652-ES and PSMC5-43011-ES were increased in colon cancer and may be related to colon cancer progression.

### The Association Between OS-ASEs and Infiltration of Immune Cells

OS-ASEs have been associated with immune signatures and have been found to be positively correlated with multiple tumour-infiltrating immune cells, including CD4+ T cells and M2 macrophages (**Figure 6C**). We next investigated the CD46-9652-ES and PSMC5-43011-ES levels in 20 colon cancer samples characterized by CD4+/CD4- T cell infiltration and 20 colon cancer samples characterized by high/low M2 macrophage infiltration. The abundance of CD46^△13^ and PSMC5^△2.1^ transcripts was higher in tumour tissues with CD4+ T cell infiltration or high M2 macrophage infiltration than in tissues with CD4- T cell infiltration or low M2 macrophage infiltration, while CD46^13+^ and PSMC5^2.1+^ expression did not differ between tissues with high and low infiltration of immune cells (**Figures 9I–P**). Taken together, these results indicate that the levels of CD46-9652-ES and PSMC5-43011-ES may reflect the infiltration of immune cells, in accordance with the above bioinformatics research.

### The Role of OS-ASEs With Immune Signatures in Colon Cancer

**Figure 10:**
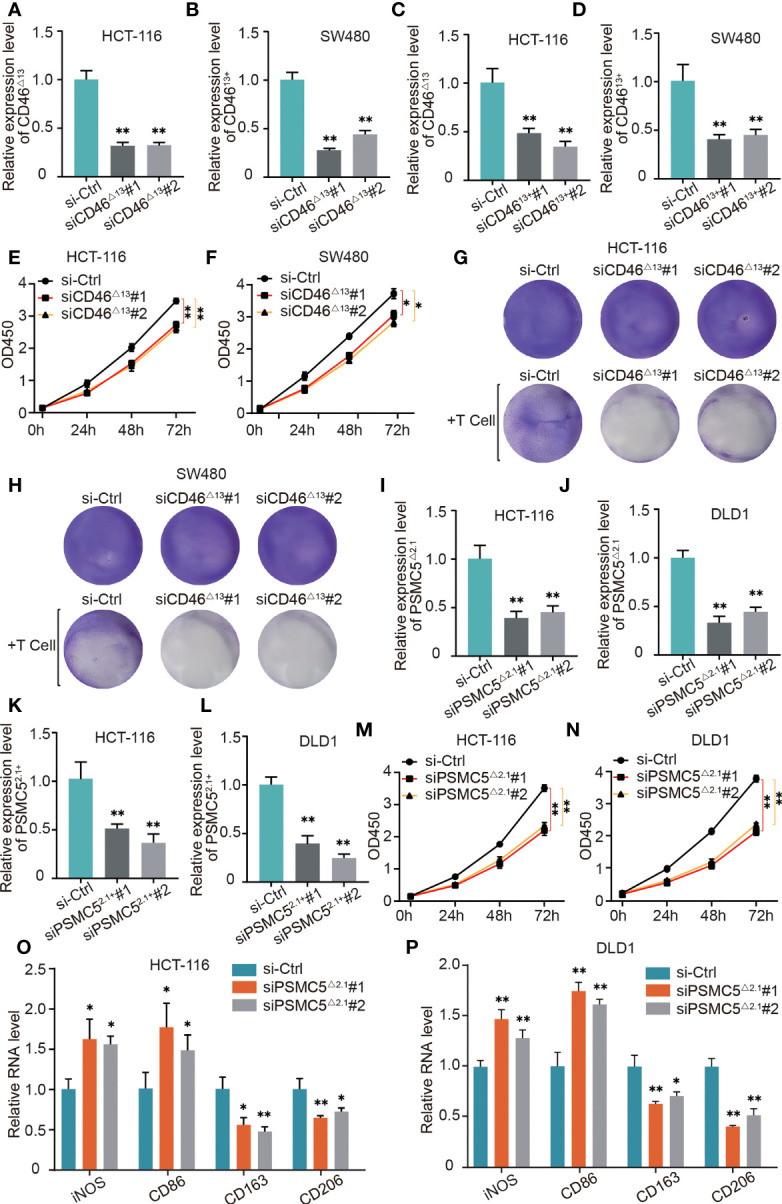
The role of OS-ASEs with immune signatures in colon cancer. **(A–D)** Relative expression levels of CD46^△13^ and CD46^13+^ after transfection with siRNA in HCT116 and SW480 cells. **(E, F)** The proliferation ability of HT116 and LoVo cells after silencing CD46^△13^ expression. **(G, H)** T cell-mediated tumour cell killing of HT116 and LoVo cells treated with si-CD46^△13^. Activated T cells and colon cancer cells were cocultured in 6-well plates for 4 days, and crystal violet staining showed the surviving tumour cells. **(I–L)** Relative expression levels of PSMC5^△2.1^ and PSMC5^2.1+^ after transfection with siRNA in HCT116 and DLD1 cells. **(M, N)** The proliferation ability of HT116 and LoVo cells after silencing PSMC5^△2.1^ expression. **(O, P)** After coculturing colon cancer cells with THP-1-derived macrophages for 48 h by Transwell assay, we detected the relative mRNA levels of iNOS, CD86, CD206 and CD163 by qRT–PCR **(G)**. *p < 0.05 and **p < 0.01.

As shown in **Figures 9A, B**, we found that in HCT-116 and SW480 cells, the ratio of CD46^△13^ transcript vs. CD46^13+^ was higher than that in other colon cancer cells. Therefore, in the next experiments, we used HCT-116 and SW480 cells to explore the biological roles of CD46-9652-ES. Small interfering RNAs (siRNAs) were utilized to specifically silence CD46^△13^ and CD46^13+^ expression. After transfecting these siRNAs, we found that the expression of CD46^△13^ and CD46^13+^ was significantly downregulated (**Figures 10A–D**). Then, we performed Cell Counting Kit-8 (CCK-8) assays to evaluate the roles of CD46^△13^ and CD46^13+^ in the growth and proliferation of colon cancer. The cell growth curve results revealed that depletion of CD46^△13^ expression inhibited cell growth in colon cancer cell lines (**Figures 10E, F**). However, knockdown of CD46^13+^ expression did not influence the growth of colon cancer cells.

CD46 has been reported to play an important role in immune evasion (1, 2), and our bioinformatic analysis also indicates that CD46 can regulate T cell responses in colon cancer. Our study further validated the role of CD46^△13^ and CD46^13+^ in T cell responses. We cocultured activated T cells and colon cancer cells in 6-well plates for 4 days and then observed surviving cancer cells by crystal violet staining. As shown in **Figures 10G, H**, CD46^△13^ knockdown led to a significant decrease in the numbers of surviving cancer cells when colon cancer cells and T cells were cocultured. However, T cell responses to colon cancer cells exhibited no significant changes when CD46^13+^ expression was knocked down.

In HCT-116 and DLD1 cells, the ratios of PSMC5^△2.1^ transcript vs. PSMC5^2.1+^ were higher than those of other colon cancer cells (**Figures 9A, B**). We analyzed whether PSMC5^△2.1^ and PSMC5^2.1+^ affect cell proliferation using HCT-116 and DLD1 cell lines. The siRNAs were designed, and their efficiency was then verified (**Figures 10I–L**). CCK-8 assays showed that knockdown of PSMC5^△2.1^ expression significantly inhibited the proliferation of HCT-116 and DLD1 cells (**Figures 10M, N**). However, there were no significant effects on colon cancer cell proliferation when PSMC5^2.1+^ expression was knocked down.

We also explored the relationship between PSMC5-43011-ES and T cell responses by coculturing T cells and colon cancer cells, but there were no significant changes in the staining of surviving tumour cells after silencing PSMC5^△2.1^ and PSMC5^2.1+^ expression. Our above analysis suggested that PSMC5 was possibly related to M2 macrophages. Therefore, we next explored whether PSMC5-43011-ES affects macrophage polarization. Macrophages and colon cells were cocultured in a 2-chamber culture system. qRT–PCR results showed that si-PSMC5^△2.1^ led to a reduction in the expression of M2 polarization markers (CD163 and CD206) and an increase in the expression of M1 surface markers (iNOS and CD86) in macrophages (**Figures 10O, P**). We also treated colon cancer cells with si-PSMC5^2.1+^ and detected macrophage polarization marker expression by qRT–PCR. No regular difference in macrophages was observed between the si-PSMC5^2.1+^ and control groups. These data suggested that PSMC5-43011-ES in colon cancer cells induces M2 macrophage polarization.

## Discussion

An increasing number of studies have recognized the significance of immune-related genes (IRGs) in cancer development, and survival-associated IRGs have mainly been identified by analysing differentially expressed IRGs at the level of transcription (21, 22). However, a comprehensive analysis of IRGs at the posttranscriptional level in cancer has not been performed. AS is considered an important posttranscriptional modification, and changes in the AS of IRGs can produce abnormal isoforms that participate in immune reactions (23). In this study, we developed a novel prognostic model with immune signatures based on IRG-related OS-ASEs for the first time and validated the role of OS-ASEs in the immune response to colon cancer.

Studies have indicated the feasibility of establishing prognostic risk models with OS-ASEs and using them to predict clinical outcomes. For example, Zong, Z analysed genome-wide OS-ASEs in colon cancer and established an effective prognostic model to predict patient survival outcome (24). Yuanyuan Zhang explored significant OS-ASEs and constructed a prognostic model for determining survival that exhibited good performance in evaluating the risk of mortality in patients with stomach adenocarcinoma (25). However, AS risk models based on entire genes may not reveal relationships with the immune microenvironment and may not predict the response to immunotherapy. Previous studies have reported close correlations between IRGs and the immune system in cancer (26). Therefore, we utilized comprehensive analyses of IRG-related ASEs as a novel strategy to construct robust prognostic models considering immune characteristics and treatment potential. Our prognostic models were all confirmed to be practical and to have good reliability in predicting the OS and DFS of patients. Notably, the risk score of the comprehensive model correlated with the infiltration of multiple tumour-infiltrating immune cells and the expression of HLA-D region genes and immune checkpoint genes. Moreover, our prognostic model can provide information for immunotherapy, and IRG-related OS-ASEs may represent promising targets for immunotherapy.

Immune microenvironments and the associated immune cell infiltration play crucial and intricate roles in CRC development. For example, increasing the activity of antitumour CD8+ T cells and decreasing that of pathogenic CD4+ T cells can delay CRC progression (27). Tumour-infiltrating T cells stimulate the expression of CCL5, promoting CRC metastases (28). Our study showed that AS alterations in IRGs significantly promoted the infiltration of most immune cells, such as M2 macrophages and CD4+ T cells. Furthermore, classical MHC genes are often thought to present antigenic peptides to T cells, resulting in immune T cell infiltration (29). We showed that the risk score was positively related to the expression of multiple HLA-D region genes that directly activate CD4+ T cells. The IRG-related ASE/HLA-D region gene/CD4+ T cell axis probably plays important roles in CRC prognosis. Classical studies have shown that immune checkpoint genes, represented by PD-L1 and CTLA-4, can inhibit T cell immunity and survival by counteracting immune cell-activating signals, therefore resulting in immune evasion (30–32). The risk score of our model presented a positive correlation with the expression levels of certain immune checkpoint genes, such as PD-L1 and CTLA-4, indicating that the poor prognosis of patients with a high risk score potentially results from the enhanced immune checkpoint expression caused by some IRG-related ASEs (33).

Currently, the prevailing view is that cancer patients can benefit from ICI therapy, and such therapy has achieved gratifying results (33). Notably, through risk grouping, we observed an abnormal phenomenon in which patients with specific stage disease with higher expression of immune checkpoint genes had a longer OS, suggesting that anti-PD-L1 or anti-CTLA-4 therapy possibly decreases the survival of specific patients. Recently, a study in *Nature* also reported a similar observation that anti-PD-L1 or anti-PD1 therapy reduces survival in specific hepatocellular carcinoma patients, probably as a result of abnormal T cell activation caused by ICI-mediated damage to immune surveillance (34). We further found that in our study, all of the protective functions of immune checkpoints were present in the subgroup with low risk and high immune checkpoint expression, and this subgroup had the least active T cells of the four subgroups. Therefore, in colon cancer, our bioinformatics analysis supported the findings in the *Nature* paper: ICI therapy possibly contributes to immune impairment and tissue damage as a result of abnormal T cell activation, consequently reducing the OS of patients at certain stages of disease. This interesting observation revealed a risk of ICI therapy in specific patients, which is associated with IRG-related ASEs. This finding provides effective information for excluding the population in which ICI therapy will be detrimental. In addition, our study predicted sensitivity to chemotherapeutics and certain potential targeted drugs for further study.

One gene may undergo multiple ASEs, and key ASEs have a significant effect on gene function (35). In our study, a series of immune-related assays were conducted to verify the potential roles of IRG-related OS-ASEs. First, high levels of CD46-9652-ES and PSMC5-43011-ES were proven in colon cancer. Then, CD46-9652-ES and PSMC5-43011-ES were verified to have important functions in regulating the immune cell response. CD46 was initially identified as an important human complement-regulatory protein that participates in the proteolytic inactivation of C3b and C4b (36). Soon afterwards, CD46 was found in many eukaryocytes. On CD4+ T cells, CD46 plays a crucial costimulatory role and performs multiple functions, including driving the induction of human T helper type 1 (Th1) responses (37), balancing Th1 contraction (38) and maintaining T cell homeostasis (39, 40). In tumour cells, CD46 can block the immune function mediated by the complement system and contribute to immune escape (41). However, the relationship between CD46 expression in colon cancer cells and the T cell response remains unclear. Our validation showed that the key AS of CD46 in colon cancer cells can contribute to T cell-mediated tumour cell killing. Additionally, PSMC5 was observed to be associated with the infiltration of immune cells, including M2 macrophages, in the tumour microenvironment (42), and our study further indicated that the specific AS of PSMC5 in colon cancer cells could induce M2 polarization of macrophages.

## Conclusion

In summary, this study demonstrates that immune-related AS signatures can predict the prognosis of patients with colon cancer and offer information for identifying patients who are more likely to benefit from immunotherapy and chemotherapy. Additionally, we evaluated the abundance of products derived from representative OS-ASEs and validated that OS-ASEs have important consequences for immune cell responses. Our discovery of prognostic factors highlights a novel correlation between cancer development and the immune system, providing promising therapeutic targets and prediction approaches in cancer.

## Data Availability Statement

The datasets presented in this study can be found in online repositories. The names of the repository/repositories and accession number(s) can be found in the article/**Supplementary Material**.

## Ethics Statement

The studies involving human participants were reviewed and approved by the ethics committee of The Affiliated Hospital of Xuzhou Medical University (XYFY2019-KL221-01). The patients/participants provided their written informed consent to participate in this study.

## Author Contributions

YZ, HZ and YL designed the research. YL drafted the manuscript. LX finished the experimental verification. LX, CH and JW improved the structure of this manuscript and completed the diagrams. YZ, HZ, YL, LX, CL, XD and XJ discussed and revised the manuscript. All authors read and approved the final manuscript.

## Funding

This research was supported in part by the National Natural Science Foundation of China (No. 82072729), the Natural Science Foundation of Jiangsu (BK20211606) and Xuzhou Key R&D Program (KC20064).

## Conflict of Interest

The authors declare that the research was conducted in the absence of any commercial or financial relationships that could be construed as a potential conflict of interest.

## Publisher’s Note

All claims expressed in this article are solely those of the authors and do not necessarily represent those of their affiliated organizations, or those of the publisher, the editors and the reviewers. Any product that may be evaluated in this article, or claim that may be made by its manufacturer, is not guaranteed or endorsed by the publisher.

## Supplementary Material

Supplementary Figure 1 Specific prognostic risk models based on six types of AS. Kaplan–Meier curves and the 1-, 3-, and 5-year ROC plots of the prognostic models for predicting OS outcomes based on six types of AS, including AA **(A)**, AT **(B)**, AD **(C)**, ES **(D)**, AP **(E)**, and RI **(F)**.

Supplementary Figure 2 Cox regression analysis for evaluating the independent prognostic value of the risk score of six specific risk models. Univariate Cox regression and multivariate Cox regression analyses of clinical parameters and the risk score, in which the p values of the risk score were both less than 0.001, indicated that the risk score of AA **(A)**, AD **(B)**, AP **(C)**, AT **(D)**, ES **(E)**, and RI **(F)** can act as an independent prognostic factor in colon cancer.
