# Supplementary figures and images for: Identification and Validation of Novel Immune-Related Alternative Splicing Signatures as a Prognostic Model for Colon Cancer
Source: Front Oncol. 2022 May 26;12:866289. doi: 10.3389/fonc.2022.866289 (PMC9178000; doi:10.3389/fonc.2022.866289)

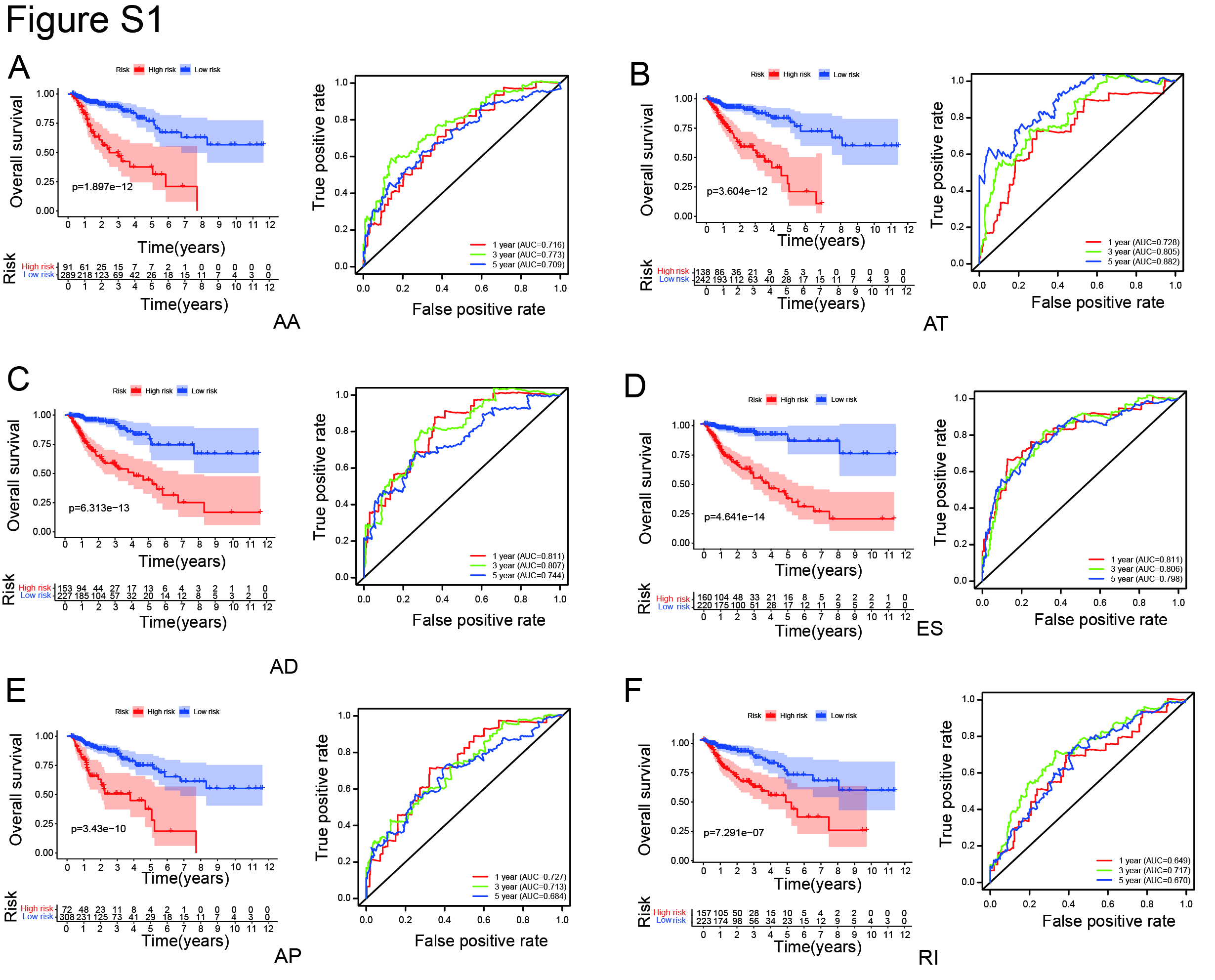

Supplement: Supplementary Figure 1 — Specific prognostic risk models based on six types of AS. Kaplan–Meier curves and the 1-, 3-, and 5-year ROC plots of the prognostic models for predicting OS outcomes based on six types of AS, including AA (A), AT (B), AD (C), ES (D), AP (E), and RI (F). [file Image_1.tif]

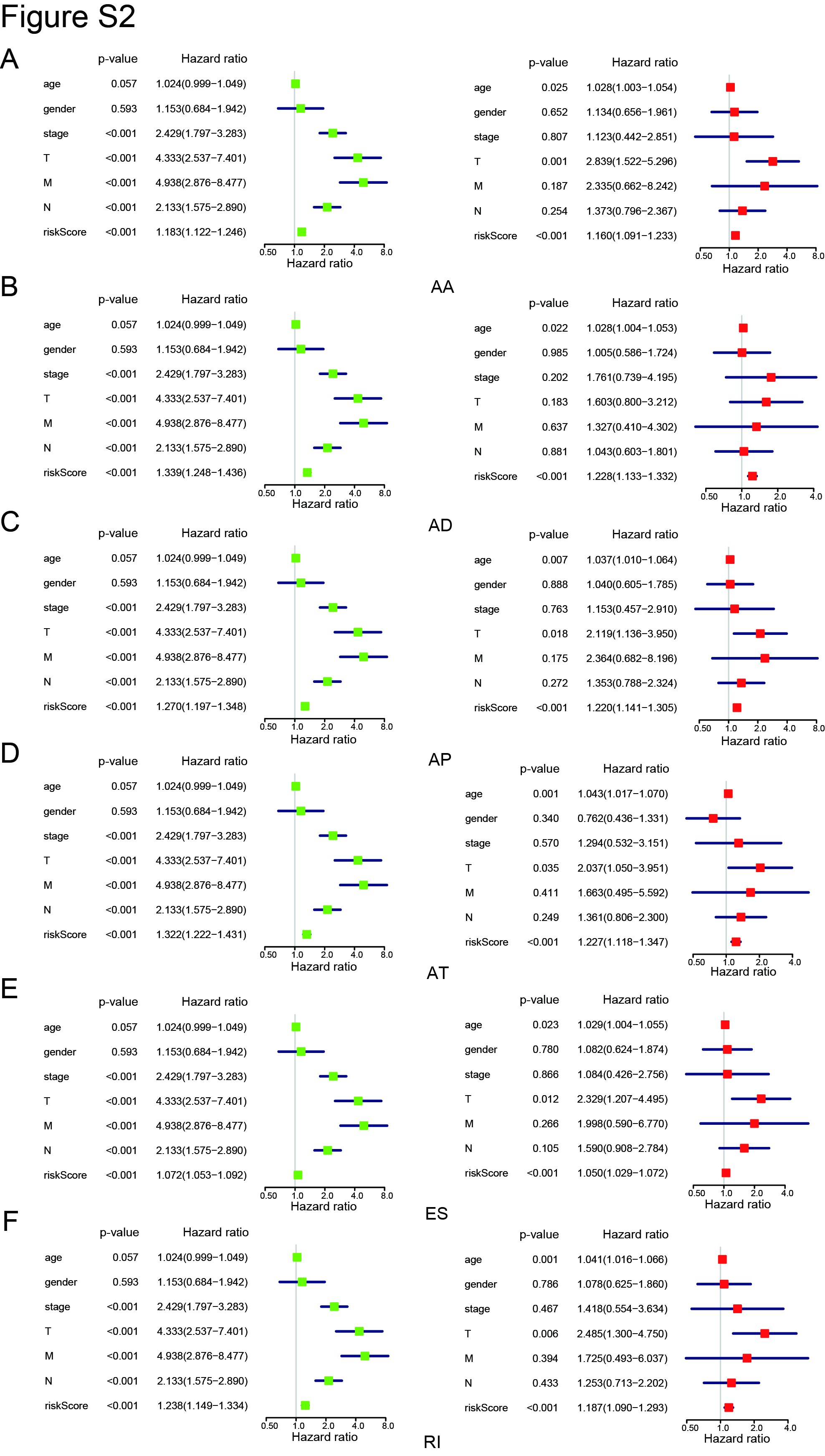

Supplement: Supplementary Figure 2 — Cox regression analysis for evaluating the independent prognostic value of the risk score of six specific risk models. Univariate Cox regression and multivariate Cox regression analyses of clinical parameters and the risk score, in which the p values of the risk score were both less than 0.001, indicated that the risk score of AA (A), AD (B), AP (C), AT (D), ES (E), and RI (F) can act as an independent prognostic factor in colon cancer. [file Image_2.tif]

Risk 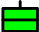 low 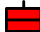 high

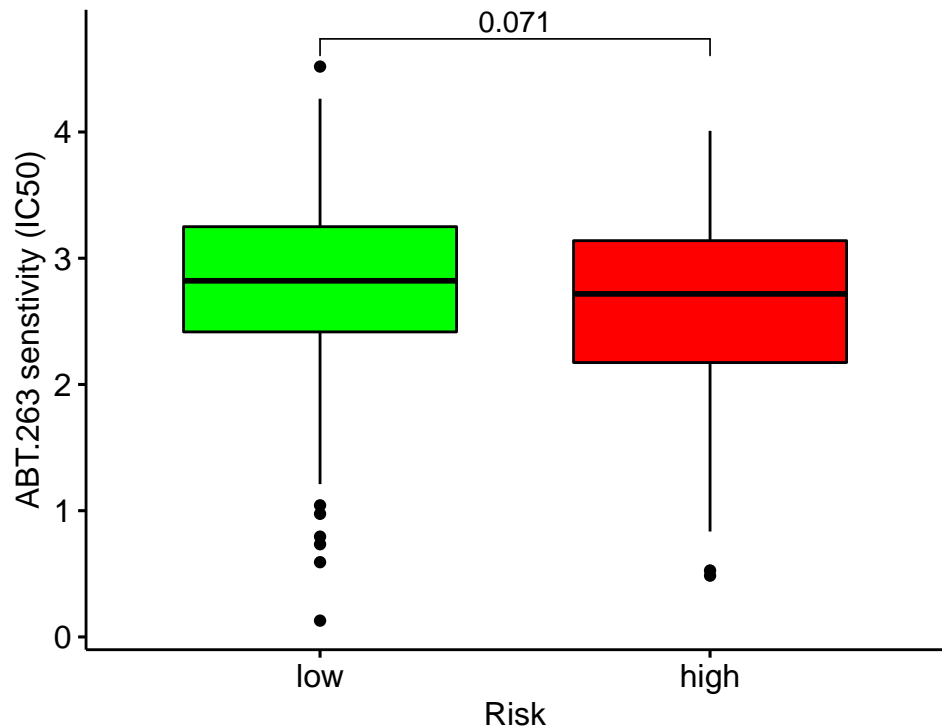

Supplement: Supplementary file 4 [file DataSheet_1.zip › Additional file 1-7/additional file 7. chemosensitivity/ABT.263 Navitoclax.pdf]

Risk 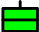 low 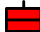 high

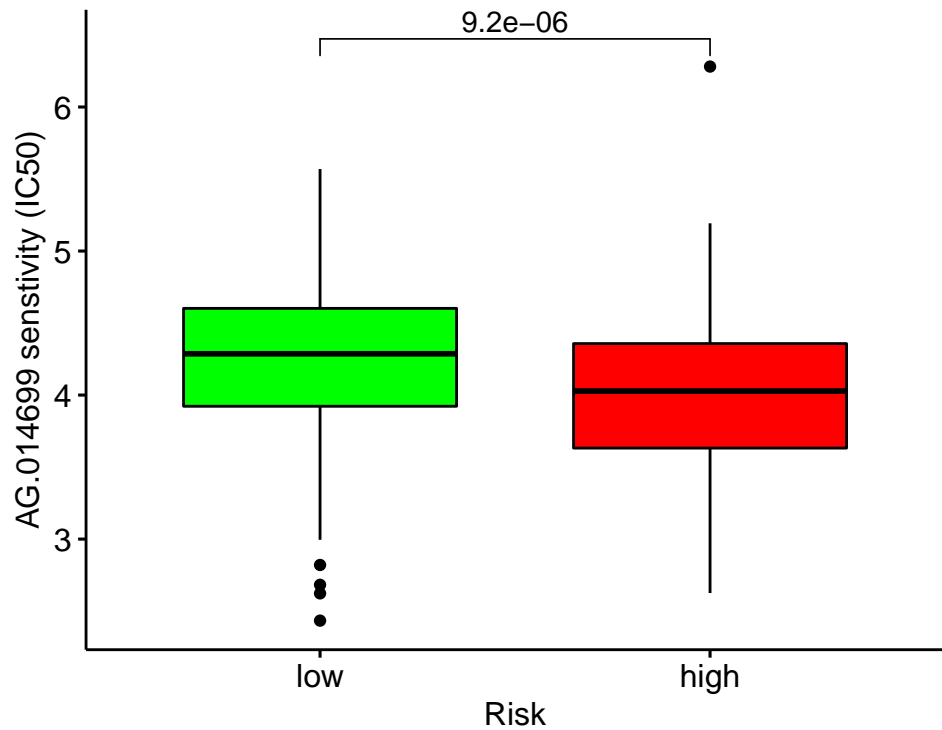

Supplement: Supplementary file 4 [file DataSheet_1.zip › Additional file 1-7/additional file 7. chemosensitivity/AG.014699.pdf]

Risk 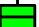 low 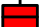 high

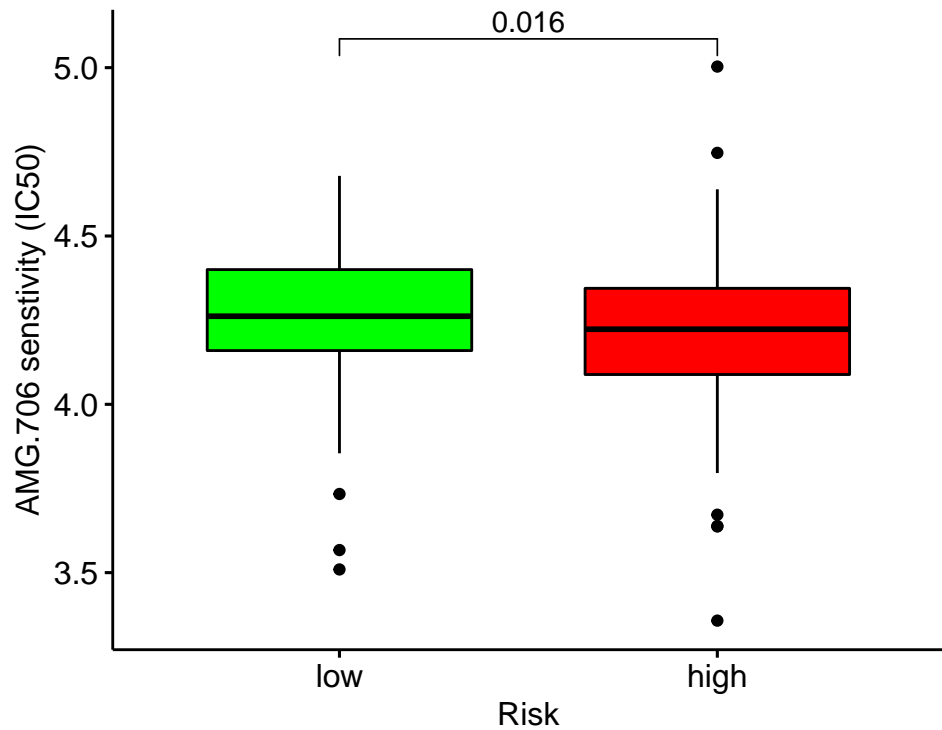

Supplement: Supplementary file 4 [file DataSheet_1.zip › Additional file 1-7/additional file 7. chemosensitivity/AMG.706.pdf]

Risk 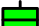 low 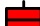 high

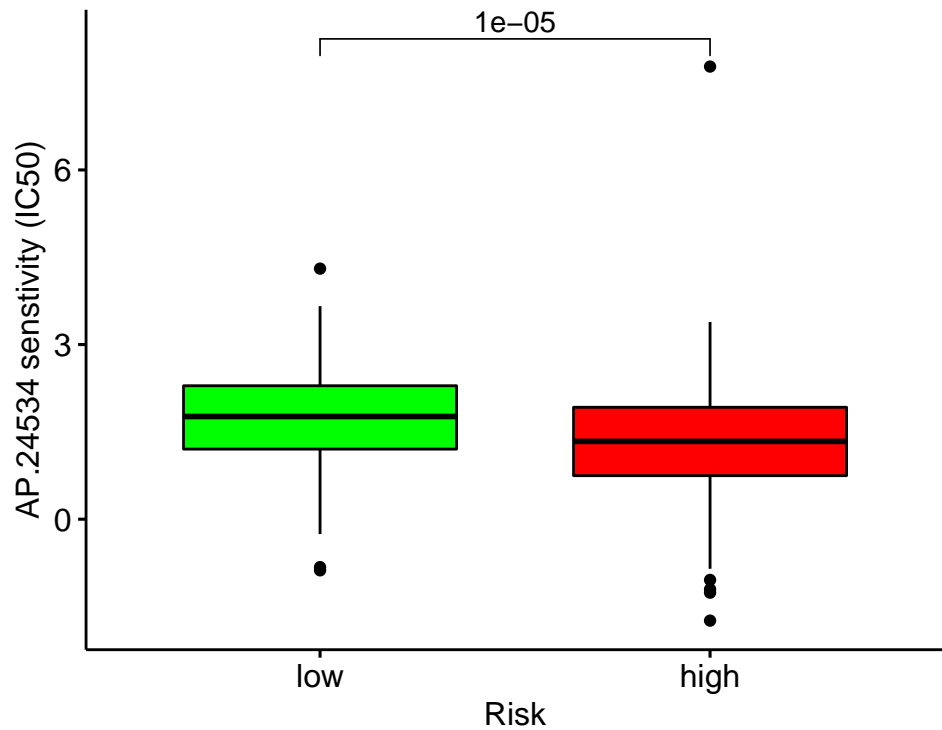

Supplement: Supplementary file 4 [file DataSheet_1.zip › Additional file 1-7/additional file 7. chemosensitivity/AP.24534.pdf]

AS601245 sensitivity (IC50)

Risk 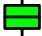 low 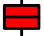 high

1.7e-06

low

high

Risk

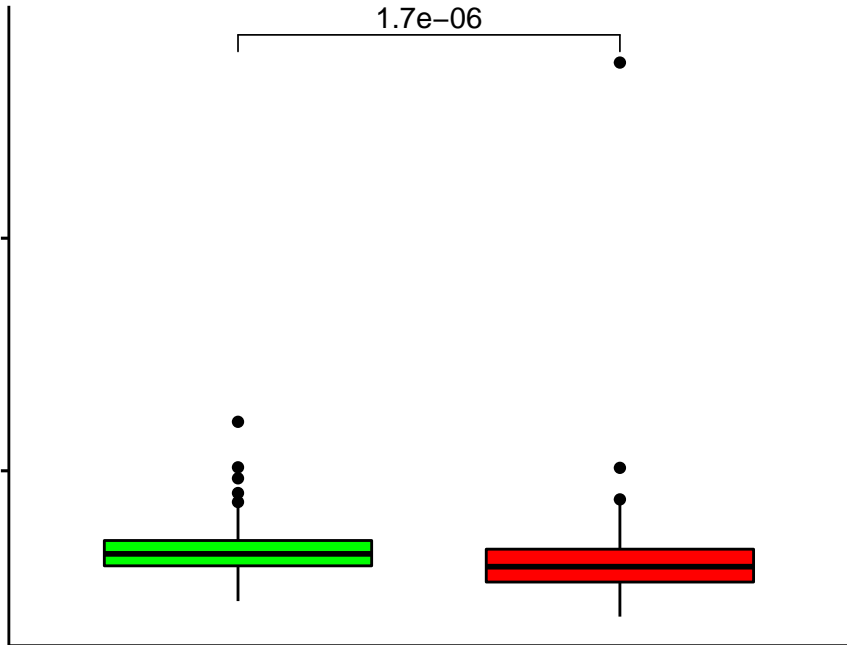

Supplement: Supplementary file 4 [file DataSheet_1.zip › Additional file 1-7/additional file 7. chemosensitivity/AS601245.pdf]

Risk 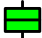 low 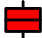 high

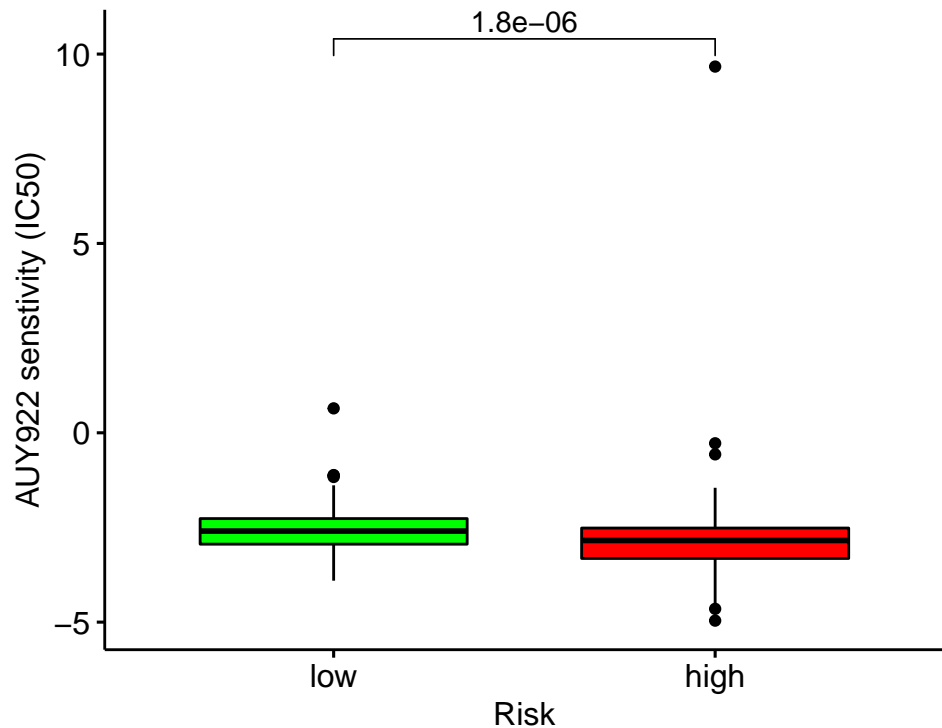

Supplement: Supplementary file 4 [file DataSheet_1.zip › Additional file 1-7/additional file 7. chemosensitivity/AUY922.pdf]

Risk 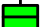 low 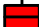 high

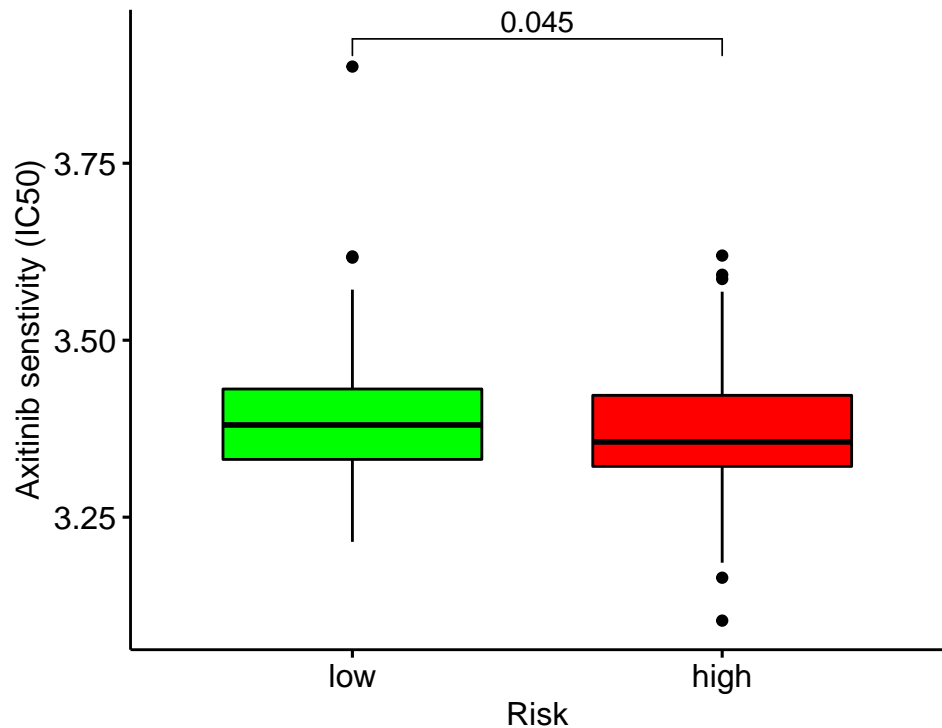

Supplement: Supplementary file 4 [file DataSheet_1.zip › Additional file 1-7/additional file 7. chemosensitivity/Axitinib.pdf]

Risk 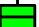 low 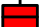 high

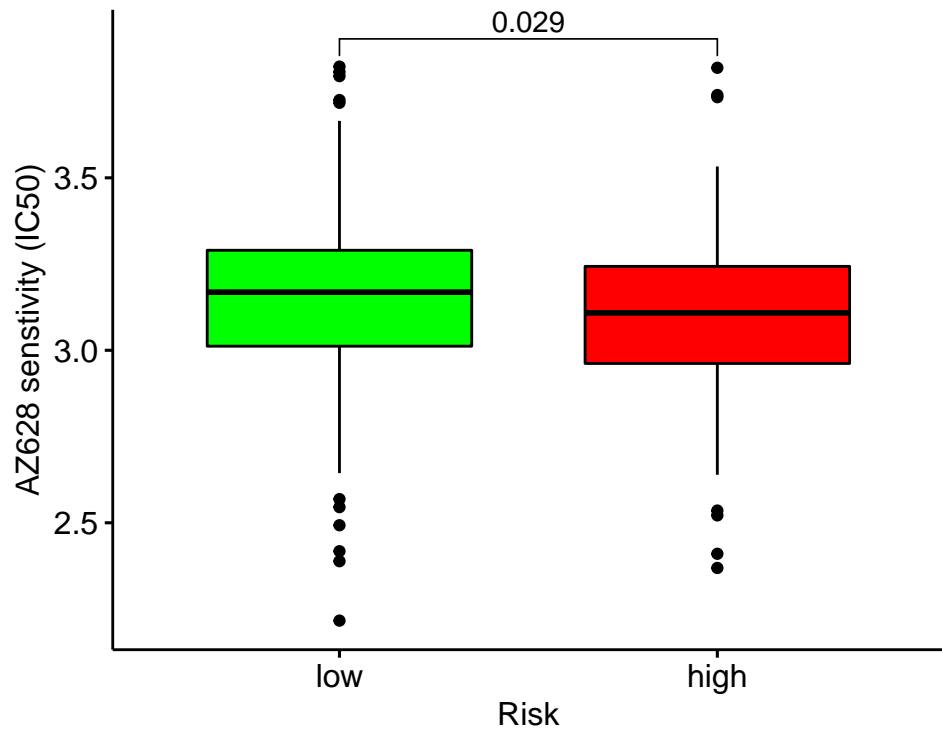

Supplement: Supplementary file 4 [file DataSheet_1.zip › Additional file 1-7/additional file 7. chemosensitivity/AZ628.pdf]

Risk 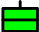 low 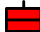 high

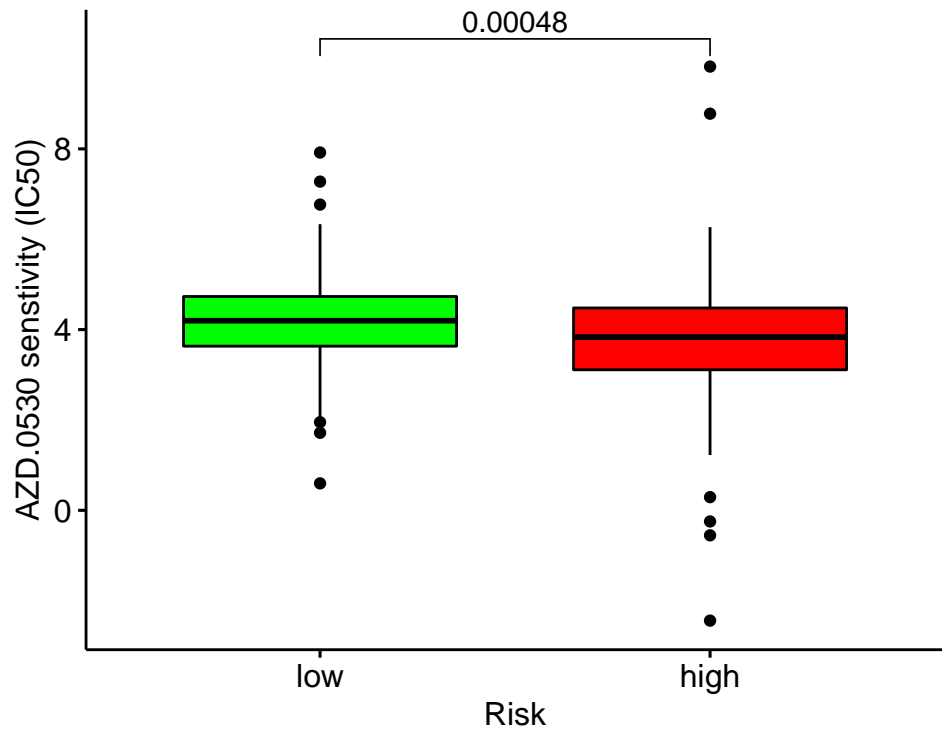

Supplement: Supplementary file 4 [file DataSheet_1.zip › Additional file 1-7/additional file 7. chemosensitivity/AZD.0530.pdf]

Risk 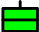 low 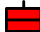 high

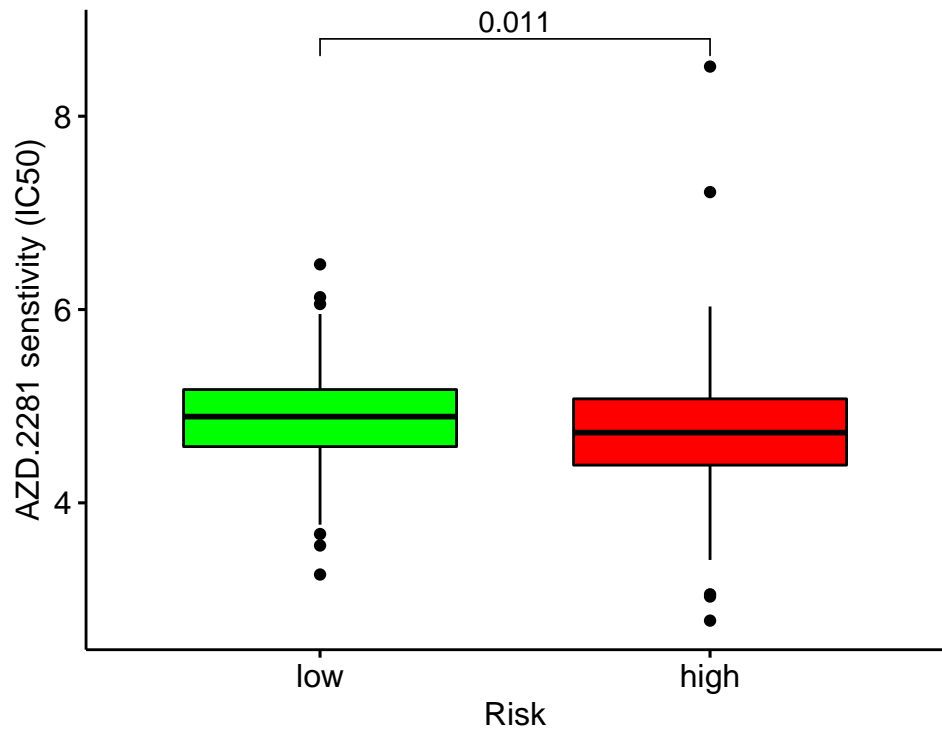

Supplement: Supplementary file 4 [file DataSheet_1.zip › Additional file 1-7/additional file 7. chemosensitivity/AZD.2281.pdf]

Risk 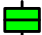 low 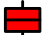 high

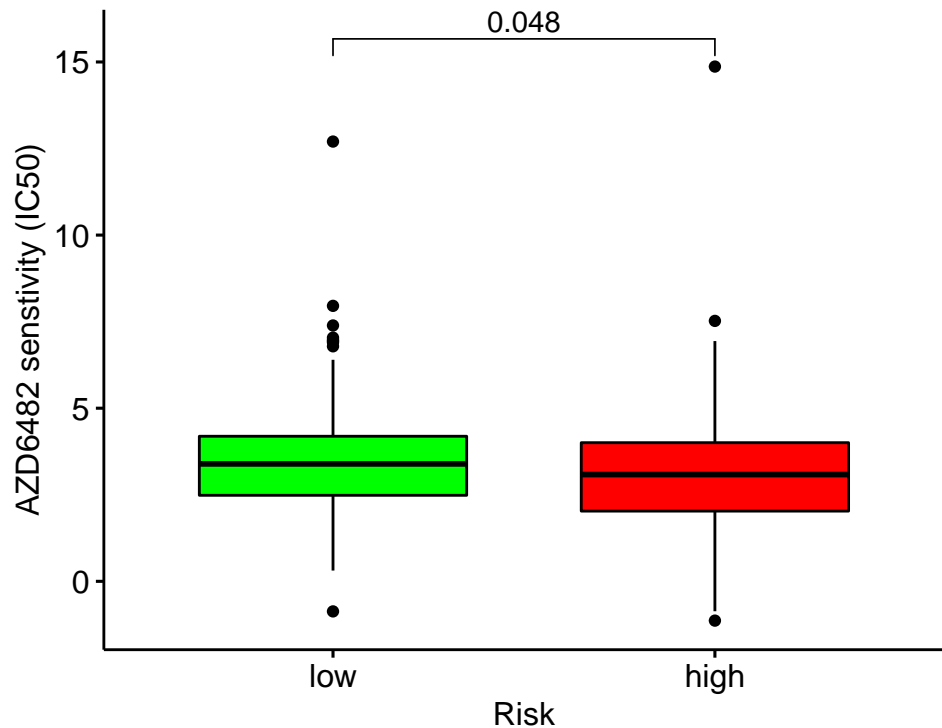

Supplement: Supplementary file 4 [file DataSheet_1.zip › Additional file 1-7/additional file 7. chemosensitivity/AZD6482.pdf]

Risk 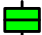 low 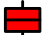 high

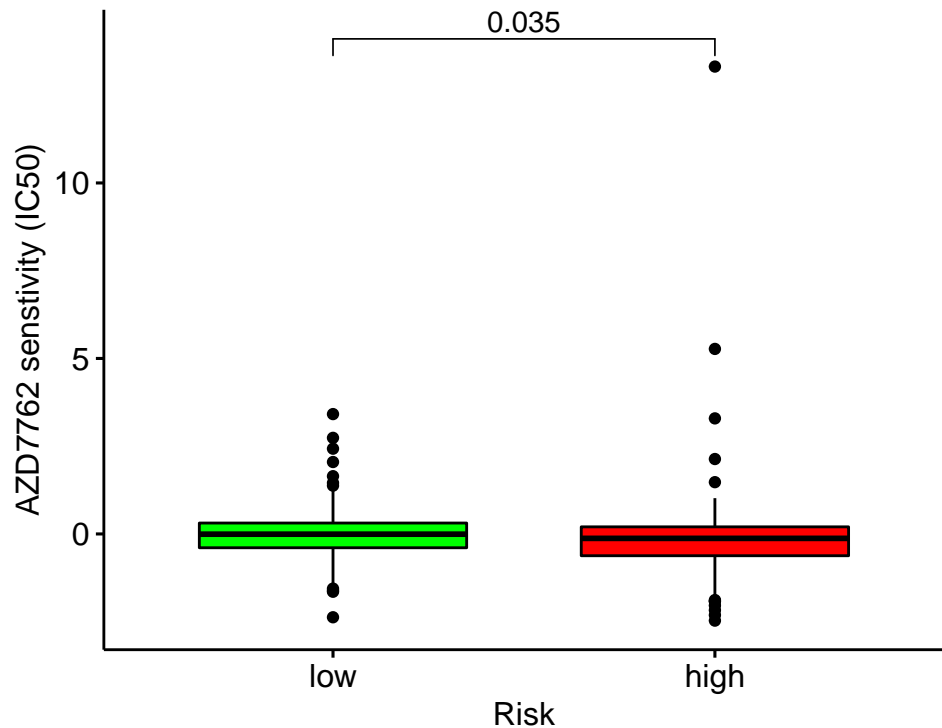

Supplement: Supplementary file 4 [file DataSheet_1.zip › Additional file 1-7/additional file 7. chemosensitivity/AZD7762.pdf]

Risk 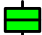 low 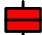 high

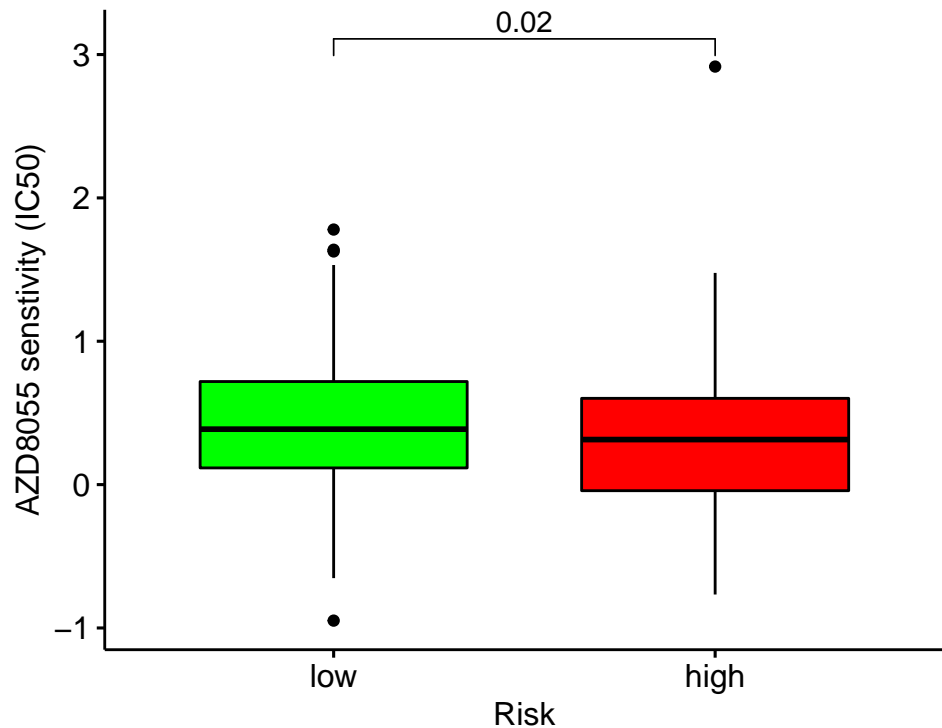

Supplement: Supplementary file 4 [file DataSheet_1.zip › Additional file 1-7/additional file 7. chemosensitivity/AZD8055.pdf]

Risk 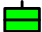 low 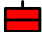 high

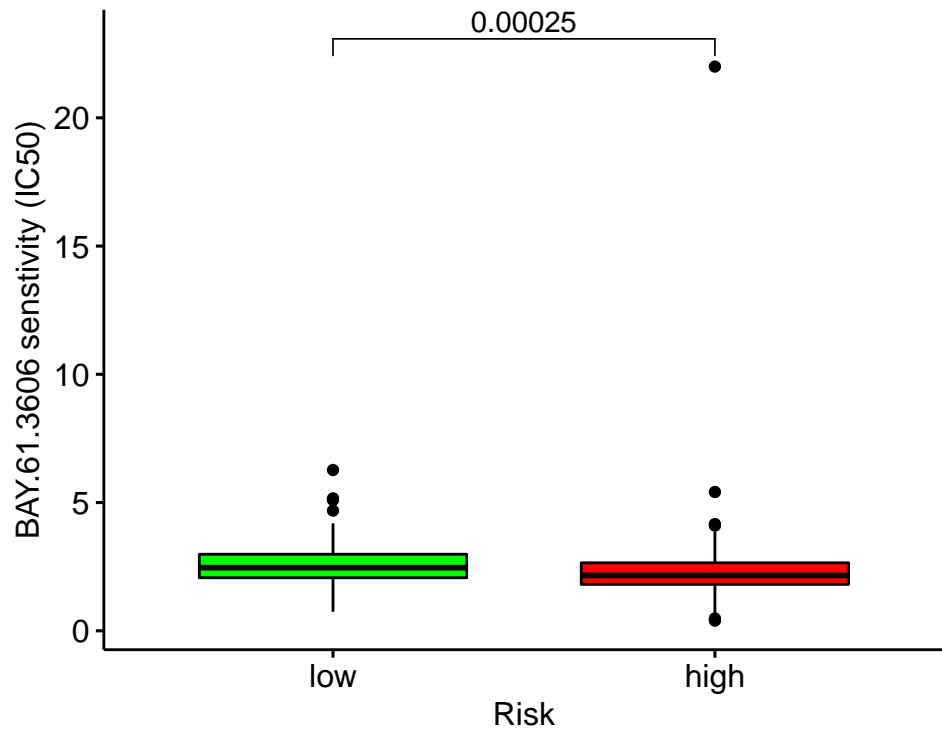

Supplement: Supplementary file 4 [file DataSheet_1.zip › Additional file 1-7/additional file 7. chemosensitivity/BAY.61.3606.pdf]

Risk 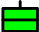 low 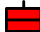 high

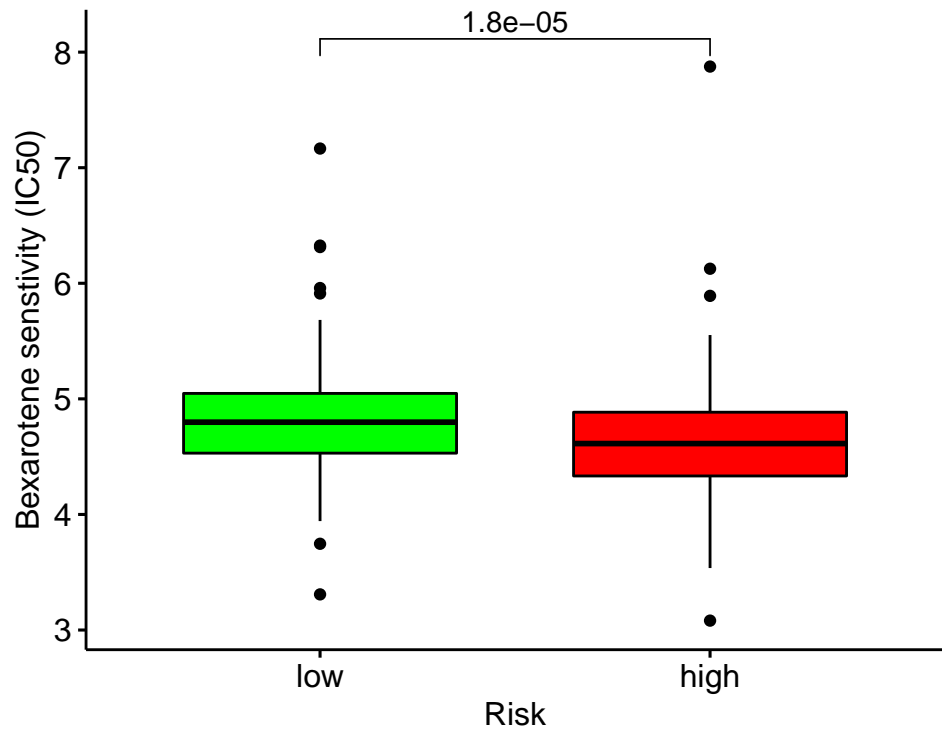

Supplement: Supplementary file 4 [file DataSheet_1.zip › Additional file 1-7/additional file 7. chemosensitivity/Bexarotene.pdf]

Risk 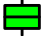 low 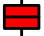 high

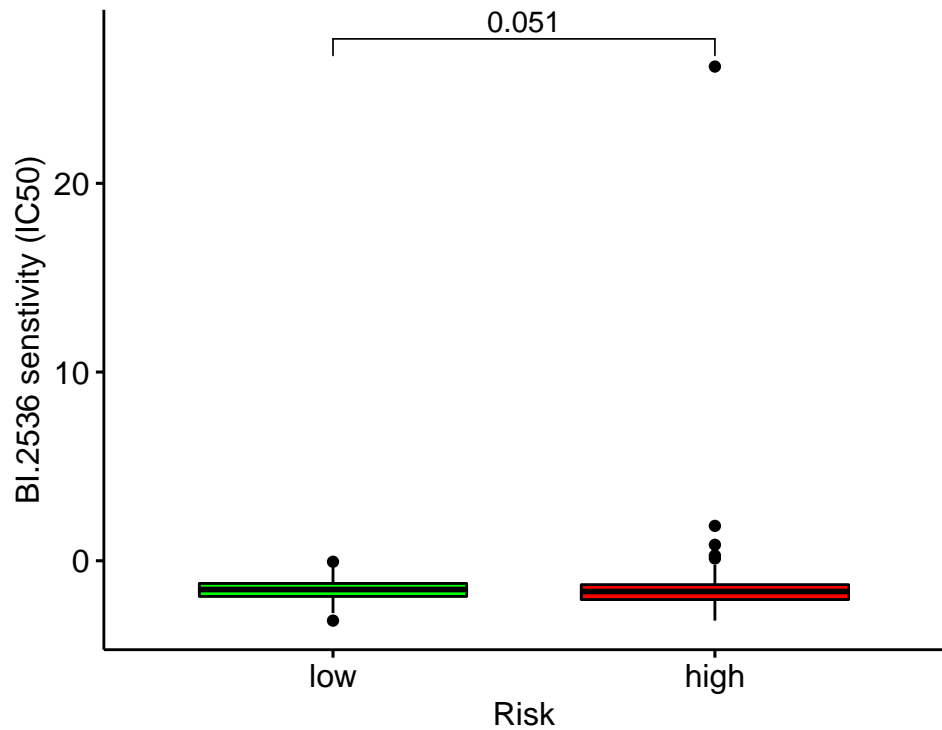

Supplement: Supplementary file 4 [file DataSheet_1.zip › Additional file 1-7/additional file 7. chemosensitivity/BI.2536.pdf]

Risk 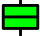 low 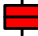 high

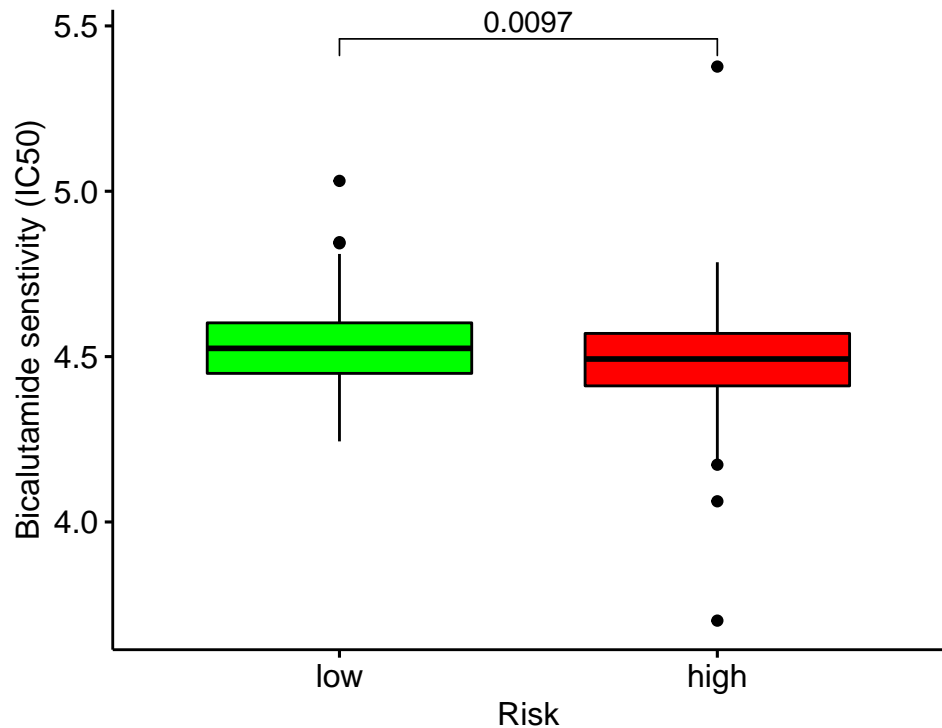

Supplement: Supplementary file 4 [file DataSheet_1.zip › Additional file 1-7/additional file 7. chemosensitivity/Bicalutamide.pdf]

Risk 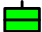 low 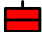 high

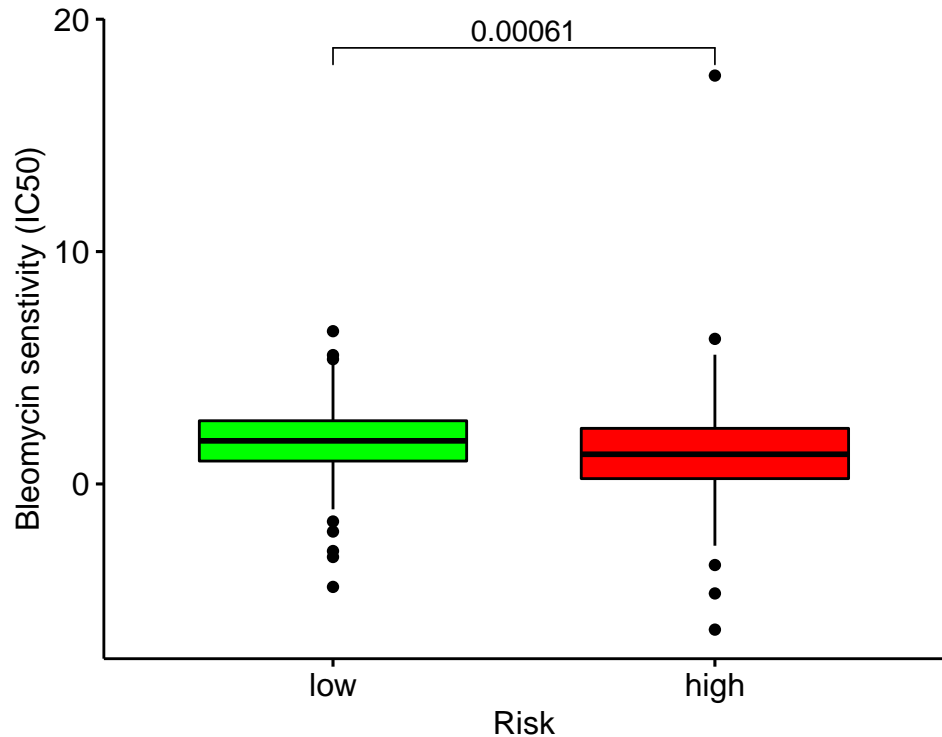

Supplement: Supplementary file 4 [file DataSheet_1.zip › Additional file 1-7/additional file 7. chemosensitivity/Bleomycin.pdf]

Risk 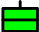 low 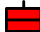 high

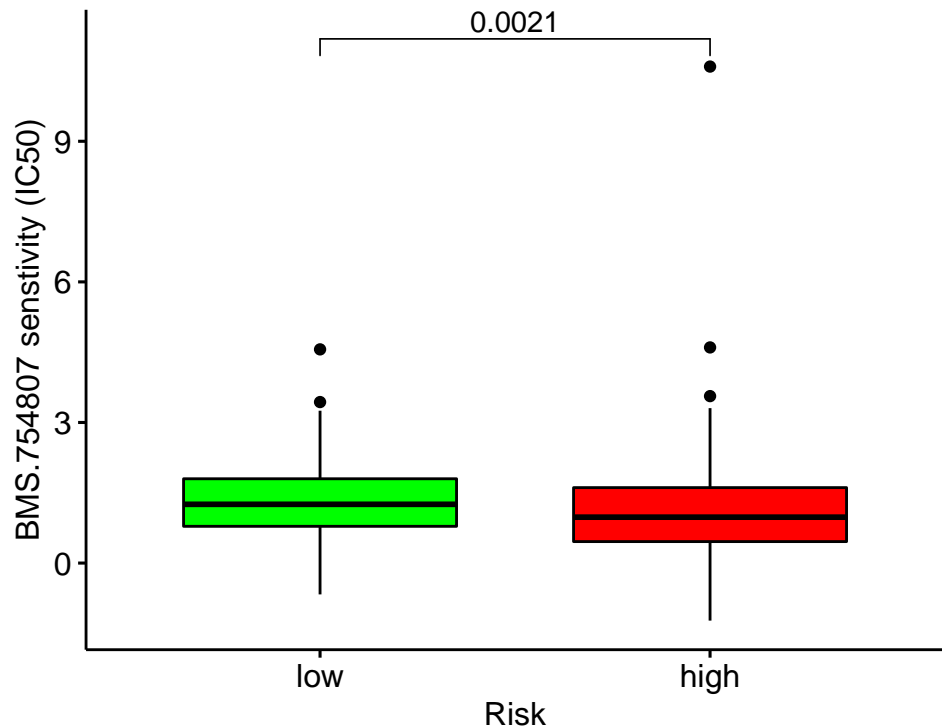

Supplement: Supplementary file 4 [file DataSheet_1.zip › Additional file 1-7/additional file 7. chemosensitivity/BMS.754807.pdf]

Risk 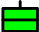 low 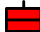 high

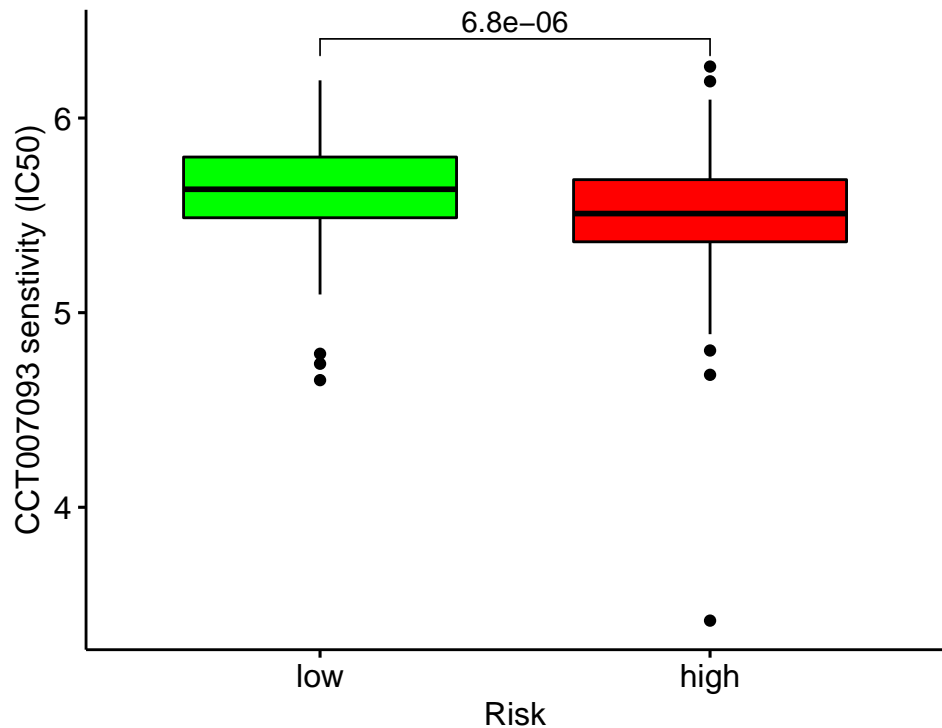

Supplement: Supplementary file 4 [file DataSheet_1.zip › Additional file 1-7/additional file 7. chemosensitivity/CCT007093.pdf]

Risk 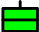 low 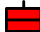 high

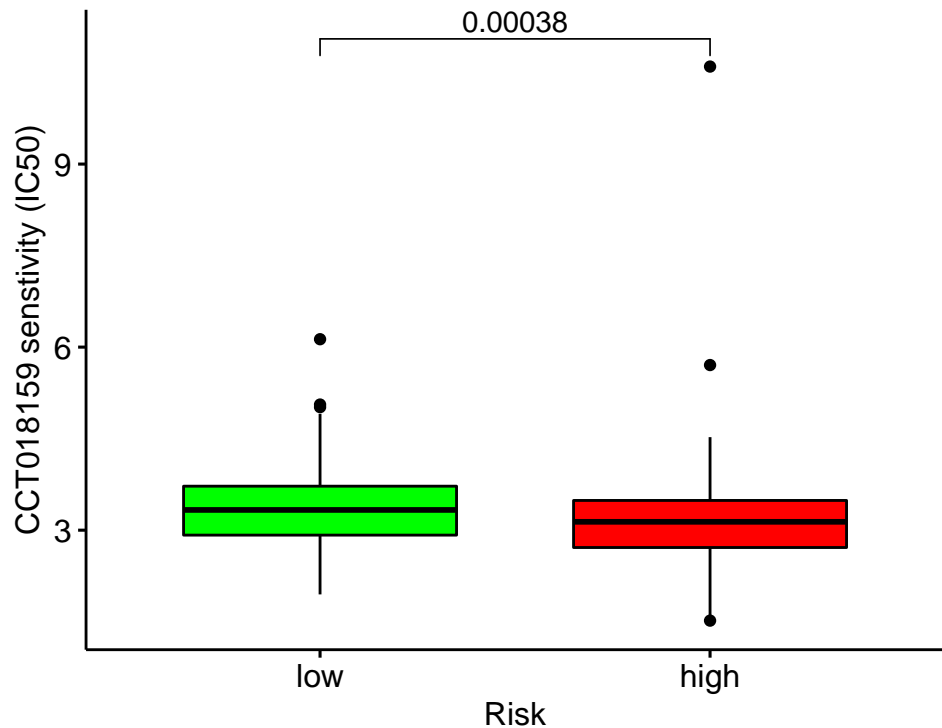

Supplement: Supplementary file 4 [file DataSheet_1.zip › Additional file 1-7/additional file 7. chemosensitivity/CCT018159.pdf]

Risk 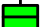 low 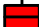 high

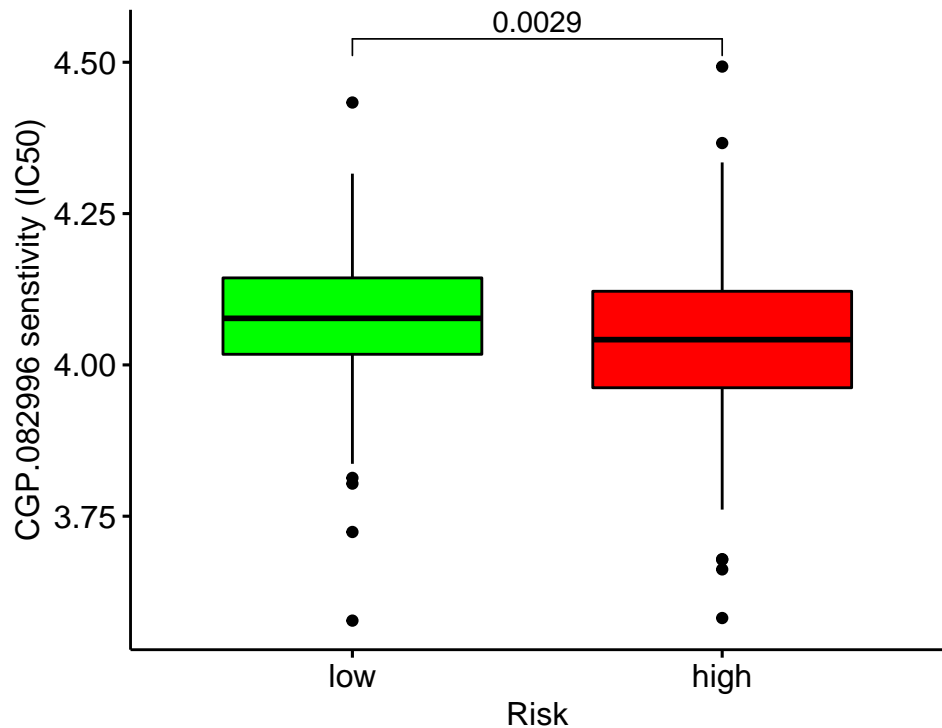

Supplement: Supplementary file 4 [file DataSheet_1.zip › Additional file 1-7/additional file 7. chemosensitivity/CGP.082996.pdf]

Risk 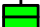 low 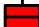 high

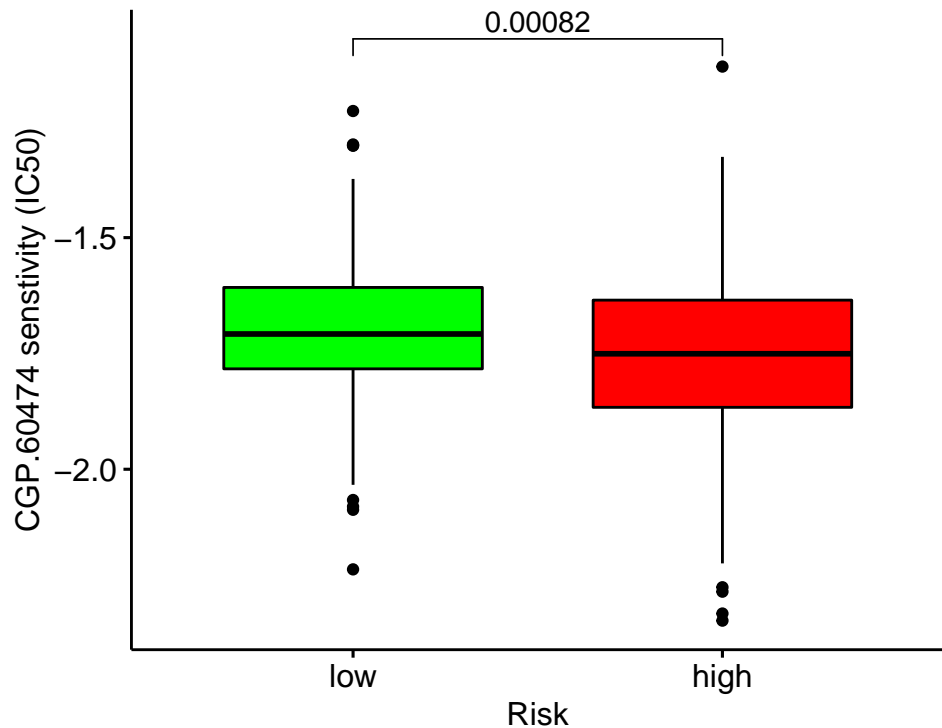

Supplement: Supplementary file 4 [file DataSheet_1.zip › Additional file 1-7/additional file 7. chemosensitivity/CGP.60474.pdf]

Risk 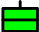 low 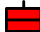 high

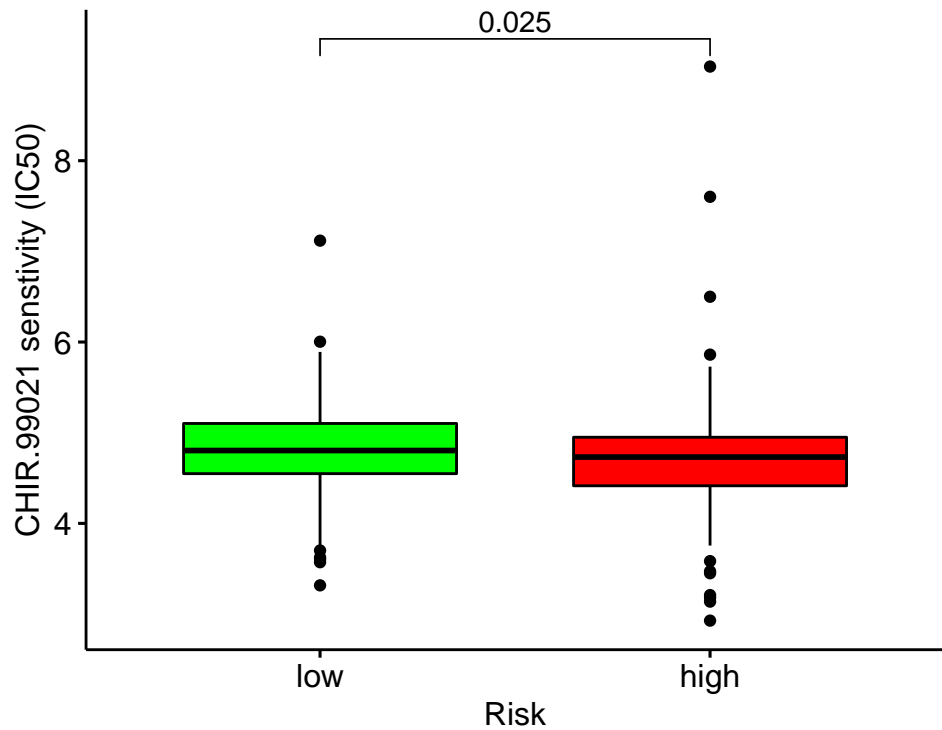

Supplement: Supplementary file 4 [file DataSheet_1.zip › Additional file 1-7/additional file 7. chemosensitivity/CHIR.99021.pdf]

Risk 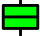 low 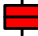 high

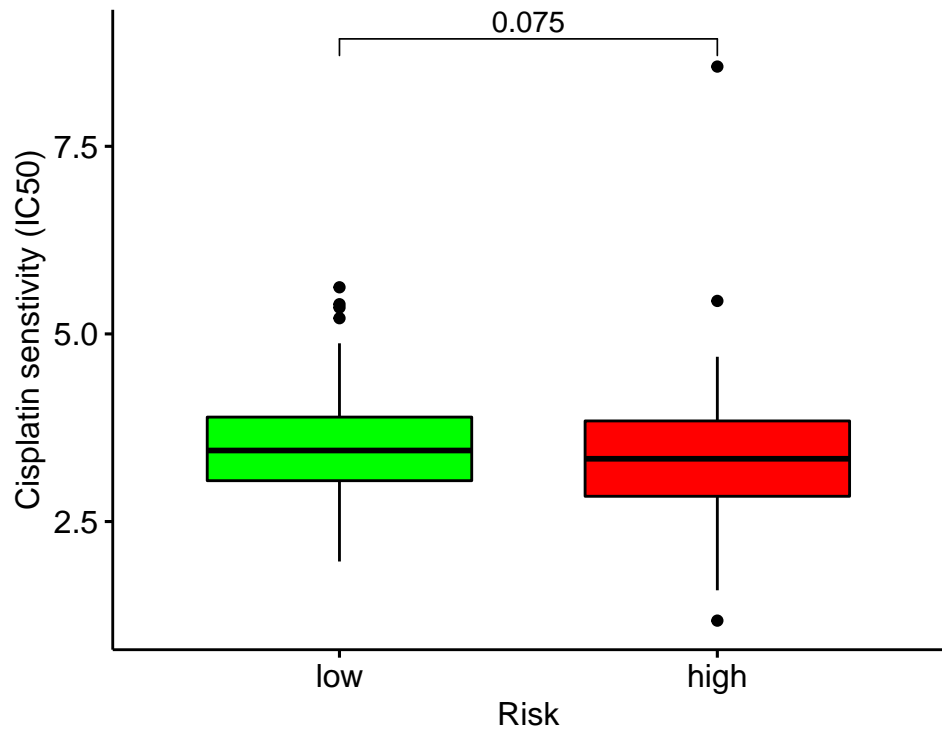

Supplement: Supplementary file 4 [file DataSheet_1.zip › Additional file 1-7/additional file 7. chemosensitivity/Cisplatin.pdf]

Risk 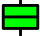 low 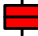 high

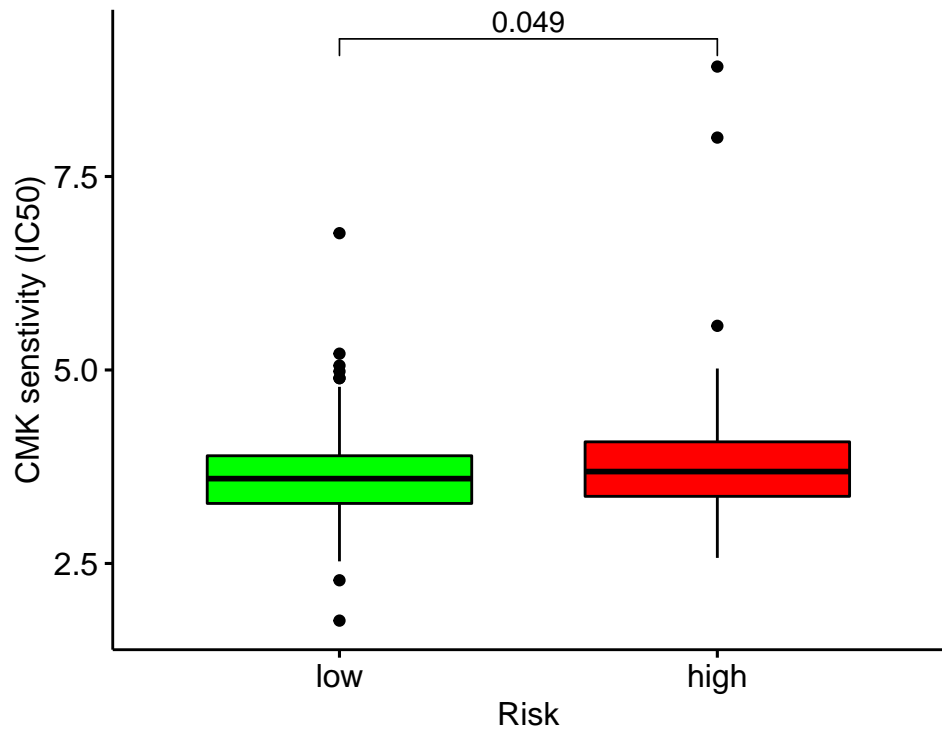

Supplement: Supplementary file 4 [file DataSheet_1.zip › Additional file 1-7/additional file 7. chemosensitivity/CMK.pdf]

Risk 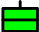 low 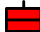 high

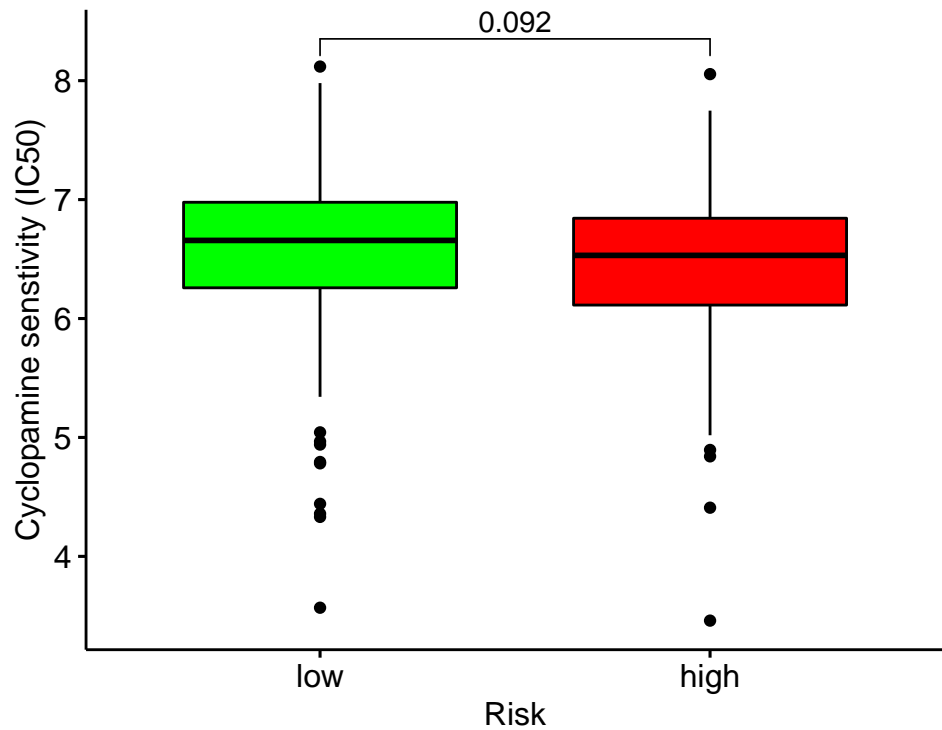

Supplement: Supplementary file 4 [file DataSheet_1.zip › Additional file 1-7/additional file 7. chemosensitivity/Cyclopamine.pdf]

Risk 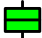 low 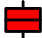 high

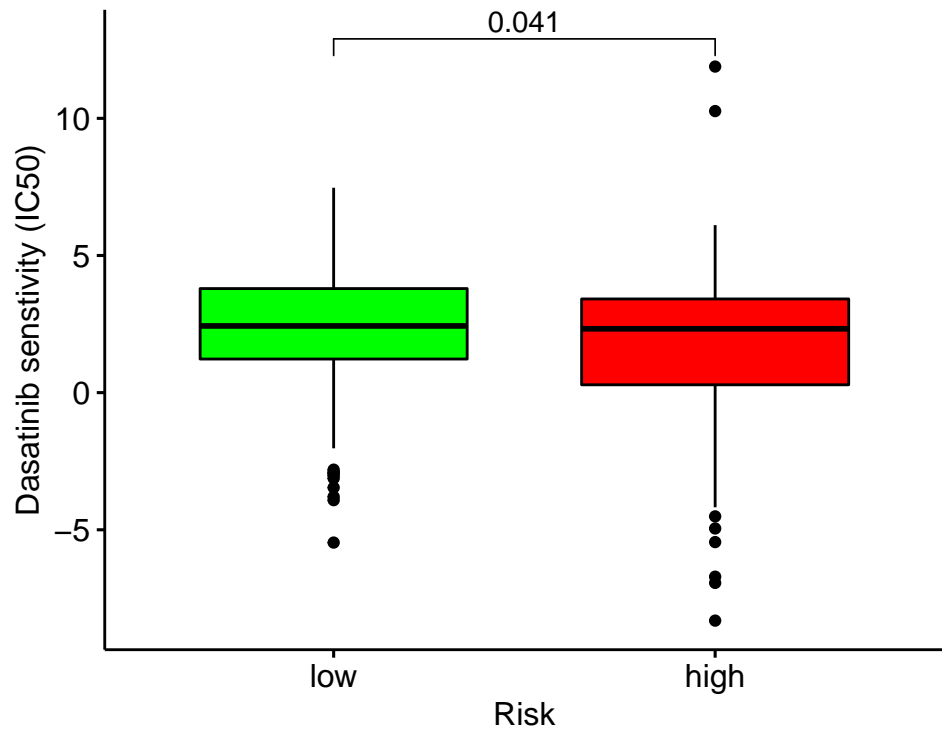

Supplement: Supplementary file 4 [file DataSheet_1.zip › Additional file 1-7/additional file 7. chemosensitivity/Dasatinib.pdf]

Risk 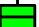 low 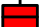 high

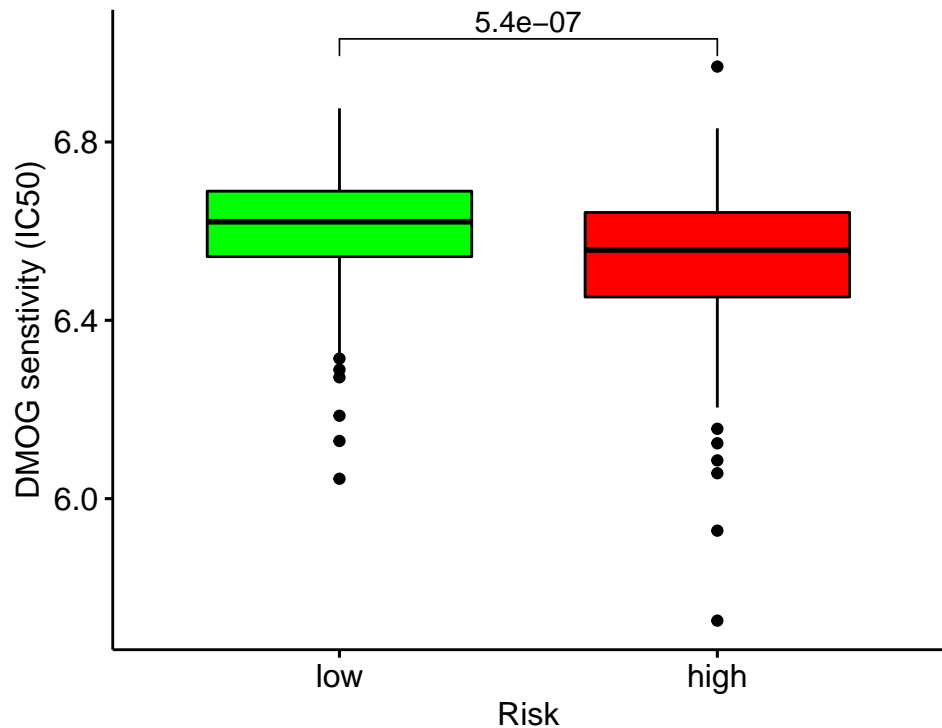

Supplement: Supplementary file 4 [file DataSheet_1.zip › Additional file 1-7/additional file 7. chemosensitivity/DMOG.pdf]

Risk 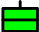 low 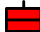 high

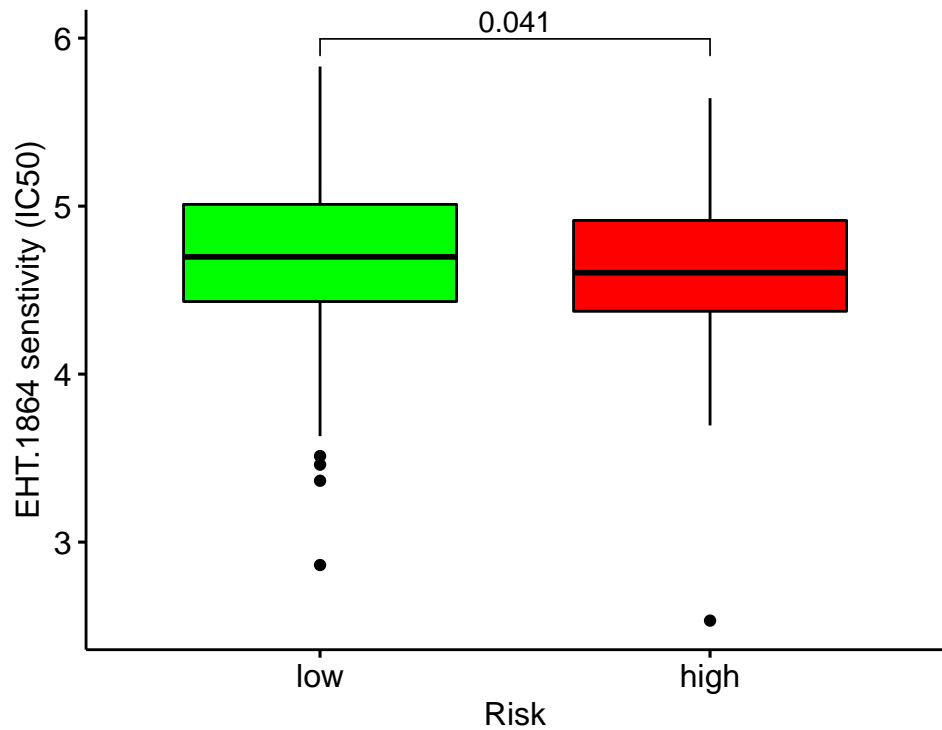

Supplement: Supplementary file 4 [file DataSheet_1.zip › Additional file 1-7/additional file 7. chemosensitivity/EHT.1864.pdf]

Risk 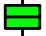 low 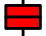 high

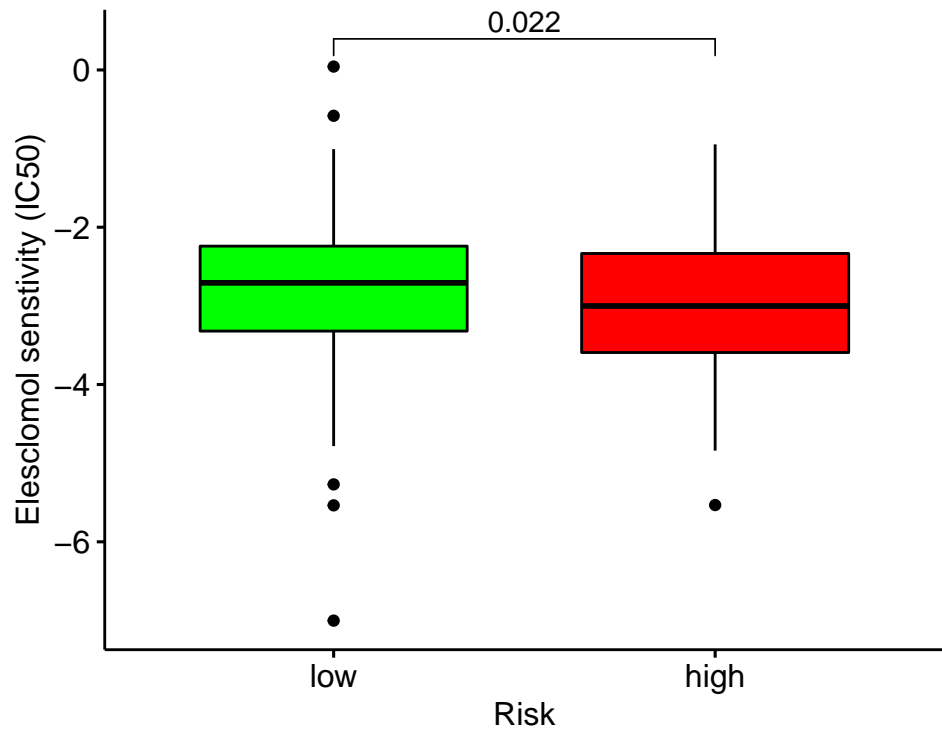

Supplement: Supplementary file 4 [file DataSheet_1.zip › Additional file 1-7/additional file 7. chemosensitivity/Elesclomol.pdf]

Risk 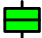 low 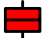 high

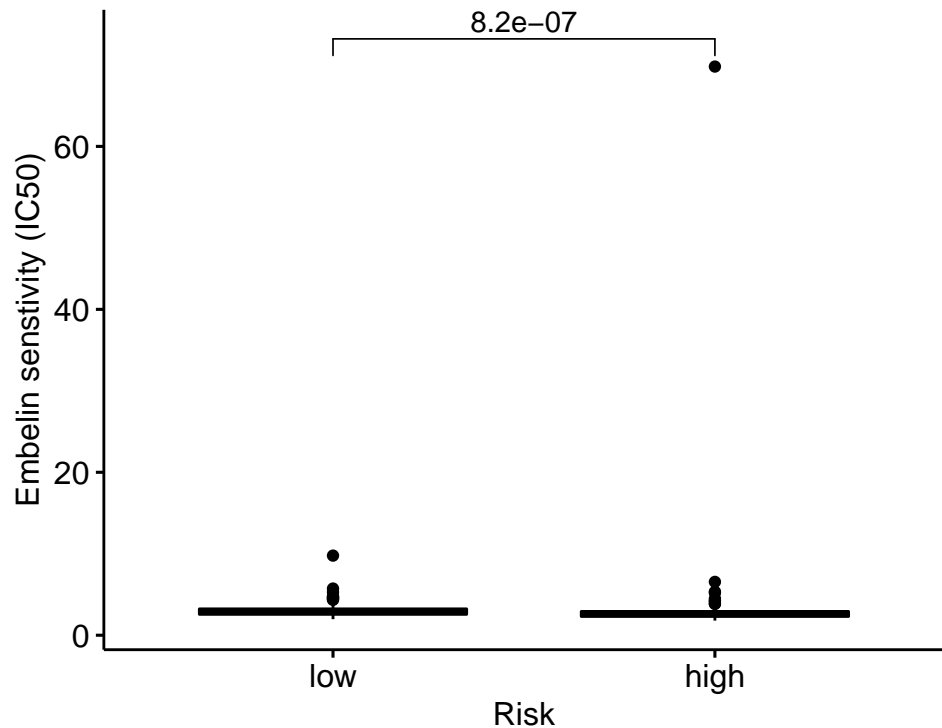

Supplement: Supplementary file 4 [file DataSheet_1.zip › Additional file 1-7/additional file 7. chemosensitivity/Embelin.pdf]

Risk 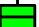 low 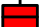 high

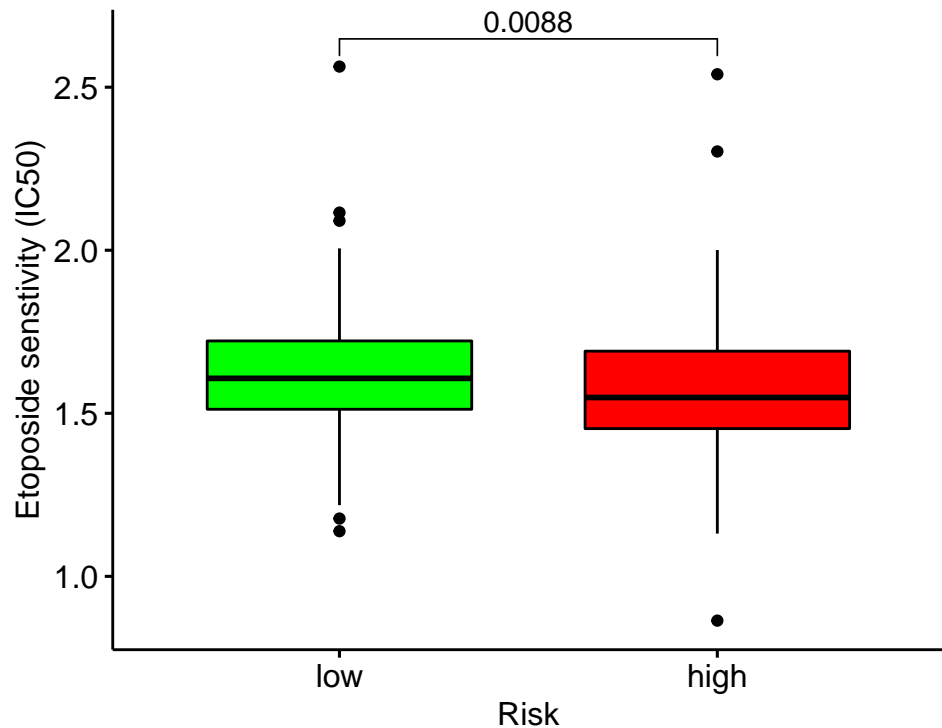

Supplement: Supplementary file 4 [file DataSheet_1.zip › Additional file 1-7/additional file 7. chemosensitivity/Etoposide.pdf]

Risk 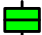 low 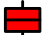 high

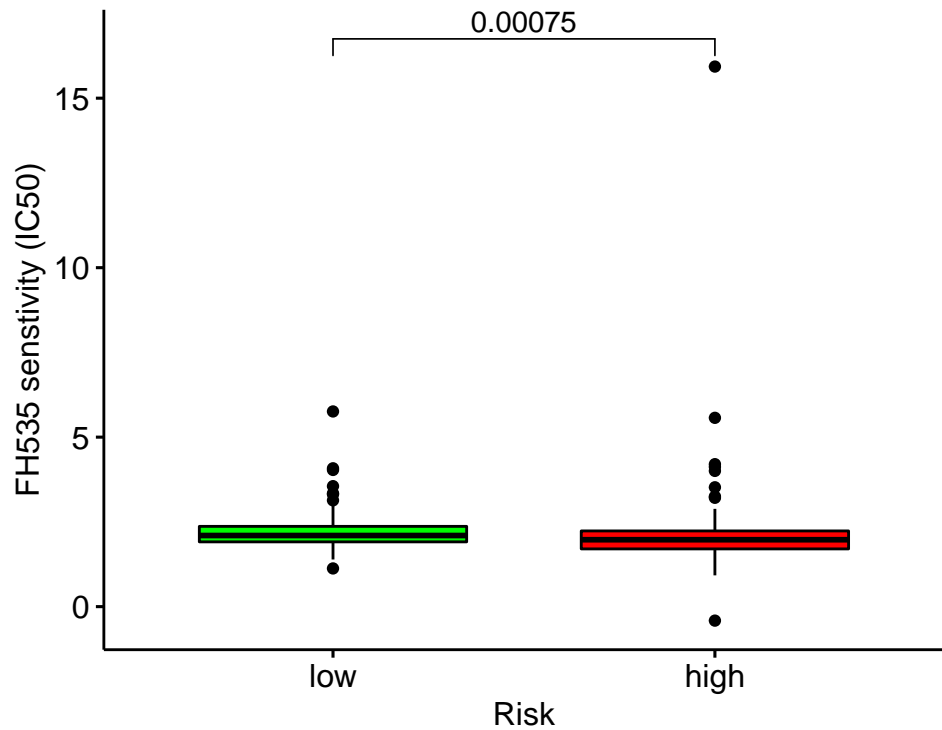

Supplement: Supplementary file 4 [file DataSheet_1.zip › Additional file 1-7/additional file 7. chemosensitivity/FH535.pdf]

Risk 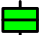 low 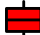 high

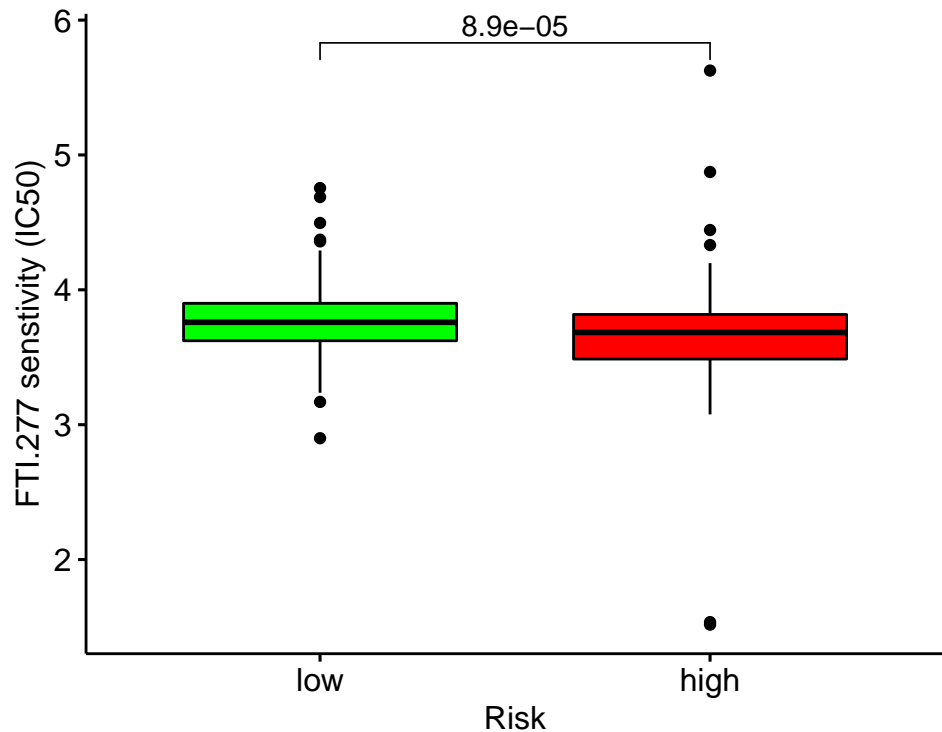

Supplement: Supplementary file 4 [file DataSheet_1.zip › Additional file 1-7/additional file 7. chemosensitivity/FTI.277.pdf]

Risk 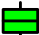 low 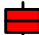 high

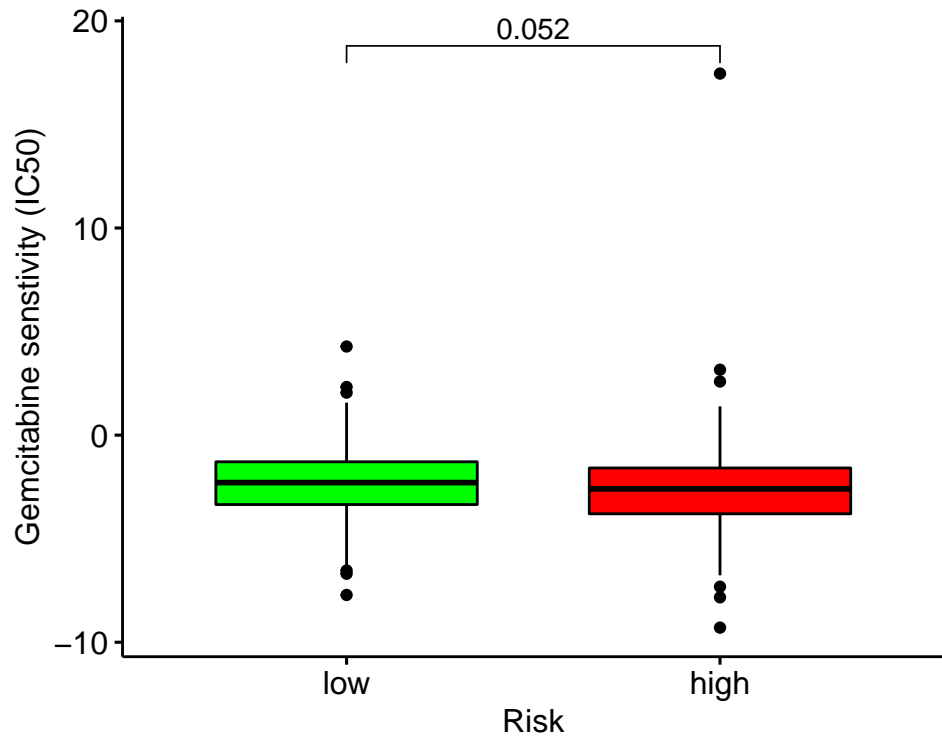

Supplement: Supplementary file 4 [file DataSheet_1.zip › Additional file 1-7/additional file 7. chemosensitivity/Gemcitabine.pdf]

Risk 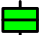 low 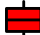 high

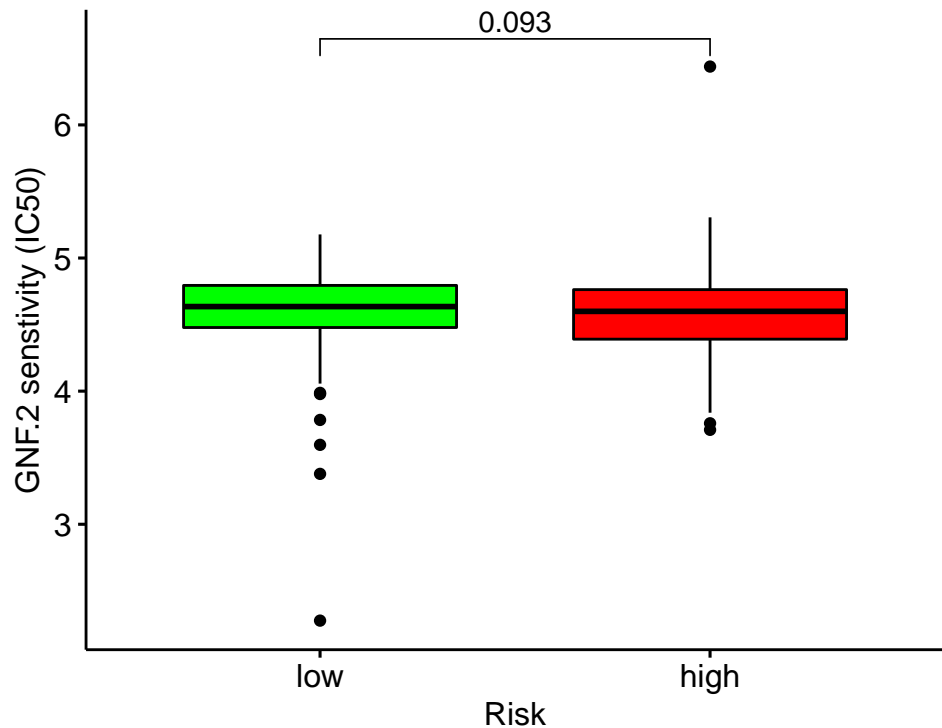

Supplement: Supplementary file 4 [file DataSheet_1.zip › Additional file 1-7/additional file 7. chemosensitivity/GNF.2.pdf]

GSK.650394 sensitivity (IC50)

Risk 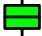 low 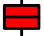 high

0.049

low

high

Risk

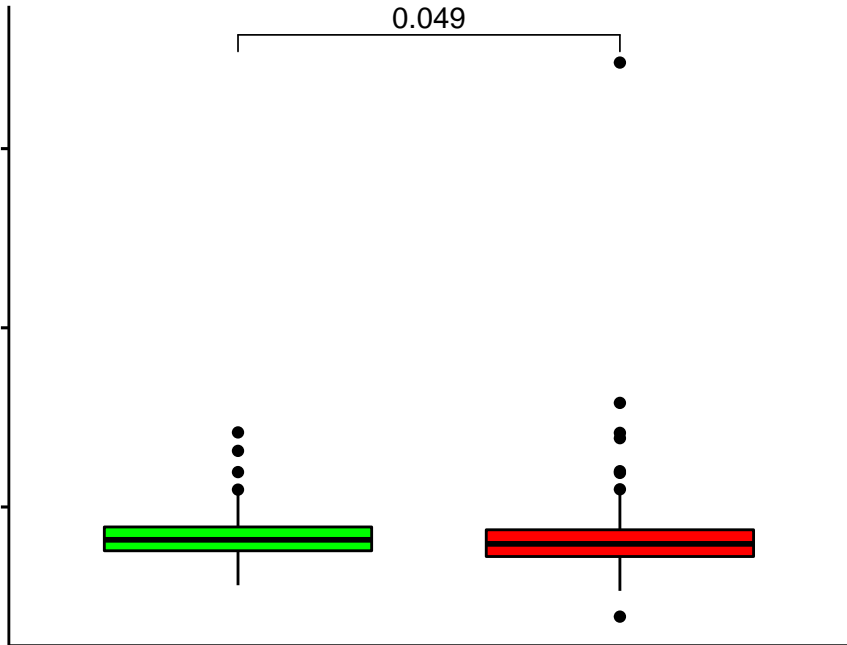

Supplement: Supplementary file 4 [file DataSheet_1.zip › Additional file 1-7/additional file 7. chemosensitivity/GSK.650394.pdf]

Risk 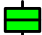 low 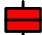 high

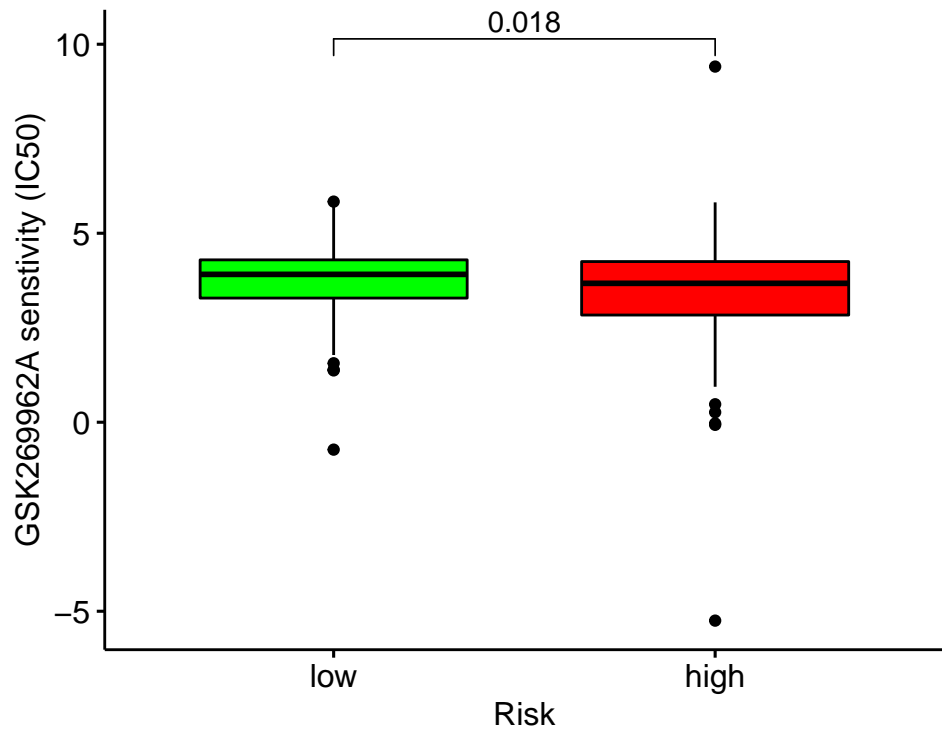

Supplement: Supplementary file 4 [file DataSheet_1.zip › Additional file 1-7/additional file 7. chemosensitivity/GSK269962A.pdf]

Risk 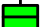 low 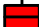 high

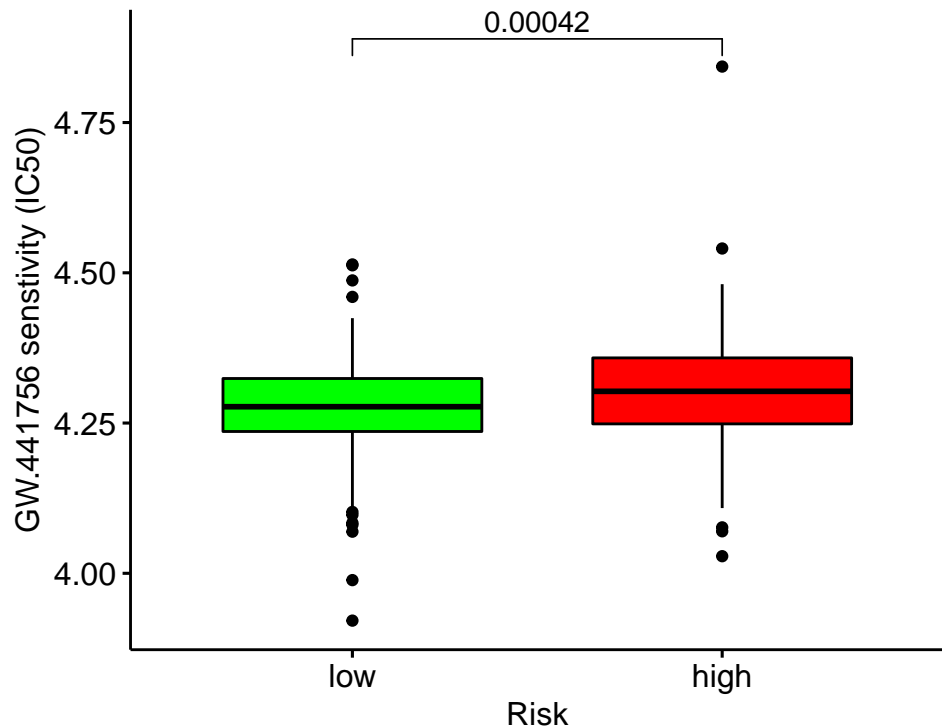

Supplement: Supplementary file 4 [file DataSheet_1.zip › Additional file 1-7/additional file 7. chemosensitivity/GW.441756.pdf]

Risk 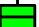 low 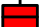 high

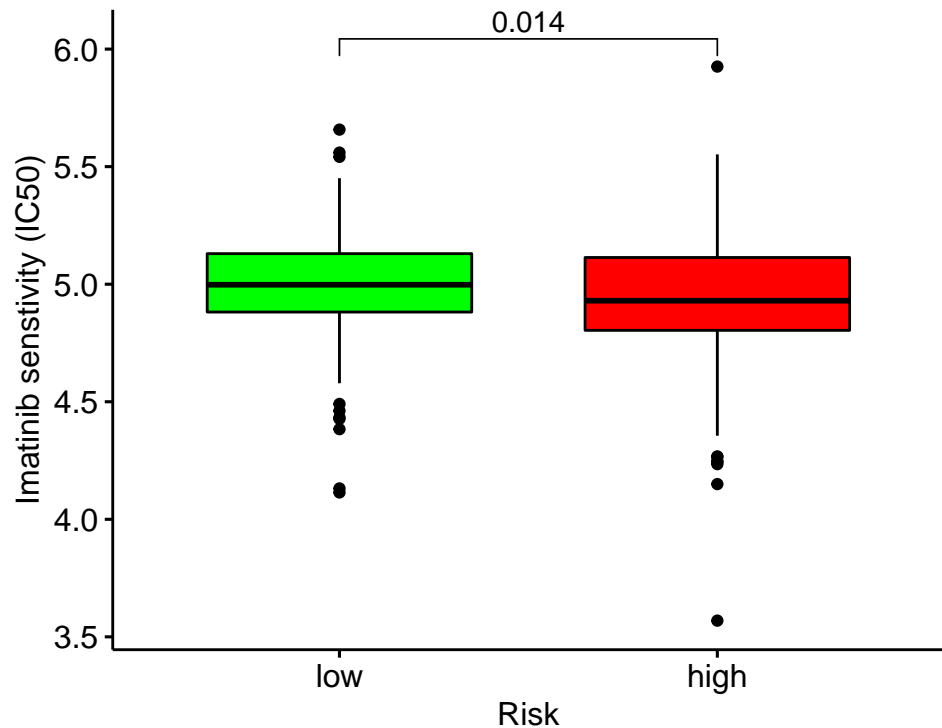

Supplement: Supplementary file 4 [file DataSheet_1.zip › Additional file 1-7/additional file 7. chemosensitivity/Imatinib.pdf]

Risk 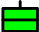 low 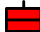 high

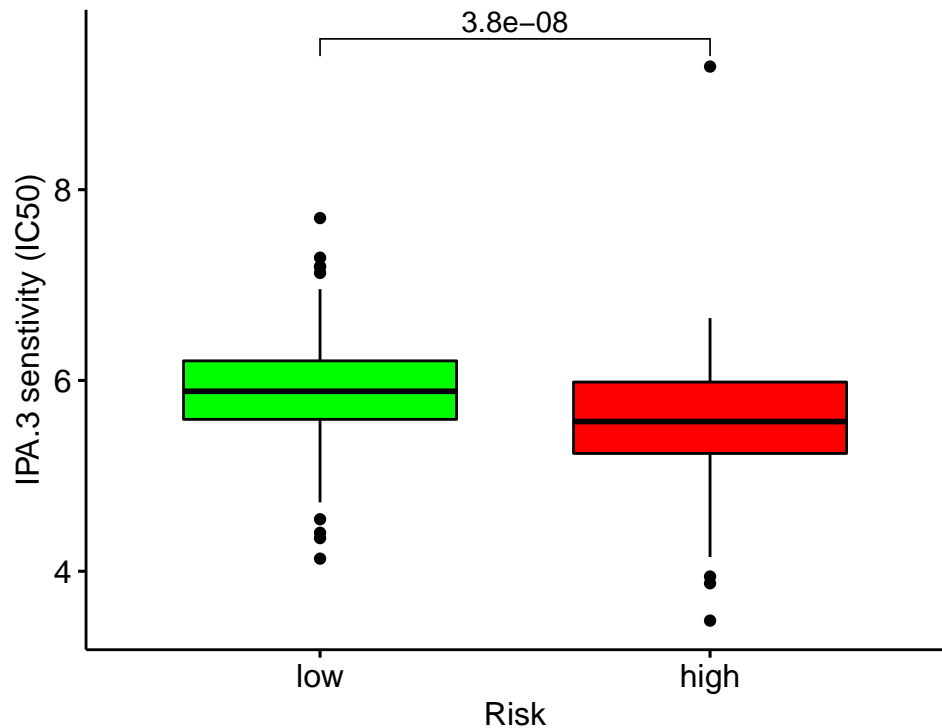

Supplement: Supplementary file 4 [file DataSheet_1.zip › Additional file 1-7/additional file 7. chemosensitivity/IPA.3.pdf]

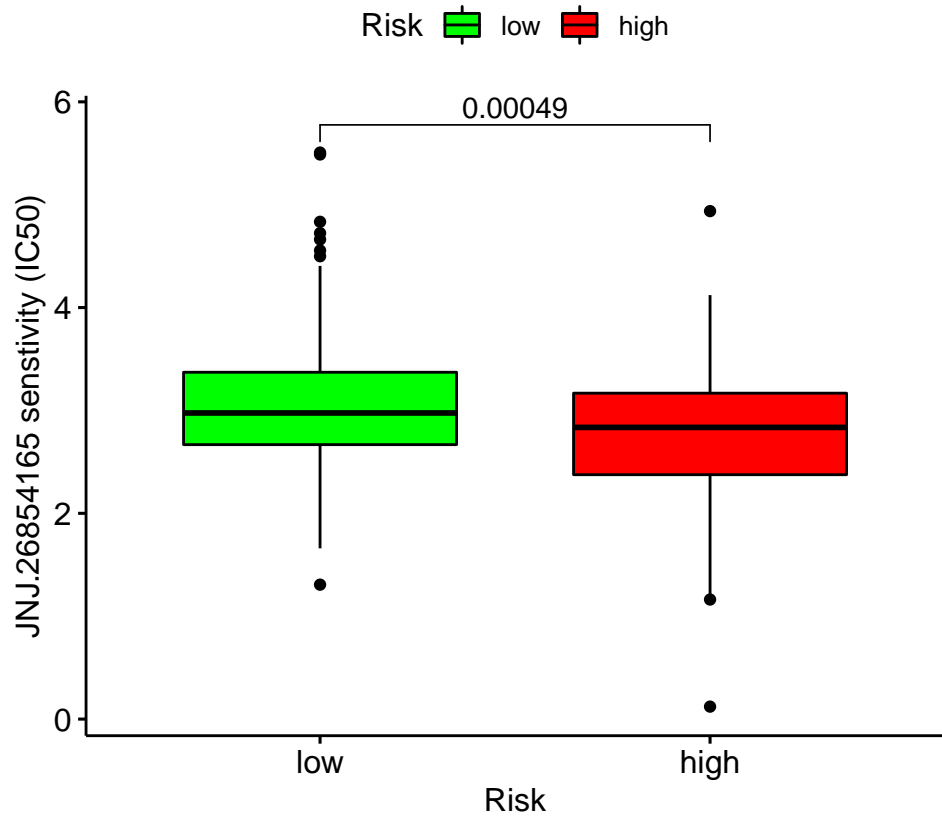

Supplement: Supplementary file 4 [file DataSheet_1.zip › Additional file 1-7/additional file 7. chemosensitivity/JNJ.26854165.pdf]

Risk 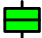 low 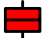 high

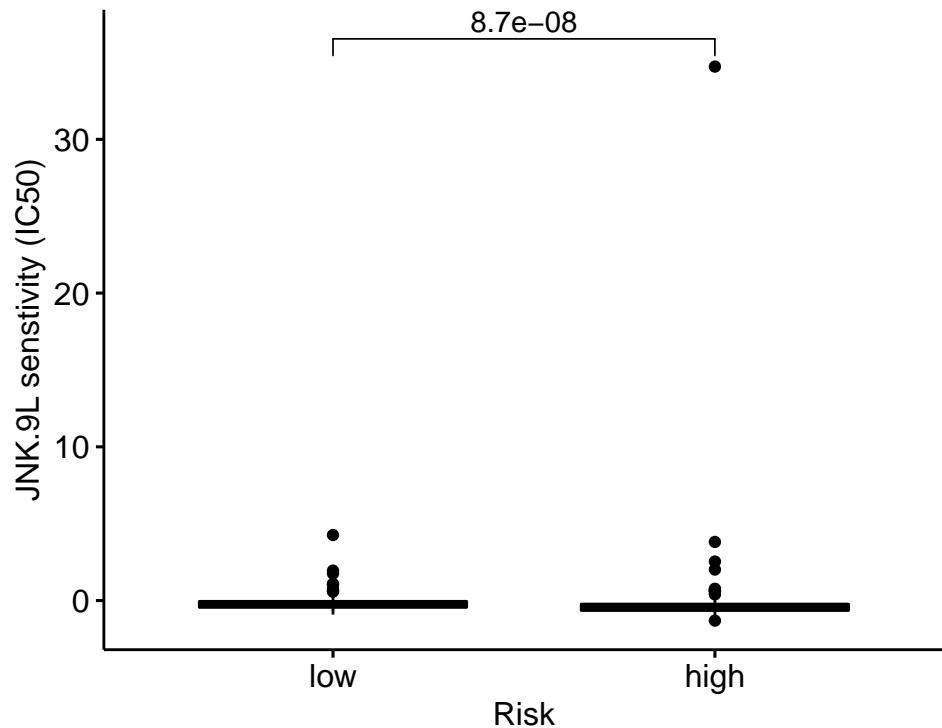

Supplement: Supplementary file 4 [file DataSheet_1.zip › Additional file 1-7/additional file 7. chemosensitivity/JNK.9L.pdf]

Risk 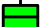 low 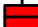 high

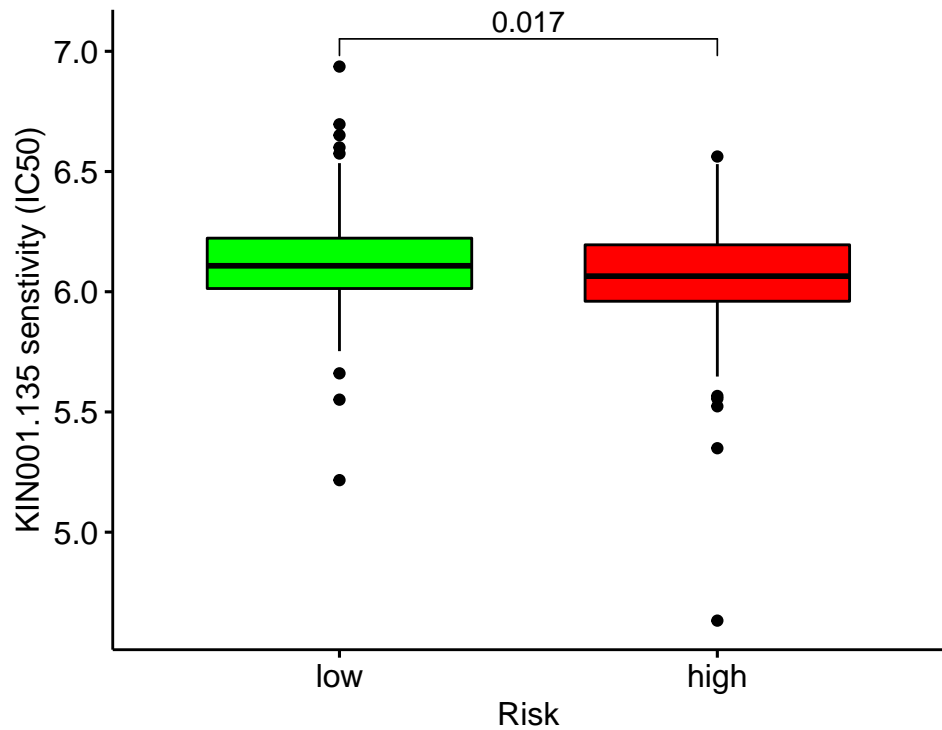

Supplement: Supplementary file 4 [file DataSheet_1.zip › Additional file 1-7/additional file 7. chemosensitivity/KIN001.135.pdf]

Risk 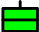 low 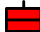 high

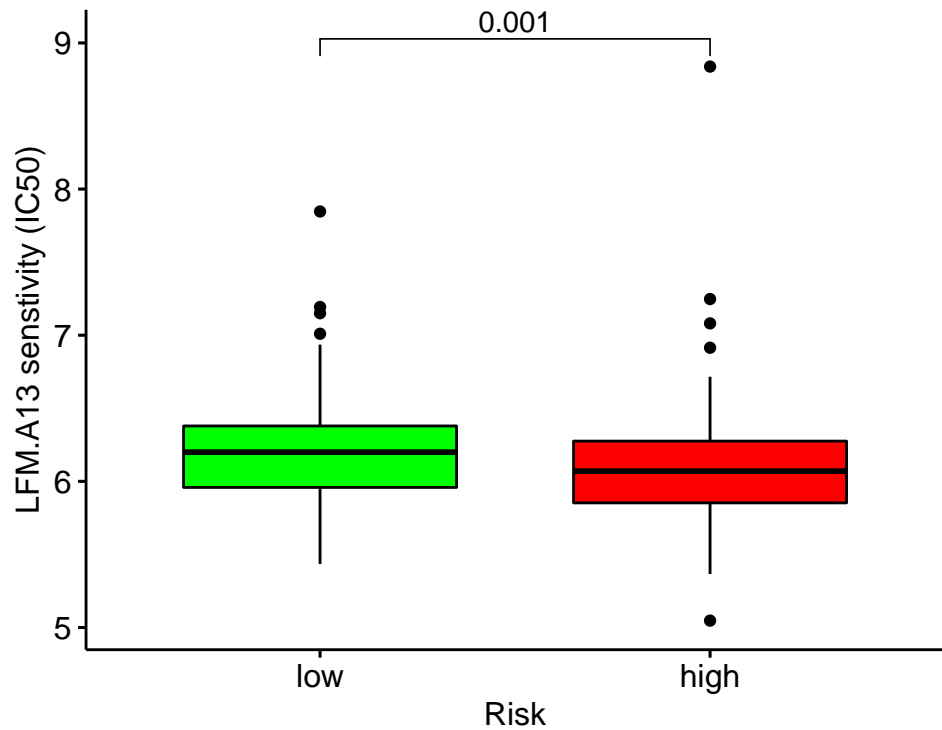

Supplement: Supplementary file 4 [file DataSheet_1.zip › Additional file 1-7/additional file 7. chemosensitivity/LFM.A13.pdf]

Risk 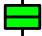 low 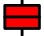 high

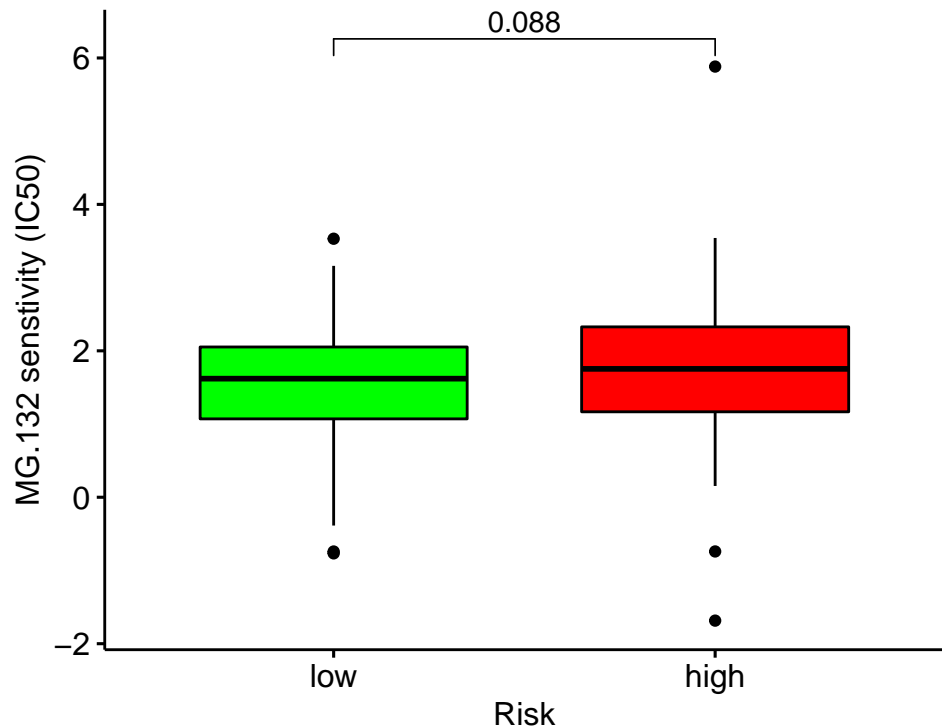

Supplement: Supplementary file 4 [file DataSheet_1.zip › Additional file 1-7/additional file 7. chemosensitivity/MG.132.pdf]

Risk 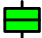 low 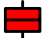 high

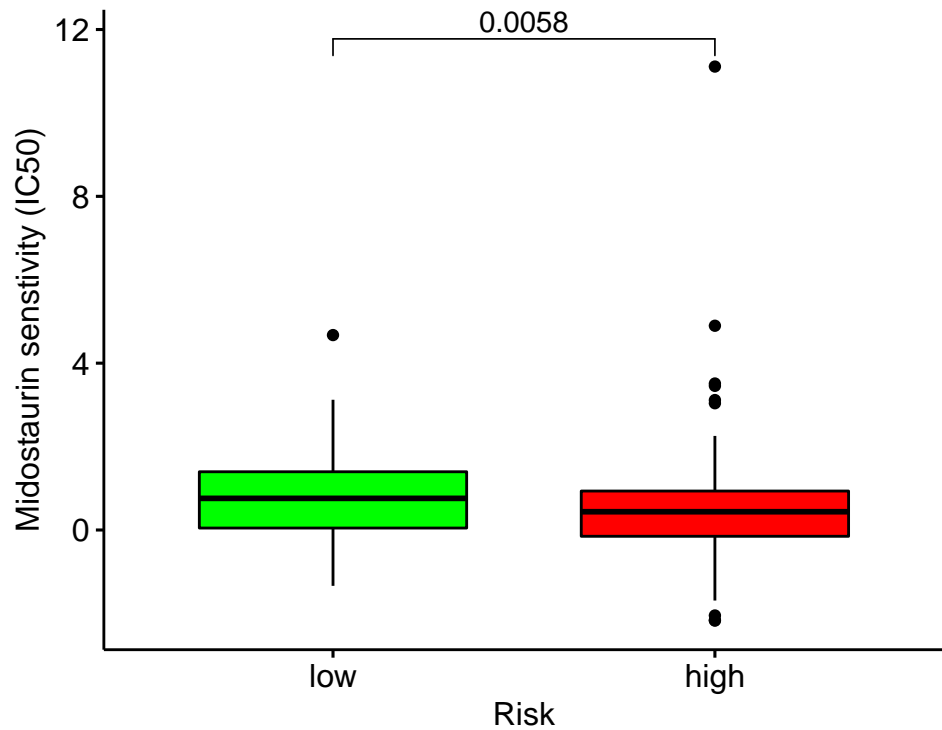

Supplement: Supplementary file 4 [file DataSheet_1.zip › Additional file 1-7/additional file 7. chemosensitivity/Midostaurin.pdf]

Risk 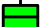 low 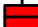 high

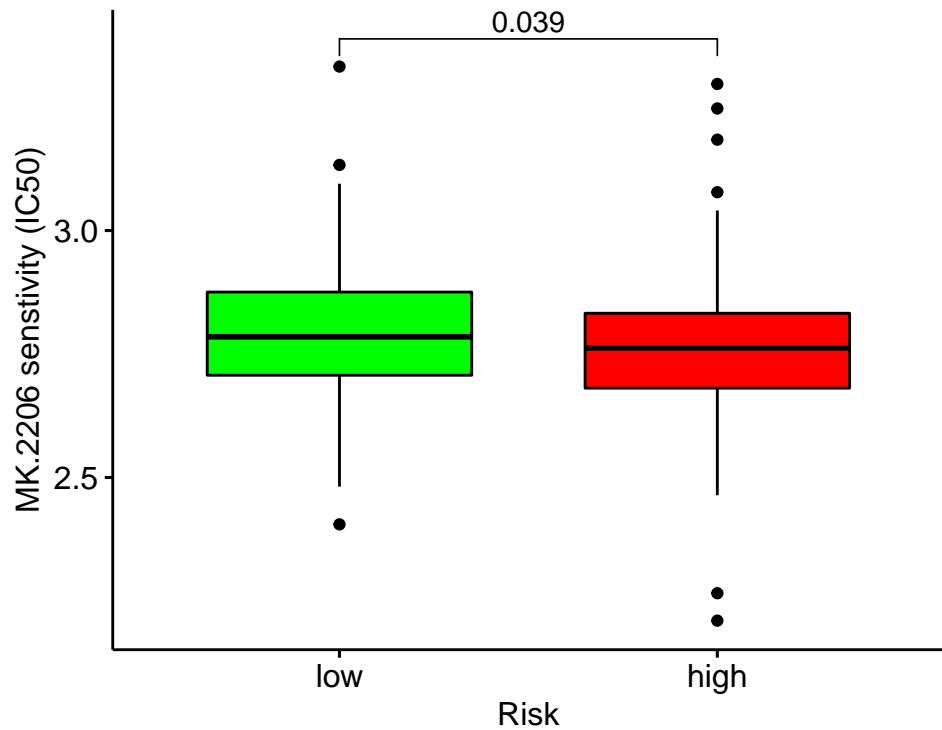

Supplement: Supplementary file 4 [file DataSheet_1.zip › Additional file 1-7/additional file 7. chemosensitivity/MK.2206.pdf]

Risk 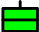 low 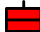 high

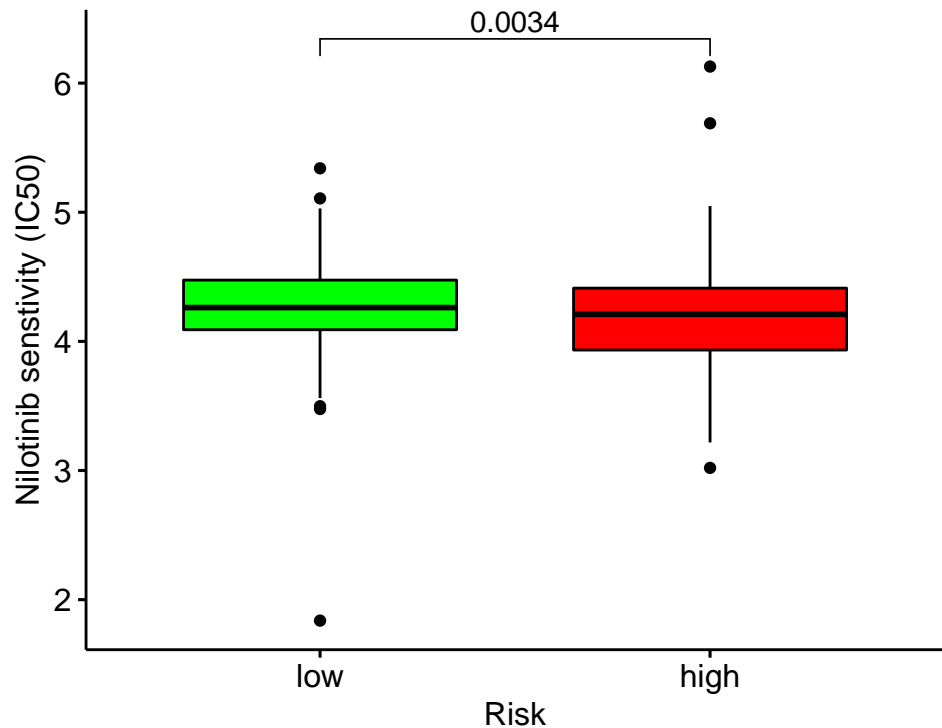

Supplement: Supplementary file 4 [file DataSheet_1.zip › Additional file 1-7/additional file 7. chemosensitivity/Nilotinib.pdf]

Risk 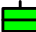 low 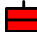 high

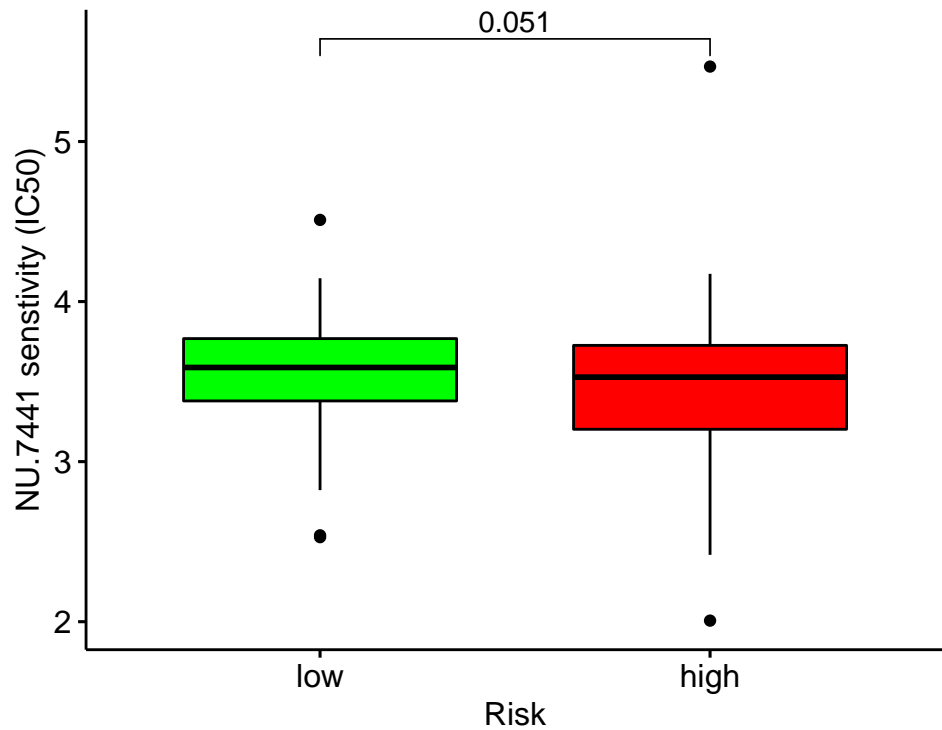

Supplement: Supplementary file 4 [file DataSheet_1.zip › Additional file 1-7/additional file 7. chemosensitivity/NU.7441.pdf]

Risk 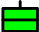 low 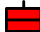 high

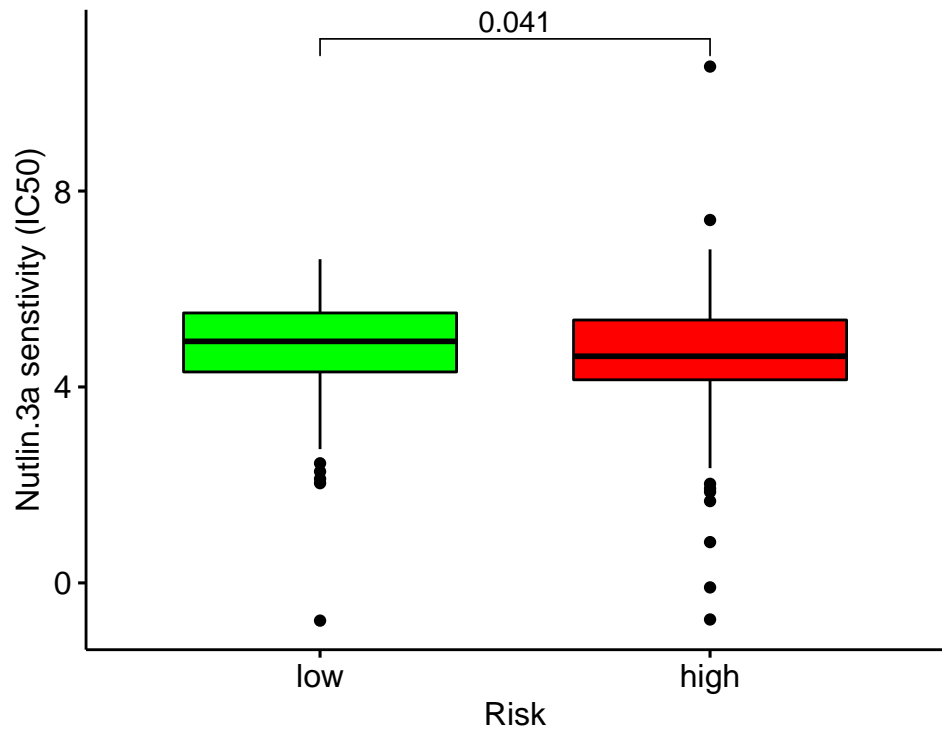

Supplement: Supplementary file 4 [file DataSheet_1.zip › Additional file 1-7/additional file 7. chemosensitivity/Nutlin.3a.pdf]

Risk 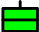 low 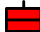 high

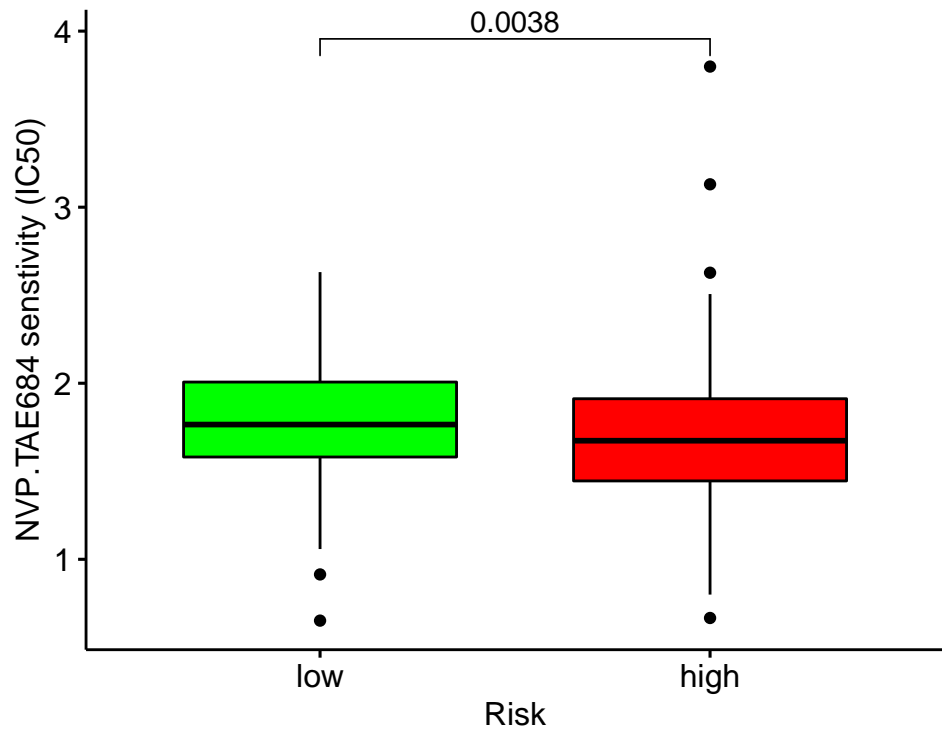

Supplement: Supplementary file 4 [file DataSheet_1.zip › Additional file 1-7/additional file 7. chemosensitivity/NVP.TAE684.pdf]

Risk 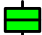 low 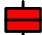 high

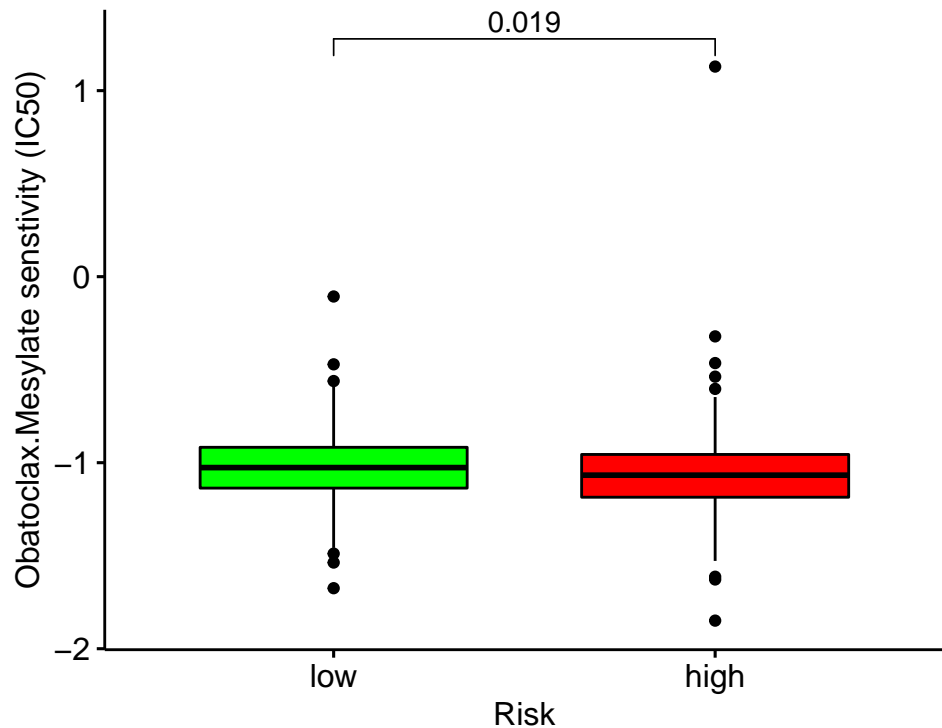

Supplement: Supplementary file 4 [file DataSheet_1.zip › Additional file 1-7/additional file 7. chemosensitivity/Obatoclax.Mesylate.pdf]

Risk 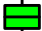 low 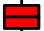 high

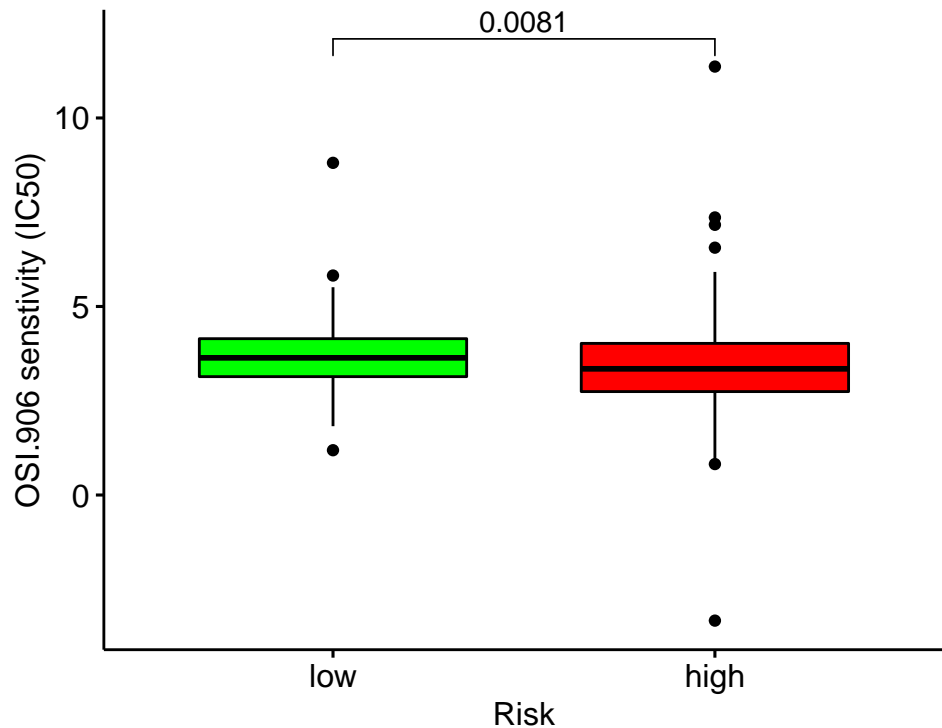

Supplement: Supplementary file 4 [file DataSheet_1.zip › Additional file 1-7/additional file 7. chemosensitivity/OSI.906.pdf]

Risk 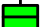 low 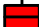 high

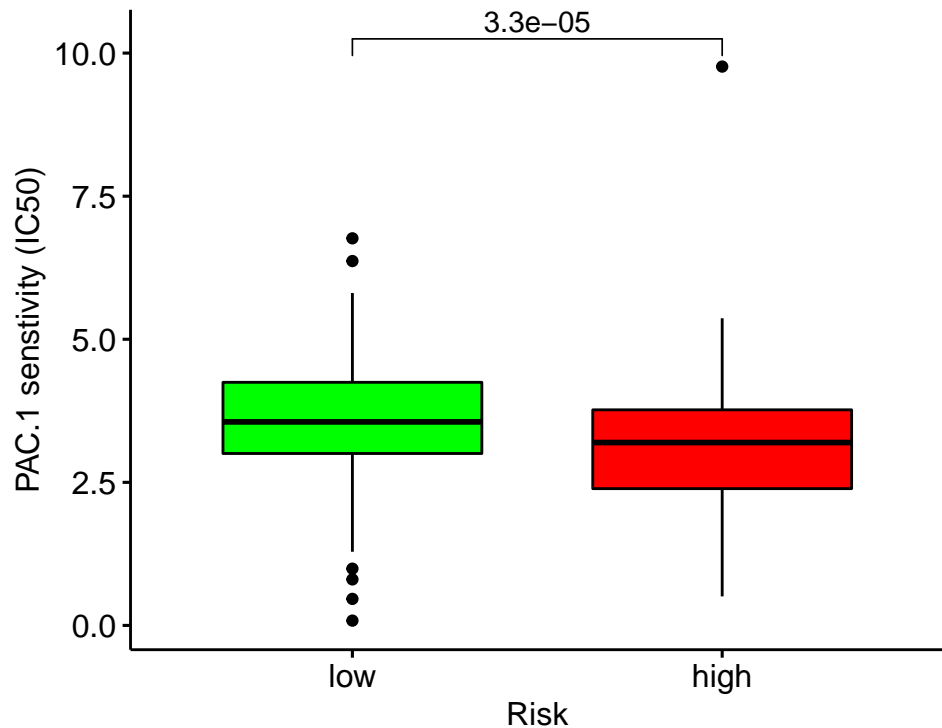

Supplement: Supplementary file 4 [file DataSheet_1.zip › Additional file 1-7/additional file 7. chemosensitivity/PAC.1.pdf]

Risk 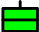 low 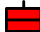 high

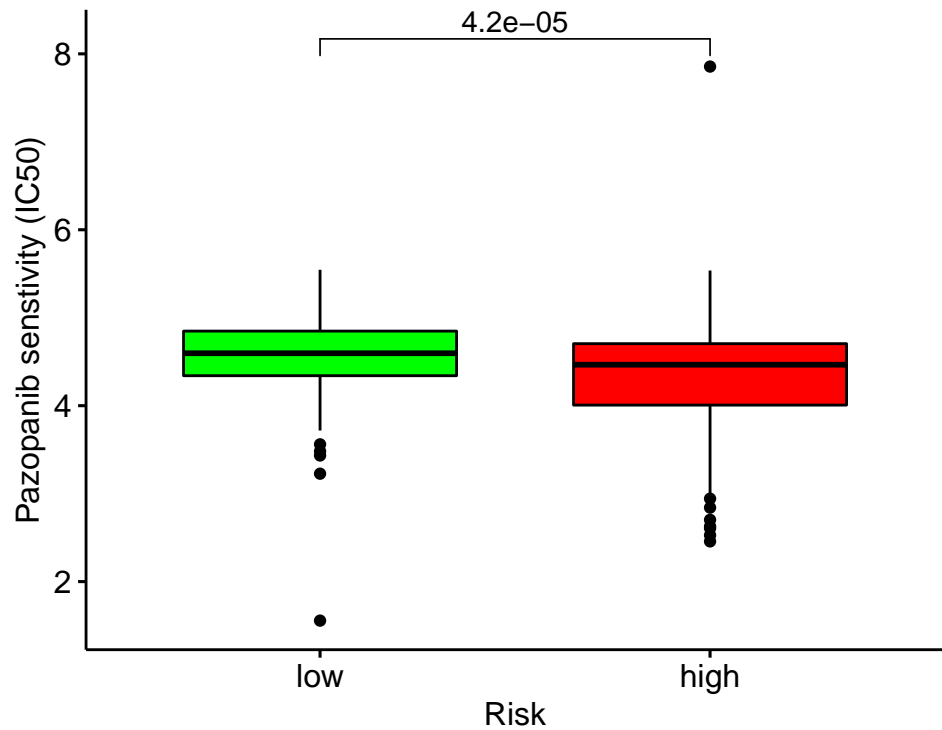

Supplement: Supplementary file 4 [file DataSheet_1.zip › Additional file 1-7/additional file 7. chemosensitivity/Pazopanib.pdf]

Risk 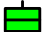 low 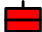 high

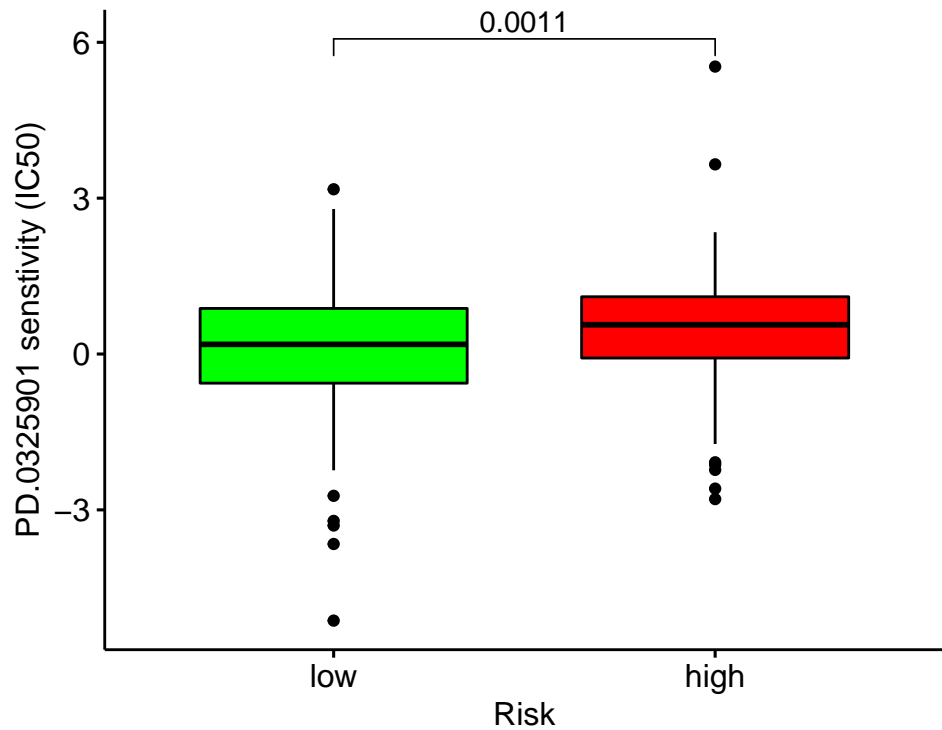

Supplement: Supplementary file 4 [file DataSheet_1.zip › Additional file 1-7/additional file 7. chemosensitivity/PD.0325901.pdf]

Risk 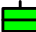 low 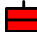 high

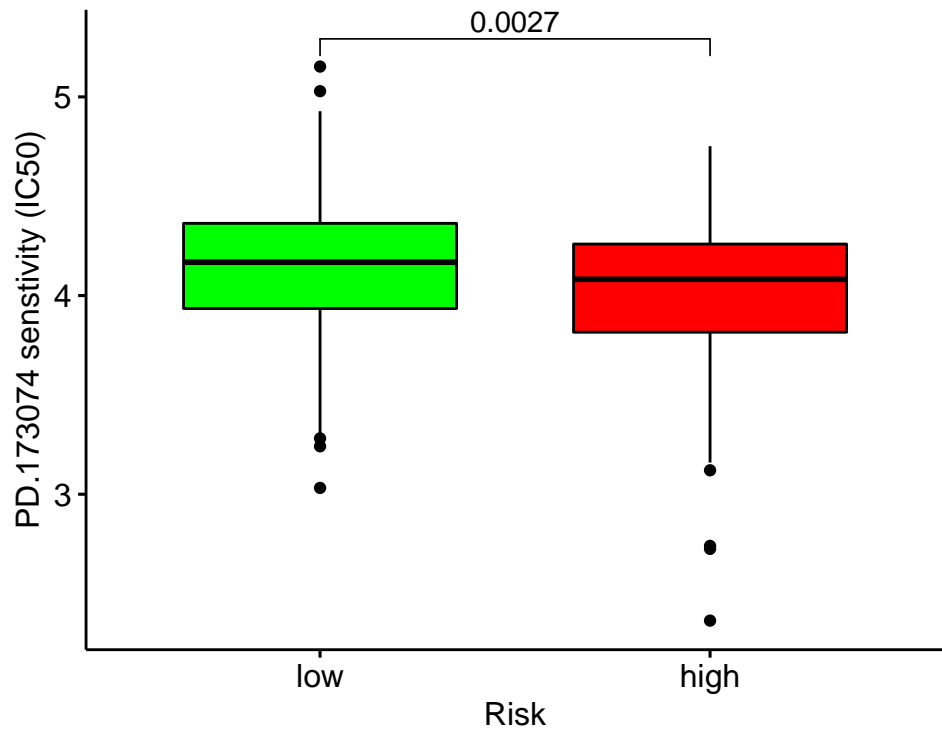

Supplement: Supplementary file 4 [file DataSheet_1.zip › Additional file 1-7/additional file 7. chemosensitivity/PD.173074.pdf]

Risk 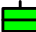 low 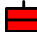 high

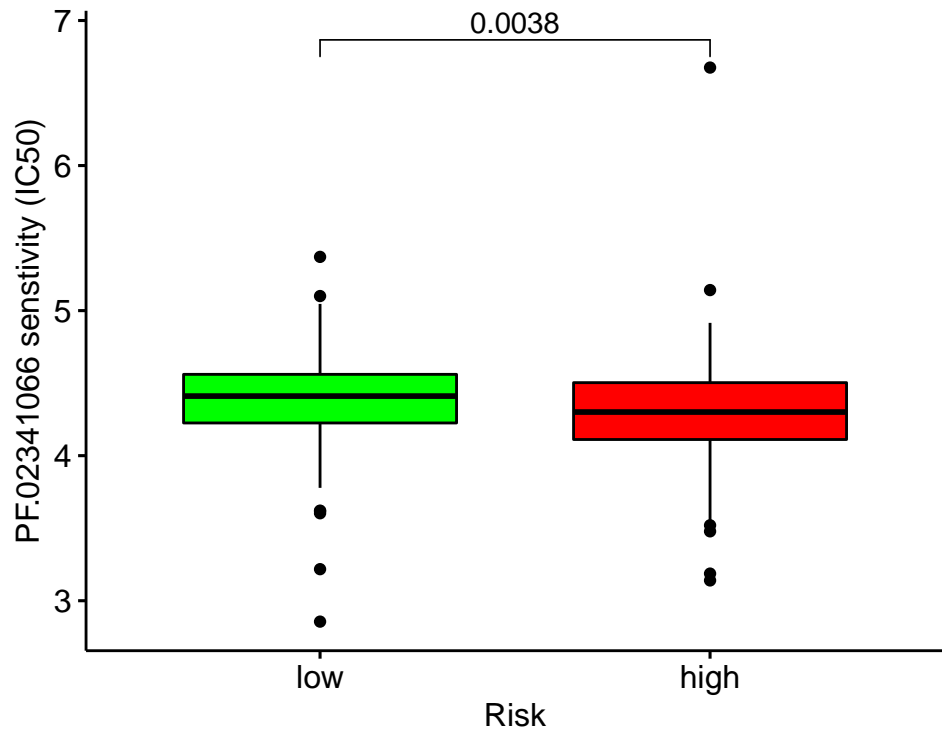

Supplement: Supplementary file 4 [file DataSheet_1.zip › Additional file 1-7/additional file 7. chemosensitivity/PF.02341066.pdf]

Risk 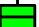 low 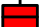 high

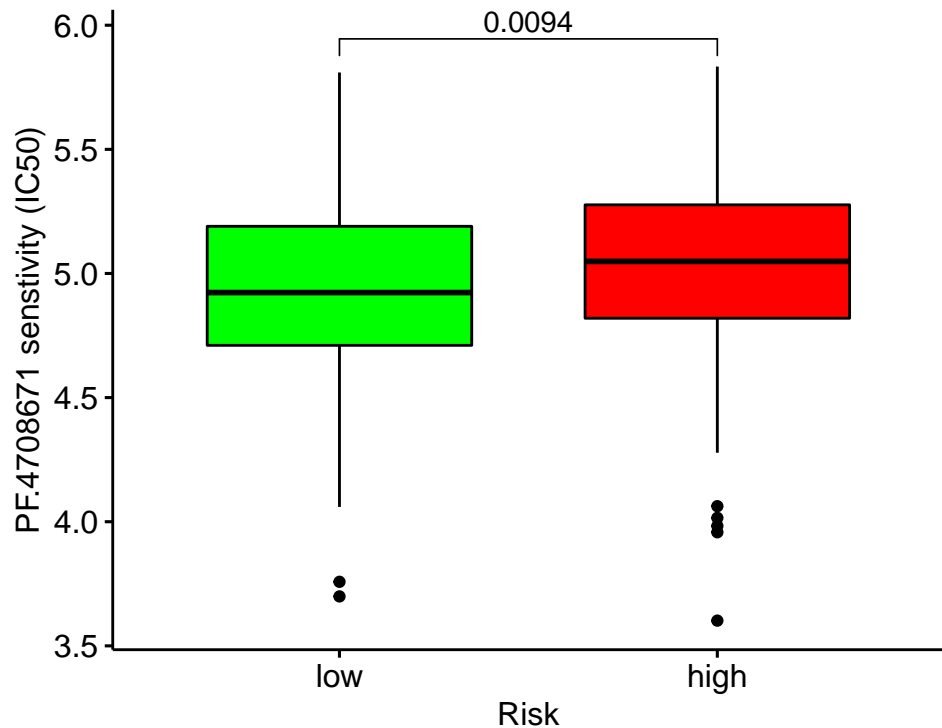

Supplement: Supplementary file 4 [file DataSheet_1.zip › Additional file 1-7/additional file 7. chemosensitivity/PF.4708671.pdf]

PF.562271 sensitivity (IC50)

Risk 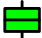 low 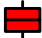 high

0.00028

low

high

Risk

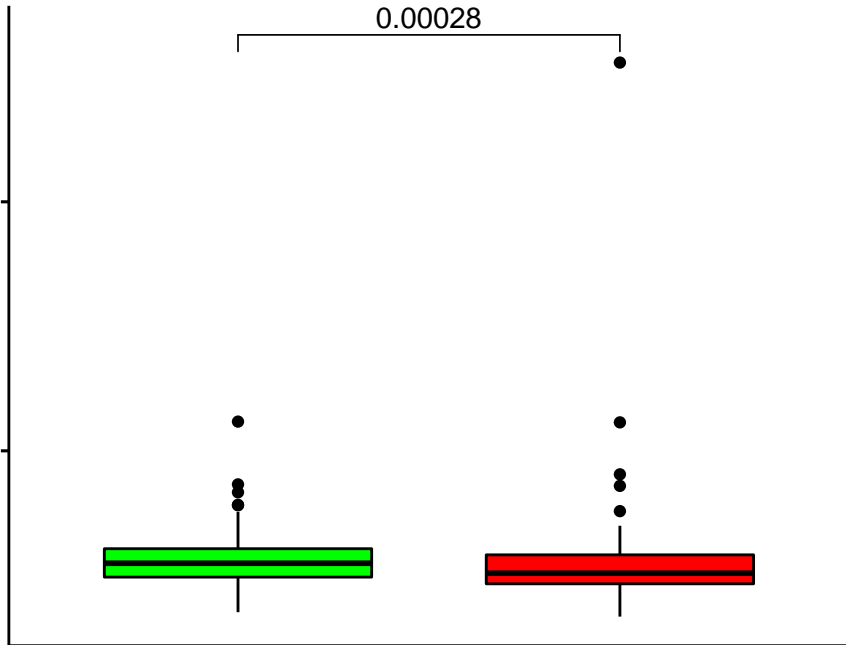

Supplement: Supplementary file 4 [file DataSheet_1.zip › Additional file 1-7/additional file 7. chemosensitivity/PF.562271.pdf]

Risk 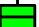 low 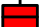 high

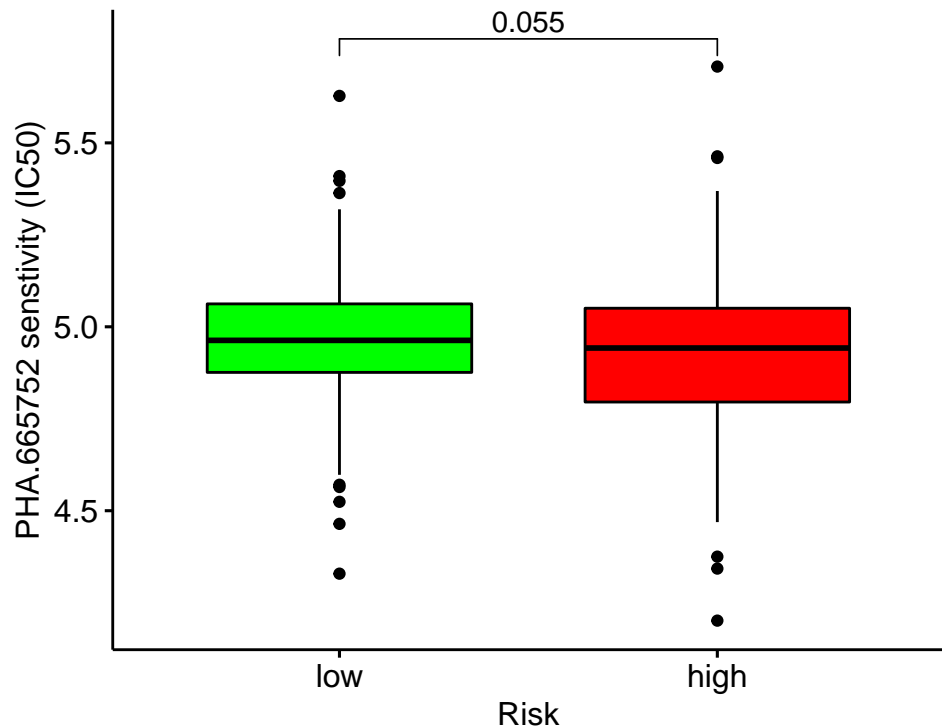

Supplement: Supplementary file 4 [file DataSheet_1.zip › Additional file 1-7/additional file 7. chemosensitivity/PHA.665752.pdf]

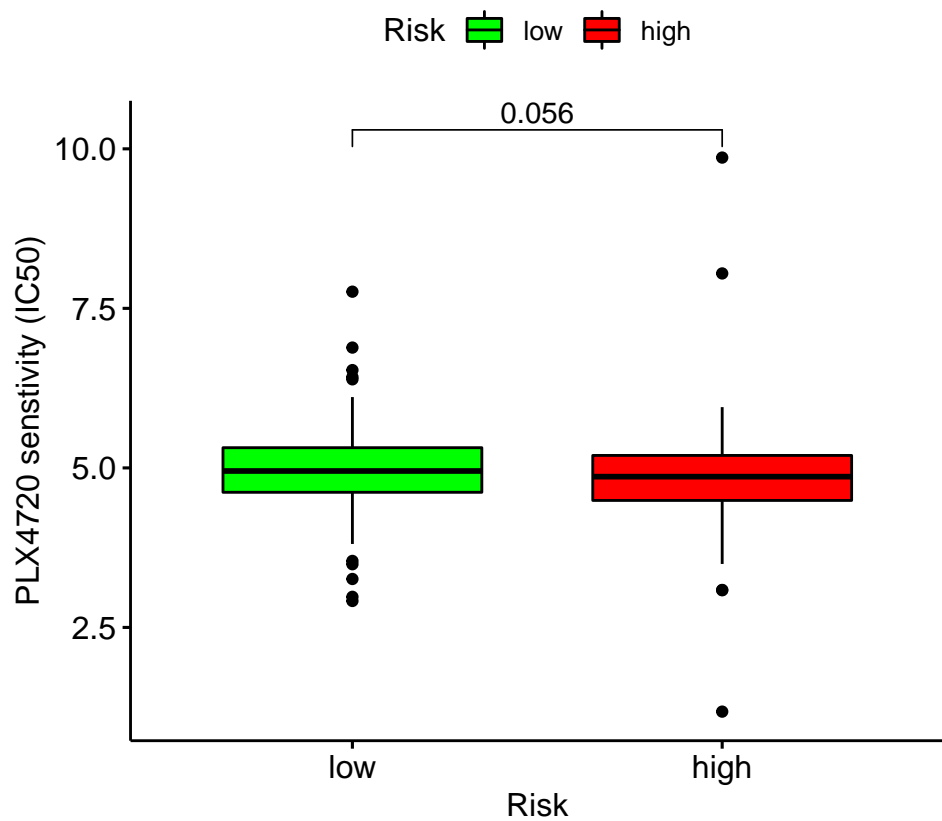

Supplement: Supplementary file 4 [file DataSheet_1.zip › Additional file 1-7/additional file 7. chemosensitivity/PLX4720.pdf]

Risk 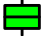 low 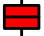 high

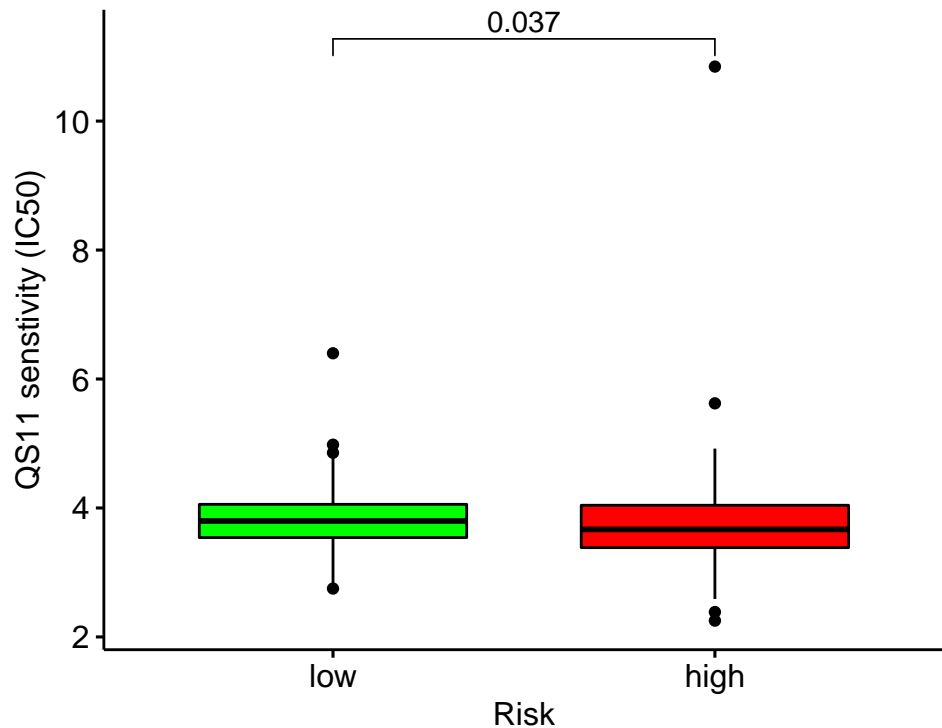

Supplement: Supplementary file 4 [file DataSheet_1.zip › Additional file 1-7/additional file 7. chemosensitivity/QS11.pdf]

Risk 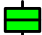 low 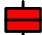 high

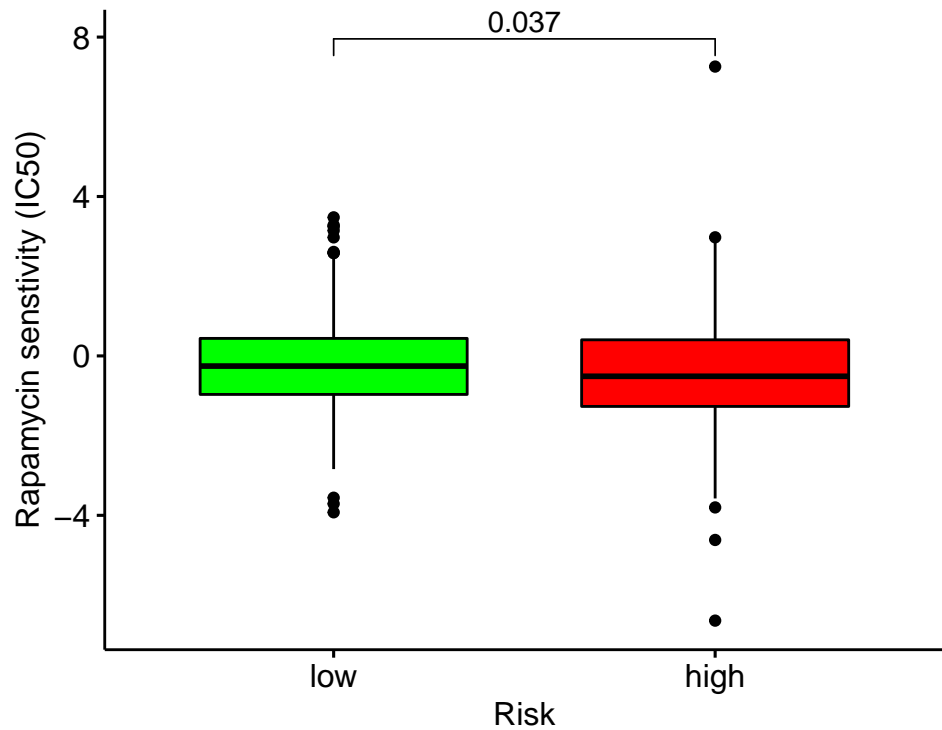

Supplement: Supplementary file 4 [file DataSheet_1.zip › Additional file 1-7/additional file 7. chemosensitivity/Rapamycin.pdf]

Risk 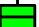 low 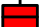 high

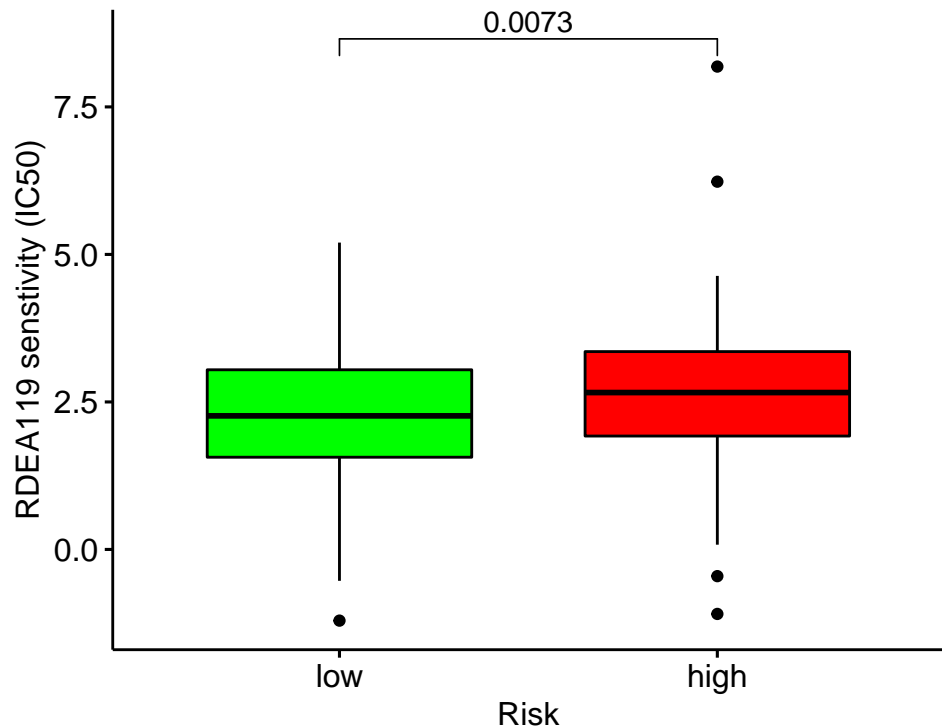

Supplement: Supplementary file 4 [file DataSheet_1.zip › Additional file 1-7/additional file 7. chemosensitivity/RDEA119.pdf]

Risk 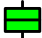 low 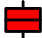 high

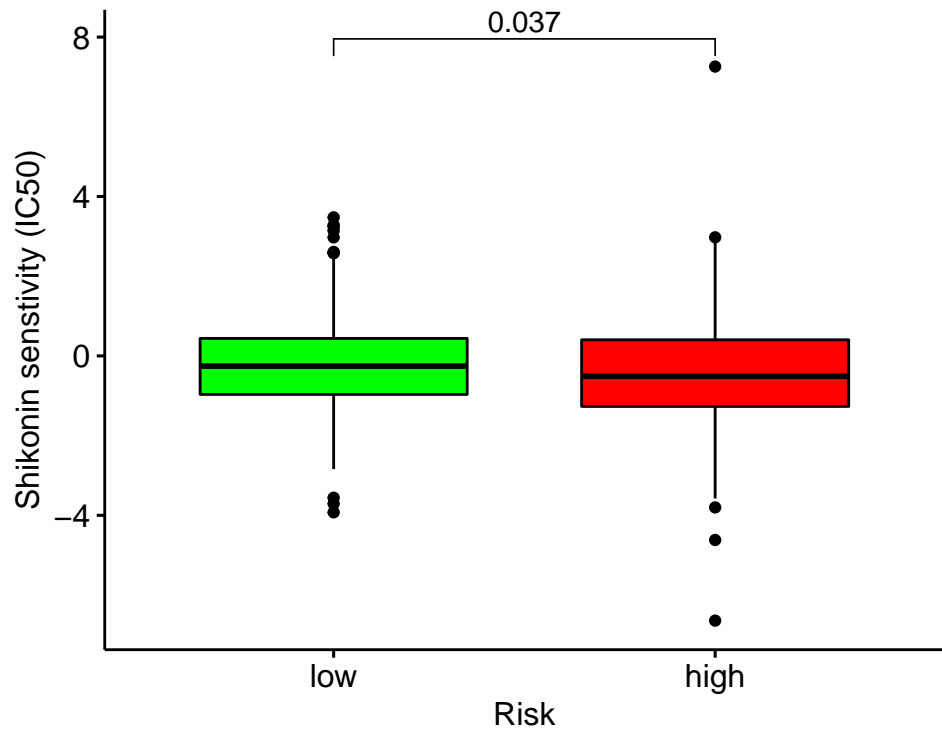

Supplement: Supplementary file 4 [file DataSheet_1.zip › Additional file 1-7/additional file 7. chemosensitivity/Shikonin.pdf]

Thapsigargin sensitivity (IC50)

Risk 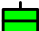 low 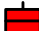 high

0.0034

20  
10  
0  
-10

low

high

Risk

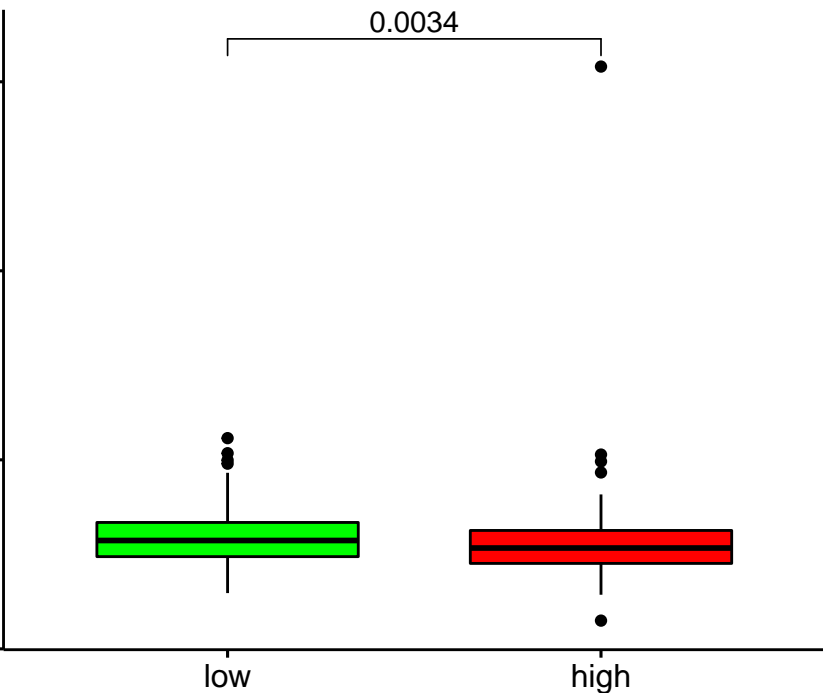

Supplement: Supplementary file 4 [file DataSheet_1.zip › Additional file 1-7/additional file 7. chemosensitivity/Thapsigargin.pdf]

Risk 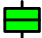 low 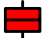 high

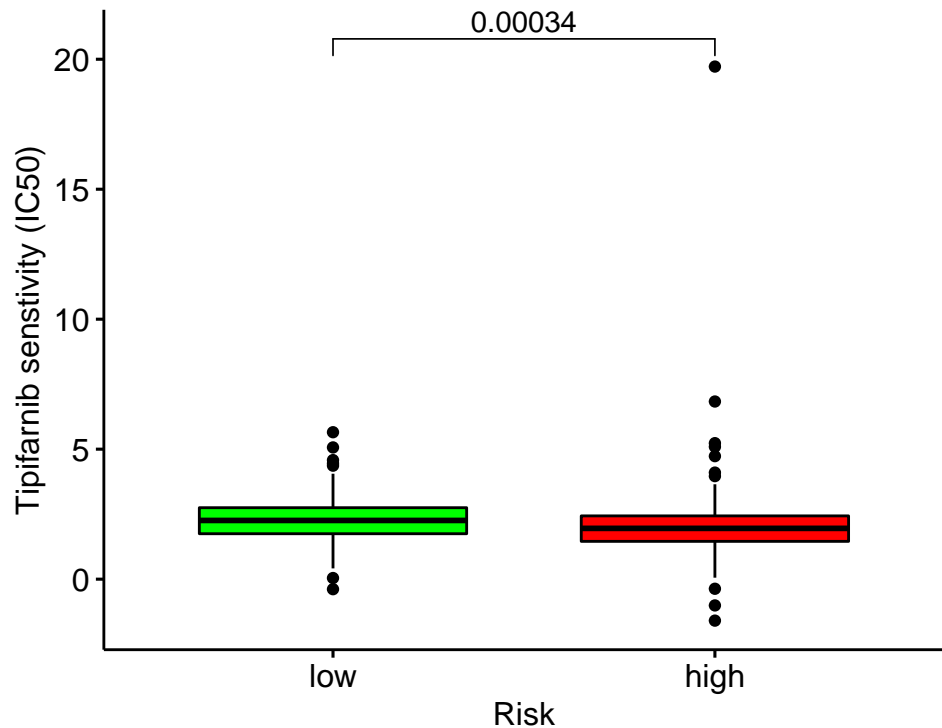

Supplement: Supplementary file 4 [file DataSheet_1.zip › Additional file 1-7/additional file 7. chemosensitivity/Tipifarnib.pdf]

Risk 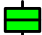 low 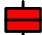 high

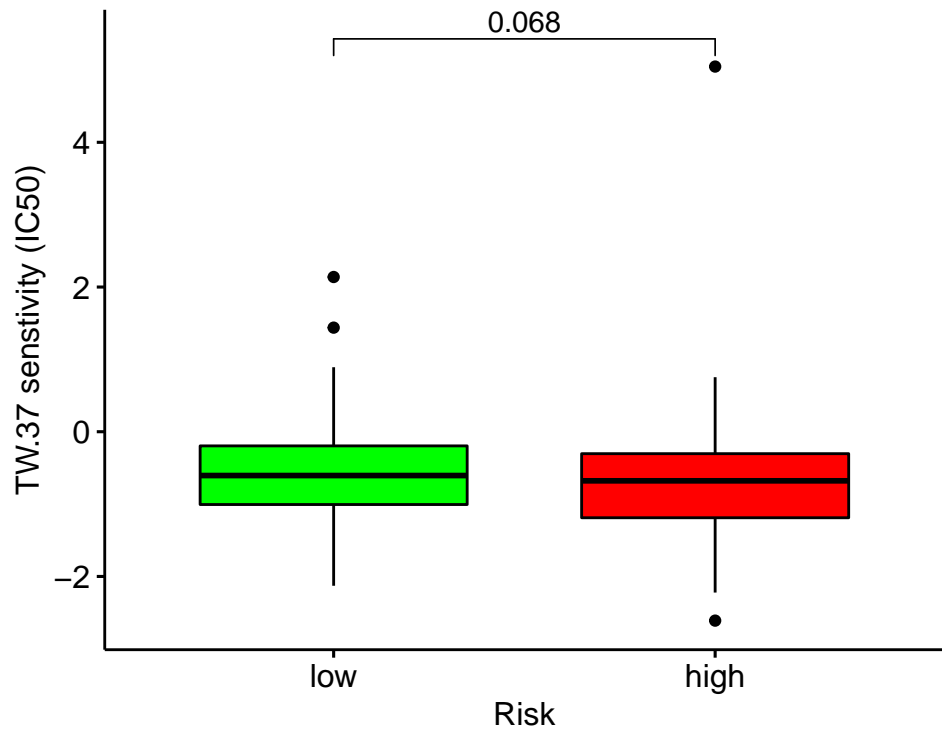

Supplement: Supplementary file 4 [file DataSheet_1.zip › Additional file 1-7/additional file 7. chemosensitivity/TW.37.pdf]

Risk 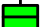 low 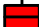 high

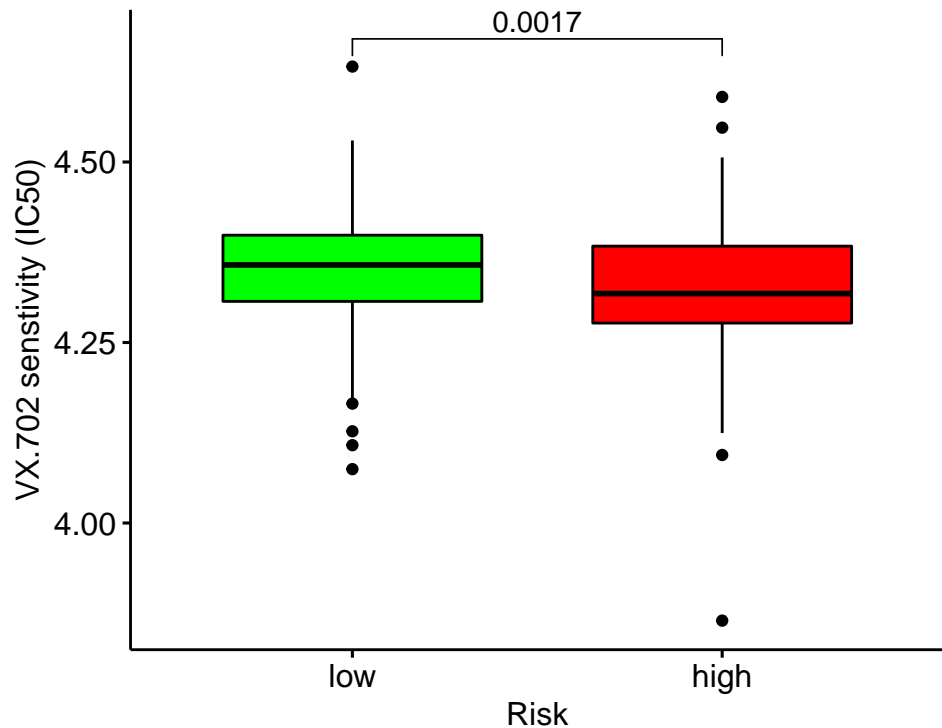

Supplement: Supplementary file 4 [file DataSheet_1.zip › Additional file 1-7/additional file 7. chemosensitivity/VX.702.pdf]

Risk 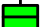 low 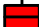 high

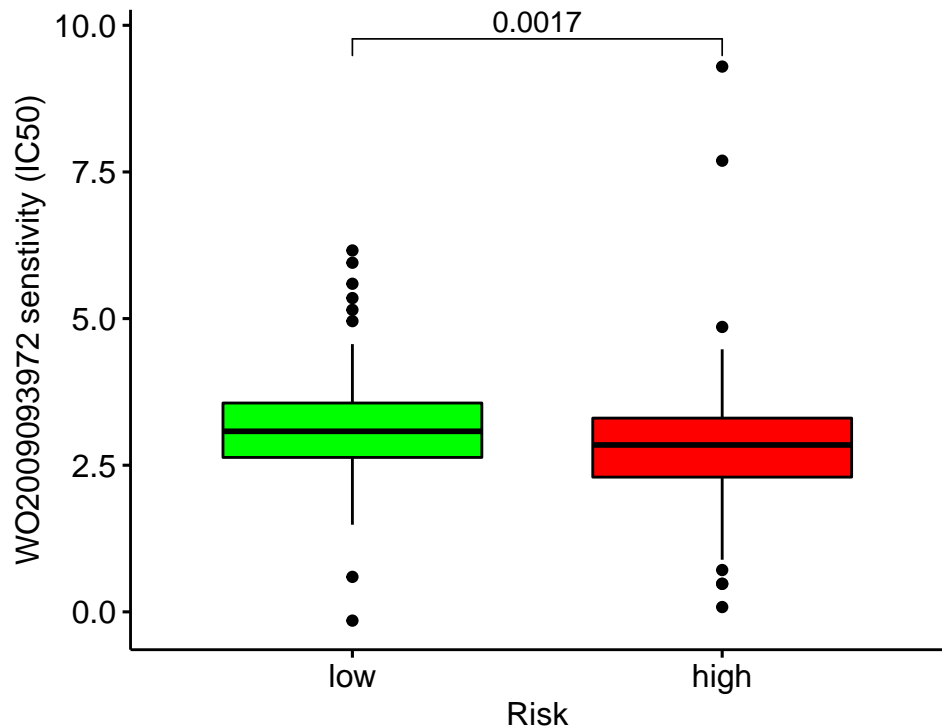

Supplement: Supplementary file 4 [file DataSheet_1.zip › Additional file 1-7/additional file 7. chemosensitivity/WO2009093972.pdf]

Risk 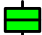 low 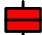 high

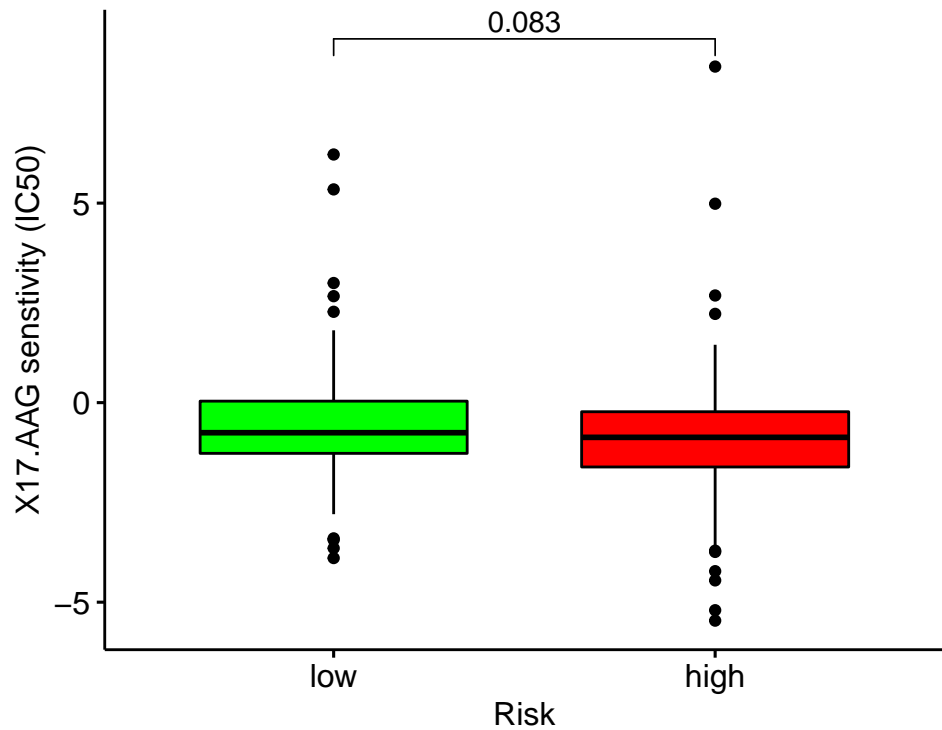

Supplement: Supplementary file 4 [file DataSheet_1.zip › Additional file 1-7/additional file 7. chemosensitivity/X17.AAG.pdf]

Risk 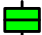 low 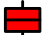 high

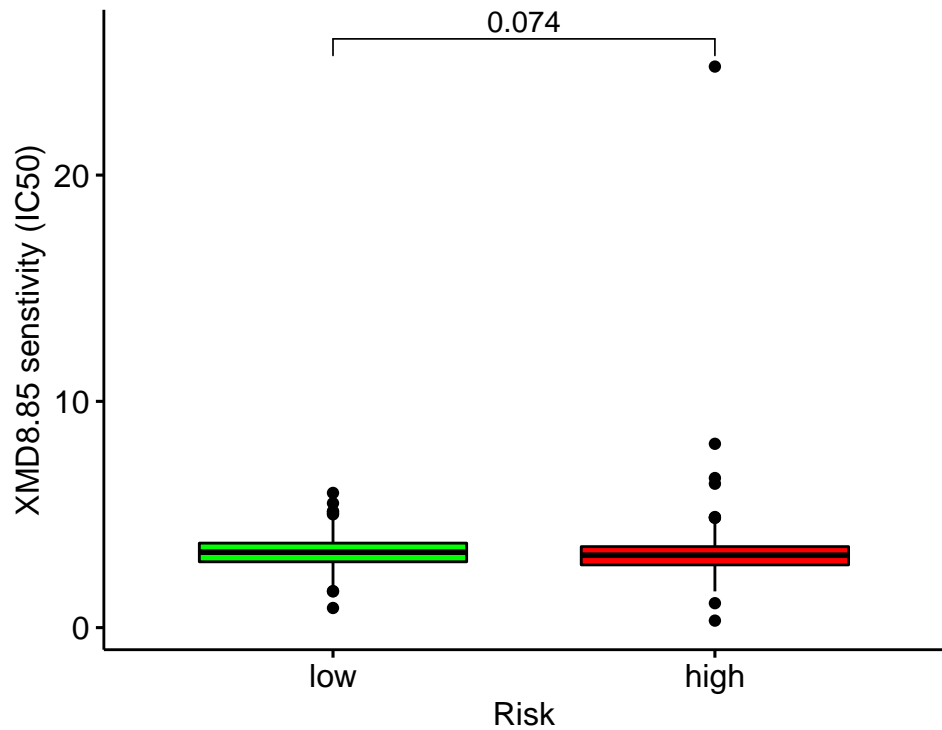

Supplement: Supplementary file 4 [file DataSheet_1.zip › Additional file 1-7/additional file 7. chemosensitivity/XMD8.85.pdf]

Risk 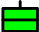 low 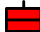 high

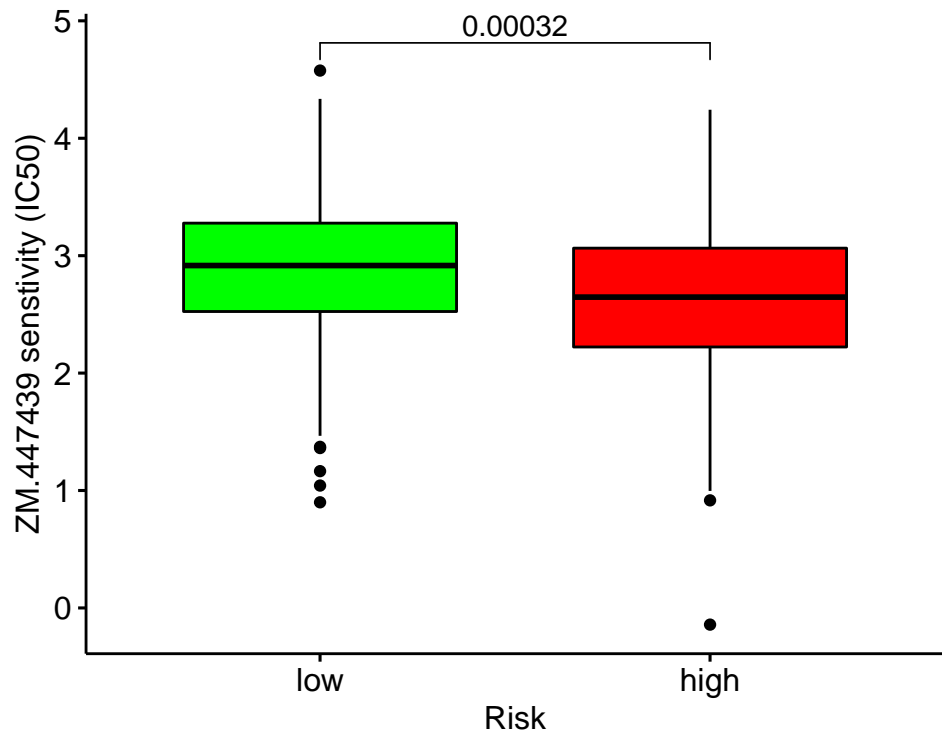

Supplement: Supplementary file 4 [file DataSheet_1.zip › Additional file 1-7/additional file 7. chemosensitivity/ZM.447439.pdf]
