# Supplementary material for: Identification and Validation of Novel Immune-Related Alternative Splicing Signatures as a Prognostic Model for Colon Cancer
Source: Front Oncol. 2022 May 26;12:866289. doi: 10.3389/fonc.2022.866289 (PMC9178000; doi:10.3389/fonc.2022.866289)
Supplement: Supplementary file 3 [file DataSheet_1.docx]

**Supplemental Materials**

**Primers for qRT-PCR**

| Transcript | Forward | Reverse |
| --- | --- | --- |
| CD46^△13^ | GCAGTAATTTGTGTTGTCCCGT | GCTCTGCTGGAGTGGTTGAT |
| CD46^13+^ | ACCTAACTGATGAGACCCACAGA | AGTGGCATATTCAGCTCCACC |
| PSMC5^△2.1^ | TTGACGGACCAGAGCAGATG | TGGACAGATAATATTGGCGGAGT |
| PSMC5^2.1+^ | TCATATCGCCTCTCGGTCCT | TGGACAGATAATATTGGCGGAGT |
|  |  |  |

**siRNA sequence**

**CD46^△13^ siRNA#1**

GGAGGAAGAAGAAAGGGAA

UUCCCUUUCUUCUUCCUCC

**CD46^△13^ siRNA#2**

GGAGGAAGAAGAAAGGGAAAG

UUCCCUUUCUUCUUCCUCCUU

**CD46^13+^ siRNA #1**

GAAGGAGAGAUGAGAGAAAGG

UUUCUCUCAUCUCUCCUUCUC

**CD46^13+^ siRNA #2**

GAGAAGGAGAGAUGAGAGAAA

UCUCUCAUCUCUCCUUCUCAG

**PSMC5**^△2.1^ **siRNA#1**

CAGAGCAGAUGGAGCUGGAGG

UCCAGCUCCAUCUGCUCUGGU

**PSMC5**^△2.1^ **siRNA#2**

CAGAGCAGAUGGAGCUGGA

UCCAGCUCCAUCUGCUCUG

**PSMC5^2.1+^ siRNA#1**

GGGCUUGUAGAGCACUUCACG

UGAAGUGCUCUACAAGCCCGA

**PSMC5^2.1+^ siRNA#2**

GGGAGAGGAAGACGCGGUAGA

UACCGCGUCUUCCUCUCCCAG
